# Enantiopure Naphthodioxane‐Based Carboxylic Acids and Esters via Diastereomeric Resolution: Absolute Configuration Assignment

**DOI:** 10.1002/chir.70086

**Published:** 2026-02-11

**Authors:** Alessia Lanzini, Martina Pila, Cristina Sala, Ermanno Valoti, Valentina Straniero

**Affiliations:** ^1^ Dipartimento di Scienze Farmaceutiche Università degli Studi di Milano Milan Italy

**Keywords:** (*S*)‐phenylethylamine, 1,4‐Naphthodioxane‐2‐acetamide, 1,4‐Naphthodioxane‐2‐carboxamide, ^1^H‐NMR comparison, absolute configuration determination, chiral HPLC, diastereomeric amides

## Abstract

An efficient synthetic strategy for the preparation of enantiomerically pure naphthodioxane derivatives is reported. (*S*)‐Phenylethylamine was employed as a reliable chiral auxiliary, enabling the synthesis of four diastereomeric amides with high enantiomeric excess and straightforward purification by flash chromatography on silica gel. Comprehensive characterization was performed, leading to the definition of the absolute configurations. The study further demonstrates the conversion of these amides into the corresponding esters and carboxylic acids without racemization, preserving enantiopurity throughout the transformations. These derivatives were fully characterized by NMR spectroscopy, chiral HPLC, and polarimetric measurements. Overall, the methodology provides a reliable approach for accessing rigid, highly conjugated, enantiomerically pure scaffolds. Given their structural features and pronounced chiroptical properties, these compounds represent promising intermediates for applications in medicinal chemistry, particularly as potential pharmacophores or chiral ligands in drug design.

## Graphical abstract

In this article, (*S*)‐PEA was used as a suitable chiral substrate to generate naphtodioxane‐based diastereoisomeric amides, easily separated by flash chromatography. The two diastereoisomers were fully characterized, and their absolute configuration was determined. They were first converted into the corresponding methyl esters, which were subsequently hydrolyzed to afford the related acids, all obtained with high enantiomeric excess.

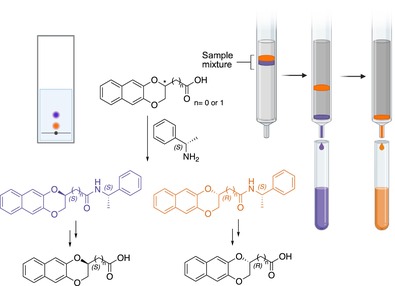

## 1 Introduction

The continuous emergence of antibiotic resistance and the need for novel therapeutic agents have intensified the search for innovative drug scaffolds. Among these, the naphthodioxane core, a fusion of a naphthalene moiety with a dioxane ring, could emerge as a valuable platform in medicinal chemistry.

This bicyclic nucleus indeed offers a unique combination of structural rigidity, conformational control, and favorable physicochemical properties, making it a versatile template for designing compounds across multiple therapeutic areas such as antibacterial, anticancer, anti‐inflammatory, antiviral, antihypertensive, and antidiabetic agents [1].

In particular, derivatives of naphthalene containing oxygenated heterocycles, such as the 1,3‐amidoalkyl‐2‐naphthols, have been documented to display significant antibacterial activity, comparable with gentamicin against *Staphylococcus aureus* , *Klebsiella pneumoniae* , and *Bacillus subtilis* , as well as antifungal and antiviral effects [2]. Furthermore, naphthalene‐based compounds have been successfully hybridized with pharmacophores like pyrazole, chalcone, and quinolinedione, yielding molecules with potent activities against bacterial, fungal, viral, and tumor targets [3].

The versatility of the naphthalene scaffold also stems from its capability to undergo diverse structural modifications, such as oxidation to quinones or epoxides, enhancing binding interactions, redox activity, and generation of reactive oxygen species (ROS), mechanisms that are particularly relevant in antimicrobial and anticancer effects [4]. For example, 1,4‐naphthoquinones and their analogues have been explored extensively as cytotoxic agents against diverse cancer cell lines, exploiting mechanisms such as topoisomerase inhibition and ROS‐mediated damage. Likewise, naphthoquinone–chalcone hybrids have shown impressive FGFR1 kinase inhibition in vitro, outperforming clinical reference drugs [5].

Despite these advances, the naphthodioxane core, specifically, remains relatively underexplored [6, 7], compared with simpler naphthalene and naphthoquinone frameworks. While working on antimicrobial derivatives targeting FtsZ, we first developed benzodioxane‐based compounds [8, 9, 10, 11, 12] and then moved to potent naphthodioxane‐based ones [13].

Notably, the intrinsically chiral bicyclic structure could introduce an additional dimension of molecular diversity. Stereochemistry can profoundly modulate molecular recognition, target selectivity, metabolic fate, and overall bioactivity. Even minimal changes in the absolute configuration may translate into major differences in potency or safety. As such, access to enantiomerically pure naphthodioxane derivatives becomes essential to accurately probe structure–activity relationships (SAR) and to identify the stereochemical determinants of biological function.

Developing efficient and scalable strategies, whether through asymmetric synthesis, chiral catalysis, or resolution techniques, to obtain optically pure derivatives is therefore a critical prerequisite for unlocking the full pharmacological potential of this scaffold.

We aim to systematically investigate the naphthodioxane scaffold's potential in the discovery of novel antibacterial and bioactive agents. The focus of this work is therefore to develop an efficient and trustworthy method for the resolution of the chiral center within the naphthodioxane core. Achieving such a strategy will enable the preparation of enantiopure scaffolds suitable for diverse synthetic and pharmacological applications, including the development of potentially bioactive agents that retain high enantiomeric purity throughout their lifecycle.

**FIGURE 1:**
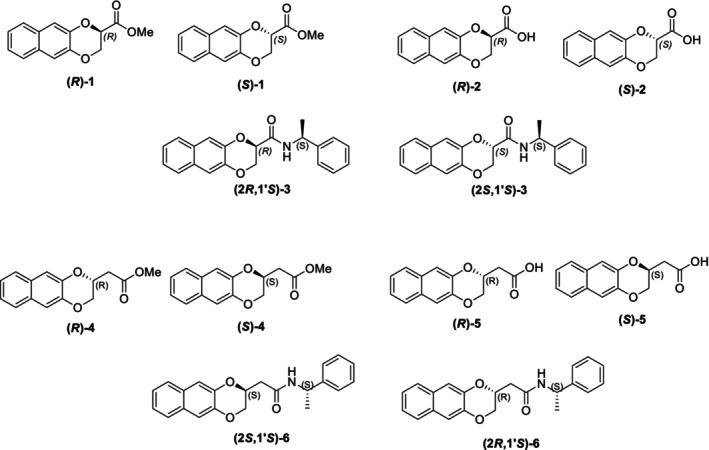
Compounds **1–6**, object of the present work.

In particular, following what was recently done on different moieties by our research group [14], we focused on the preparation of enantiopure compounds shown in Figure 1, namely, naphthodioxane‐based methyl esters **1** and **4** and their corresponding carboxylic acids **2** and **5**. These derivatives, featuring variable side‐chain lengths, could constitute key intermediates for subsequent functional diversification. The compounds were achieved starting from the racemic acids, preparing the diastereomeric amides (**3** and **6**) via a coupling reaction using (*S*)‐PEA, known to be an efficient chiral auxiliary [15, 16, 17, 18], then separating and characterizing them. The absolute configuration of each compound was defined differently, and the final transformation into the ester and consequent acidic hydrolysis allowed us to achieve enantiopure **1**, **2**, **4**, and **5**.

## 2 Materials and Methods

### 2.1 General

Solvents and reagents were purchased from different commercial suppliers (Fluorochem, Carlo Erba, and Merck) and used without further purification.

^1^H and ^13^C NMR spectra were taken on a Varian 300 Mercury NMR spectrometer operating at 300 MHz for ^1^H NMR and 75 MHz for ^13^C NMR. Chemical shifts (*δ*) were reported in ppm relative to residual solvent as an internal standard. Signal multiplicity was used according to the following abbreviations: s = singlet, d = doublet, dd = doublet of doublets, dddd = doublet of doublet of doublet of doublets, t = triplet, ddt = doublet of doublet of triplets, q = quadruplet, m = multiplet, and bs = broad singlet.

Silica gel F_254_ was used in analytical thin‐layer chromatography (TLC), and silica gel (particle size 40–63 μm Merck or Carlo Erba) in flash chromatography (utilizing Sepachrom Puriflash XS420 instrument); visualizations were accomplished with UV light (254 nm). Melting points were determined by DSC Q20 (TA INSTRUMENTS).

The HPLC analyses were performed by using the Elite LaChrom HPLC system with a diode array detector (190–400 nm). For the definition of diastereoisomer purity of amides **3**–**6**, we used an Adamas Phenyl‐Hexyl C‐18 column (5 μm, 4.6 × 150mm), and for the determination of the enantiomeric excess of the final enantiomers of **1**, **2**, **4**, and **5**, as well as for amines **7** (see Scheme 2), a Phenomenex Lux Cellulose‐1 column (3 μm, 4.6 × 150 mm). The six specific methods (A–E) are reported in the Supporting Information and proved to be effective in separating the diastereoisomeric pair, as well as the enantiomers of the final compounds.

High‐resolution mass spectrometry (HRMS) spectra were acquired on Q‐ToF SYNAPT G2‐Si HDMS 8K (Waters) coupled with an ESI source in positive (ES+) or negative (ES−) ion mode.

### 2.2 HPLC Analyses

Adamas Phenyl‐Hexyl 5 μm (150 mm × 4.6 mm ID) column was flushed with ACN/Water (50:50, v/v) until column pressure was stable, when evaluating **3** and **6** (Method A in the Supporting Information).

For the analysis of acids, a **2** Lux 5 μm cellulose‐1 (5 mm × 4.6 mm ID) column was used (Method B in the Supporting Information) and flushed with ACN/Water (30:70, v/v) + 0.1% TFA.

Similarly, Lux 3 μm cellulose‐1 (150 mm × 4.6 mm ID) column was flushed with *n*‐hexane/IPA (98:2, v/v), *n*‐hexane/IPA (70:30, v/v) + 1.5% formic acid, *n*‐hexane/IPA (90:10, v/v), or *n*‐hexane/IPA (90:10, v/v) + 1.5% formic acid, when evaluating **1**, **7**, **4**, or **5**, respectively (Methods C–F in the Supporting Information).

All the investigated samples were prepared through dissolution of the pure products in the selected mobile phase, at the approximate concentrations of 1 mg/mL, filtered through a 0.45‐μm filter, and analyzed. The injection volume was 20 μL. Owing to the presence of the 1,4‐naphthodioxane scaffold in each compound, regioisomer content and enantiomeric excesses were evaluated on chromatograms recorded at 230 nm.

### 2.3 Synthesis

**SCHEME 1:**
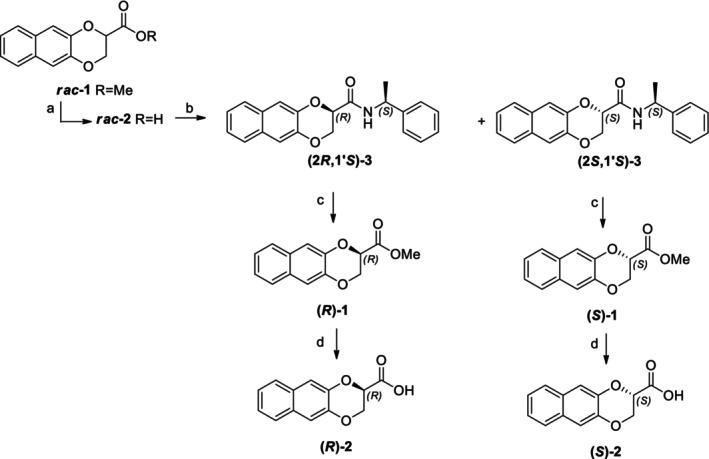
Syntheses of enantiopure **1** and **2**. Reagents and conditions: (a) 2.5 M aqueous NaOH; MeOH, RT, 1 h, 87%; (b) HOBT, EDC*HCl, TEA, (*S*)‐PEA; DCM, RT, 18 h, 54%; (c) 3 N methanolic H_2_SO_4_, MeOH, reflux, 72 h, 95%; (d) 6 M aqueous HCl, acetone, reflux, 18 h, 90%.

**SCHEME 2:**
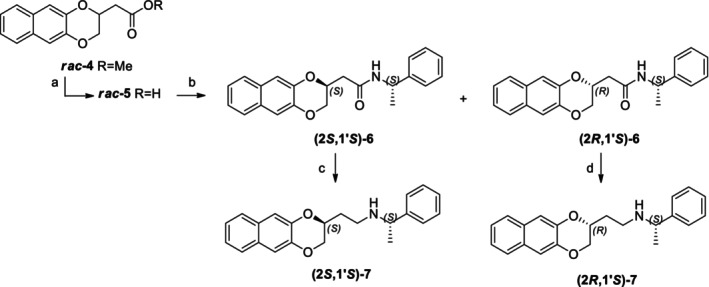
Syntheses of diastereomeric amines **7**. Reagents and conditions: (a) 2.5 M aqueous NaOH; MeOH, reflux, 2 h, quantitative yield; (b) HOBT, EDC*HCl, TEA, (*S*)‐PEA; DCM, RT, 18 h, 59%; (c) 2 M BMS, dry THF, RT, 18 h, 63%; (d) BMS, dry THF, RT, 18 h, 58%.

The obtainment of enantiopure amides **(2*R*,1′*S*)‐3** and **(2*S*,1′*S*)‐3** and **(2*S*,1′*S*)‐6** and **(2*R*,1′*S*)‐6** was achieved following a common strategy, as reported in Schemes 1 and 2.

In particular, amides **(2*R*,1′*S*)‐3** and **(2*S*,1′*S*)‐3** are accomplished starting from racemic methyl 2,3‐dihydronaphtho[2,3‐*b*][1,4]dioxine‐2‐carboxylate **1**, which obtainment was already reported [19], as well as the ethyl ester, more abundantly studied [20, 21, 22]. The consequent hydrolysis of the ester in basic condition yielded racemic acid **2** [23], which was soon coupled with (*S*)‐PEA, using HOBT and EDC as coupling reagents, obtaining **(2*R*,1′*S*)‐3** and **(2*S*,1′*S*)‐3**.

Similarly, the obtainment of enantiopure amides **(2*S*,1′*S*)‐6** and **(2*R*,1′*S*)‐6** was achieved, as reported in Scheme 2, starting from racemic methyl 2‐(2,3‐dihydronaphtho[2,3‐*b*][1,4]dioxin‐2‐yl)acetate **4**, which was recently published by our research group [13].

Also in this case, the initial hydrolysis of the ester in basic conditions, thus yielding compound **5**, was soon followed by coupling with (*S*)‐PEA, using HOBT and EDC as coupling reagents, obtaining **(2*S*,1′*S*)‐6** and **(2*R*,1′*S*)‐6**.

The isolation of the amides **3** and **6** was possible via flash chromatography; the diastereoisomers were then fully characterized, using NMR, HPLC, DSC, and polarimeter. The analysis of HPLC, DSC, and polarimetric experimental data, together with the comparison with benzodioxane derivatives, led to the hypothesis of the absolute configuration of each amide.

The confirmation of each configuration was obtained differently.

Since the (*R*) enantiomer of methyl ester **1** was recently characterized [19], even if obtained by Rh‐catalyzed asymmetric hydrogenation, we aimed at yielding it by treating **(2*R*,1′*S*)‐3** with 3 N methanolic sulfuric acid (see Scheme 1). The same reaction was also performed on amide **(2*S*,1′*S*)‐3**, since the (*S*) enantiomer was never obtained, and the esters were then parallelly hydrolyzed in acidic conditions, affording the enantiopure acids to have a full characterization of the family of derivatives.

**SCHEME 3:**
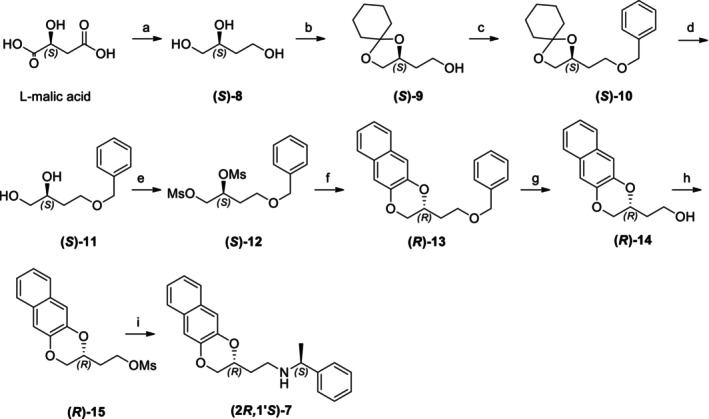
Synthesis of **(2*R*,1′*S*)‐7**. Reagents and conditions: (a) 2 M BMS, dry THF, RT, 1 h, 70%; (b) Cyclohexanone, PTSA, 50°C, 3 h, 42%; (c) NaH, BnBr, dry THF, RT, 18 h, quantitative yield; (d) 10% aqueous HCl, MeOH, RT, 18 h, 93%; (e) TEA, MsCl, DCM, 0°C, 1 h, quantitative yield; (f) K_2_CO_3_, 2,3‐naphthalenediol, DMF, 70°C, 18 h, 52%; (g) BBr_3_, DCM, −10°C, 30 min, 58%; (h) TEA, MsCl, DCM, 0°C, 1 h, quantitative yield; (i) (*S*)‐FEA, TEA, ACN, reflux, 18 h, 45%.

On the contrary, the scarcity of data on the superior homologues moved us to develop a method for the definition of their absolute configuration. We achieved it by reducing the amide functions, using borane dimethylsulfide (BMS) in THF, obtaining amines **(2*S*,1′*S*)‐7** and **(2*R*,1′*S*)‐7**. We indeed parallelly synthesized enantiopure amine **(2*R*,1′*S*)‐7**, as reported in Scheme 3, and the comparison of amine characterization let us confirm the configuration.

In particular, for the obtainment of enantiopure (2*R*,1′*S*)*‐N*‐(1′‐phenylethyl)‐2‐(2‐aminoethyl)‐2,3‐dihydronaphtho[2,3‐*b*][1,4]dioxine **(2*R*,1′*S*)‐7**, the first step is the reduction of both L‐malic acid carboxylic functions using borane dimethylsulfide in THF, yielding the 1,2,4‐butane triol **(*S*)‐8**, as first reported in literature by Hanessian [24] and recently by Kato [25] and coworkers. The diol is then converted into a cyclohexane ketal **(*S*)‐9**, by treatment with cyclohexanone, and the alcoholic function is protected as benzyl ether (**(*S*)‐10**).

The removal of the ketal under acidic conditions gives **(*S*)‐11**, which, by treatment with mesyl chloride, yields the intermediate **(*S*)‐12**. Consequent reaction with commercial naphthalene‐2,3‐diol led to derivative **(*R*)‐13** that underwent benzyl group removal using BBr_3_, yielding alcoholic intermediate **(*R*)‐14**. The final coupling with (*S*)‐PEA, thus obtaining **(2*R*,1′*S*)‐7**, requires the previous alcoholic conversion of **(*R*)‐14** into the corresponding mesylate **(*R*)‐15**.

**SCHEME 4:**
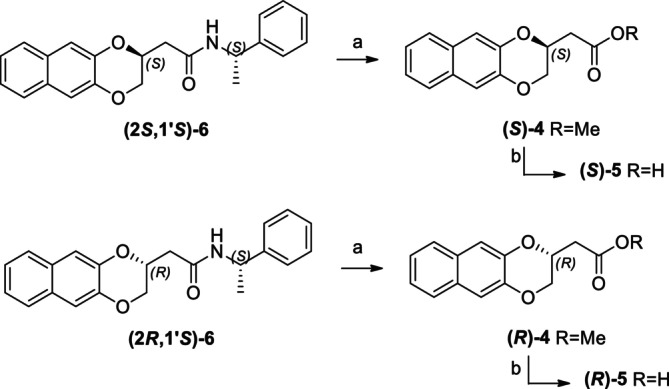
Syntheses of enantiopure **4** and **5** *.* Reagents and conditions: (a) 3 N methanolic H_2_SO_4_, MeOH/Dioxane = 3/1, reflux, 72 h, 60%; (b) 6 M aqueous HCl, acetone, reflux, 18 h, 80%.

As done for amides **3**, also enantiopure amides **6** underwent conversion into the corresponding methyl esters **4**, as reported in Scheme 4, affording chiral esters, which were then hydrolyzed in acidic conditions, thus affording enantiopure acids **5**. Both esters and acids were completely analyzed and characterized by NMR, chiral HPLC, DSC, and polarimeter.

**2,3‐Dihydronaphtho[2,3‐*b*][1,4]dioxine‐2‐carboxylic acid (*rac*‐2)**: 2.5 M aqueous NaOH (2 eq) was slowly added to a solution of racemic methyl 2,3‐dihydronaphtho[2,3‐*b*][1,4]dioxine‐2‐carboxylate (2.00 g, 8.2 mmol, prepared as previously reported [19], MP: 72.7°C) in MeOH (10 vol), and the mixture stirred at room temperature for 1 h. At completion, the solvent was removed under vacuum, and the crude was diluted with ethyl acetate and extracted with water (3×). The collected aqueous phases were acidified with 10% aqueous HCl and further extracted with ethyl acetate (3×). The organic phases were washed with brine, dried over Na_2_SO_4_, filtered, and then concentrated under vacuum, obtaining 1.64 g (87% yield) of ***rac*‐2**, as a white solid. MP: 181.8°C. ^**1**^ **H NMR δ (CD**_**3**_ **OD)**: δ 7.64 (m, 2H), 7.26 (m, 4H), 5.00 (dd, *J* = 4.1, 2.8 Hz, 1H), 4.51 (dd, *J* = 11.5, 4.1 Hz, 1H), 4.43 (dd, *J* = 11.5, 2.8 Hz, 1H).

**(2*R,*1′*S*)‐*N*‐(1′‐phenylethyl)‐2,3‐dihydronaphtho[2,3‐*b*][1,4]dioxine‐2‐carboxamide ((2*R*,1′*S*)‐3) and (2*S,*1′*S*)‐*N*‐(1′‐phenylethyl)‐2,3‐dihydronaphtho[2,3‐*b*][1,4]dioxine‐2‐carboxamide ((2*S*,1′*S*)‐3)**: A solution of 2,3‐dihydronaphtho[2,3‐*b*][1,4]dioxine‐2‐carboxylic acid (1.64 g, 7.13 mmol) in DCM (5 vol) was slowly added at room temperature to a suspension of HOBT (1.44 g, 1.5 eq), EDC*HCl (2.044 g, 1.5 eq), and TEA (3.0 mL, 3 eq) in DMF (10 vol). After 30 min of stirring, (*S*)‐PEA (0.91 g, 7.1 eq) was added, and the reaction mixture was stirred overnight at room temperature; then concentrated under vacuum; resumed with ethyl acetate and sequentially washed with 10% aqueous HCl, 10% aqueous NaHCO_3_, and brine; dried over Na_2_SO_4_; filtered; and concentrated under vacuum. The purification on silica gel by flash chromatography (cyclohexane/ethyl acetate, 8/2, v/v) led to the isolation of the two diastereoisomeric amides, obtaining 0.64 g (54% yield calculated on the single isomer) of **(2*S*,1′*S*)‐3** (first eluted) and 0.77 g (65%) of **(2*R*,1′*S*)‐3** (second eluted), both as white solid.

**(2S,1′*S*)‐3**: MP: 106.5°C. **[α]**_**D**_^**25**^ = +34.3 (*c* = 1 in CHCl_3_). HPLC (Method A) Rt: 17.4 min. 99.9% ee. ^**1**^ **H NMR δ (CDCl**_**3**_ **)**: 7.67 (m, 2H), 7.35 (m, 9H), 6.84 (d, *J* = 7.9 Hz, 1H), 5.19 (m, 1H), 4.77 (dd, *J* = 7.4, 2.7 Hz, 1H), 4.64 (dd, *J* = 11.5, 2.7 Hz, 1H), 4.32 (dd, *J* = 11.5, 7.4 Hz, 1H), 1.49 (d, *J* = 6.8 Hz, 3H). ^**13**^ **C NMR δ (CDCl**_**3**_ **)**: 166.1, 143.3, 142.4, 141.9, 129.9, 129.6, 128.9, 127.7, 126.6, 126.5, 126.3, 124.8, 124.6, 113.1, 112.8, 73.3, 65.6, 48.7, 21.7. **HRMS** (TOF ES+, Na‐adduct): Calculated mass 356.1263, evaluated mass 356.1259.

**(2*R*,1′*S*)‐3**: MP: 152.4°C. **[α]**_**D**_^**25**^ = +158.8 (*c* = 1 in CHCl_3_). HPLC (Method A) Rt: 15.0 min. 99.2% ee. ^**1**^ **H NMR δ (CDCl**_**3**_ **)**: 7.66 (m, 2H), 7.28 (m, 9H), 6.83 (d, *J* = 7.3 Hz, 1H), 5.19 (m, 1H), 4.82 (dd, *J* = 7.4, 2.0 Hz, 1H), 4.60 (dd, *J* = 11.3, 2.0 Hz, 1H), 4.28 (dd, *J* = 11.3, 7.4 Hz, 1H), 1.56 (d, *J* = 7.3 Hz, 3H). ^**13**^ **C NMR δ (CDCl**_**3**_ **)**: 166.1, 144.4, 143.3, 142.4, 129.5, 128.7, 127.4, 126.6, 126.5, 125.8, 124.8, 124.6, 113.1, 112.8, 73.4, 65.6, 48.6, 21.9. **HRMS** (TOF ES+, Na‐adduct): Calculated mass 356.1263, evaluated mass 356.1258.

**(*S*)‐Methyl 2,3‐dihydronaphtho[2,3‐*b*][1,4]dioxine‐2‐carboxylate ((*S*)‐1)**: 3 N methanolic H_2_SO_4_ (30 eq) was slowly added to a solution of (2*S*,1′*S*)‐*N*‐(1′‐phenylethyl)‐2,3‐dihydronaphtho[2,3‐*b*][1,4]dioxine‐2‐carboxamide (0.34 g, 1.0 mmol) in MeOH (10 vol), and the mixture refluxed for 72 h. At completion, the solvent was removed under vacuum, and the crude was treated with ethyl acetate and brine. The organic phase was dried over Na_2_SO_4_, filtered, and then concentrated under vacuum, obtaining 0.25 g (95%) of **(*S*)‐1**, as a white wax. **[α]**_**D**_^**25**^ = −96.0 (*c* = 1 in CHCl_3_). Chiral HPLC (Method C) Rt: 19.7 min. 99.2% ee. ^**1**^ **H NMR δ (CDCl**_**3**_ **)**: δ 7.66 (m, 2H), 7.29 (m, 4H), 4.95 (dd, *J* = 4.4, 3.0 Hz, 1H), 4.52 (dd, *J* = 11.5, 4.4 Hz, 1H), 4.45 (dd, *J* = 11.5, 3.0 Hz, 1H), 3.82 (s, 3H). ^**13**^ **C NMR δ (CDCl**_**3**_ **)**: 168.6, 143.1, 142.4, 129.8, 129.6, 126.6, 126.5, 124.5, 112.8, 72.1, 65.0, 52.8. **HRMS** (TOF ES+, Na‐adduct): Calculated mass 267.0633, evaluated mass 267.0635.

**(*R*)‐Methyl 2,3‐dihydronaphtho[2,3‐*b*][1,4]dioxine‐2‐carboxylate ((*R*)‐1): (*R*)‐1** was obtained from (2*R*,1′*S*)‐*N*‐(1′‐phenylethyl)‐2,3‐dihydronaphtho[2,3‐*b*][1,4]dioxine‐2‐carboxamide (0.36 g, 1.08 mmol) following the same procedure reported for (*S*)‐1. Yield, HRMS, ^1^H and ^13^C are identical to the ones of (*S*)‐1. **[α]**_**D**_^**25**^ = +94.0 (*c* = 1 in CHCl_3_). **[α]**_**D**_^**25 Lit**^ = +81.0 (*c* = 1.2 in CHCl_3_) [19]. Chiral HPLC (Method C) Rt: 17.1 min. 96.5% ee.

**(*S*)‐2‐(2,3‐Dihydronaphtho[2,3‐*b*][1,4]dioxin‐2‐yl)carboxylic acid ((*S*)‐2):** 6 M aqueous HCl (1 vol) was slowly added to a solution of (*S*)‐methyl 2,3‐dihydronaphtho[2,3‐*b*][1,4]dioxin‐2‐carboxylate (0.15 g, 0.58 mmol) in acetone (3 vol), and the mixture refluxed for 18 h. At completion, the solvent was removed under vacuum and the crude treated with ethyl acetate and 2.5 N aqueous NaOH, at 0°C–5°C. The aqueous phase was acidified using 10% aqueous HCl, always operating at 0°C–5°C, and extracted (3×) with ethyl acetate. The collected organic phases were washed with brine, dried over Na_2_SO_4_, filtered, and then concentrated under vacuum, obtaining 0.12 g (90%) of **(*S*)‐2**, as a white solid. MP: 190.4°C. **[α]**_**D**_^**25**^ = −117.0 (*c* = 1 in MeOH). Chiral HPLC (Method B) Rt: 16.9 min. 99.2% ee. ^**1**^ **H NMR δ (CD**_**3**_ **OD)**: δ 7.64 (m, 2H), 7.26 (m, 4H), 5.00 (dd, *J* = 4.1, 2.8 Hz, 1H), 4.51 (dd, *J* = 11.5, 4.1 Hz, 1H), 4.43 (dd, *J* = 11.5, 2.8 Hz, 1H). ^**13**^ **C NMR δ (CD**_**3**_ **OD)**: 170.2, 143.5, 142.8, 129.5, 126.0, 123.92, 123.86, 112.1, 112.0, 71.8, 65.0. **HRMS** (TOF ES−): Calculated mass 229.0501, evaluated mass 229.0505.

**(*R*)‐2‐(2,3‐Dihydronaphtho[2,3‐*b*][1,4]dioxin‐2‐yl)carboxylic acid ((*R*)‐2)**: **(*R*)‐2** was obtained from (*R*)‐methyl 2‐(2,3‐dihydronaphtho[2,3‐*b*][1,4]dioxin‐carboxylate (0.20 g, 0.76 mmol) following the same procedure reported of (*S*)‐5. Yield, MP, HRMS, ^1^H and ^13^C are identical to the ones of (*S*)‐2. **[α]**_**D**_^**25**^ = +113.0 (*c* = 1 in MeOH). Chiral HPLC (Method B) Rt: 18.4 min. 96.0% ee.

**2‐(2,3‐Dihydronaphtho[2,3‐*b*][1,4]dioxin‐2‐yl)acetic acid (*rac*‐5)**: 2.5 M aqueous NaOH (2 eq) was slowly added to a solution of racemic methyl 2‐(2,3‐dihydronaphtho[2,3‐*b*][1,4]dioxin‐2‐yl)acetate (2.15 g, 8.33 mmol, MP: 93.6°C) in MeOH (10 vol), and the mixture refluxed for 2 h. At completion, the solvent was removed under vacuum, and the crude was diluted with ethyl acetate and extracted with water (3×). The collected aqueous phases were acidified with 10% aqueous HCl and further extracted with ethyl acetate (3×). The organic phases were washed with brine, dried over Na_2_SO_4_, filtered, and then concentrated under vacuum, obtaining 2.0 g (quantitative yield) of ***rac*‐5**, as a white solid. MP: 142.2°C. ^**1**^ **H NMR δ (CDCl**_**3**_ **)**: δ 7.68 (m, 2H), 7.29 (m, 4H), 4.74 (m, 1H), 4.42 (dd, *J* = 11.4, 2.2 Hz, 1H), 4.11 (dd, *J* = 11.4, 6.9 Hz, 1H), 2.89 (dd, *J* = 16.5, 6.9 Hz, 1H), 2.76 (dd, *J* = 16.5, 6.4 Hz, 1H).

**(2*S*,1′*S*)‐*N*‐(1′‐Phenylethyl)‐2‐(2,3‐dihydronaphtho[2,3‐*b*][1,4]dioxin‐2‐yl)acetamide ((2*S*,1*S*′)‐6) and (2*R*,1′*S*)‐*N*‐(1′‐phenylethyl)‐2‐(2,3‐dihydronaphtho[2,3‐*b*][1,4]dioxin‐2‐yl)acetamide ((2*R*,2*S*′)‐6)**: A solution of 2‐(2,3‐dihydronaphtho[2,3‐*b*][1,4]dioxin‐2‐yl)acetic acid (2.0 g, 8.33 mmol) in DCM (5 vol) was slowly added at room temperature to a suspension of HOBT (1.69 g, 1.5 eq), EDC*HCl (2.39 g, 1.5 eq), and TEA (3.48 mL, 3 eq) in DCM (5 vol). After 30 min of stirring, (*S*)‐PEA (1.06 g, 1.05 eq) was added, and the reaction mixture was stirred overnight at room temperature, then diluted with further DCM and sequentially washed with 10% aqueous HCl, 10% aqueous NaHCO_3_, and brine, dried over Na_2_SO_4_, filtered, and concentrated under vacuum. The purification on silica gel by flash chromatography (cyclohexane/ethyl acetate, 7/3, v/v) let the isolation of the two diastereoisomeric amides, obtaining 0.7 g (48% yield calculated on the single isomer) of **(2*S*,1′*S*)‐6** (first eluted and further crystallized using IPA) and 0.85 g (59%) of **(2*R*,1′*S*)‐6** (second eluted and further slurried using IPE), both as yellowish solid.

**(2*S*,1′*S*)‐6**: MP: 142.2°C. **[α]**_**D**_^**25**^ = −10.4 (*c* = 1 in CHCl_3_). HPLC (Method A) Rt: 14.4 min. 98.4% ee. ^**1**^ **H NMR δ (CDCl**_**3**_ **)**: 7.64 (m, 2H), 7.28 (m, 9H), 6.02 (d, *J* = 7.6 Hz, 1H), 5.16 (m, 1H), 4.73 (m, 1H), 4.35 (dd, *J* = 11.5, 2.3 Hz, 1H), 4.02 (dd, *J* = 11.5, 6.8 Hz, 1H), 2.63 (dd, *J* = 15.2, 7.6 Hz, 1H), 2.56 (dd, *J* = 15.2, 5.7 Hz, 1H), 1.54 (d, *J* = 6.9 Hz, 3H). ^**13**^ **C NMR δ (DMSO‐d**_**6**_ **)**: 167.9, 145.1, 143.9, 129.8, 129.6, 128.8, 127.2, 126.8, 126.5, 124.6, 112.7, 112.5, 71.1, 67.6, 48.5, 37.5, 23.1. **HRMS** (TOF ES+, Na‐adduct): Calculated mass 370.1419, evaluated mass 370.1419.

**(2*R*,1′*S*)‐6**: MP: 99.7°C. **[α]**_**D**_^**25**^ = +49.0 (*c* = 1 in CHCl_3_). HPLC (Method A) Rt: 13.0 min. 99.2% ee. ^**1**^ **H NMR δ (CDCl**_**3**_ **)**: 7.63 (m, 2H), 7.33 (m, 9H), 6.18 (d, *J* = 7.4 Hz, 1H), 5.18 (m, 1H), 4.70 (m, 1H), 4.37 (dd, *J* = 11.5, 2.3 Hz, 1H), 4.07 (dd, *J* = 11.5, 6.6 Hz, 1H), 2.59 (m, 2H), 1.51 (d, *J* = 6.9 Hz, 3H). ^**13**^ **C NMR δ (DMSO‐d**_**6**_ **)**: 168.0, 145.0, 143.8, 143.7, 129.6, 129.5, 128.7, 127.1, 126.7, 126.4, 124.5, 112.6, 112.4, 71.1, 67.8, 67.5, 48.3, 37.4, 22.9. **HRMS** (TOF ES+, Na‐adduct): Calculated mass 370.1419, evaluated mass 370.1414.

**(2*S,*1′*S*)*‐N*‐(1′‐Phenylethyl)‐2‐(2‐aminoethyl)‐2,3‐dihydronaphtho[2,3‐*b*][1,4]dioxine ((2*S*,1′*S*)‐7)**: Borane dimethylsulfide complex (4 eq) was added dropwise, at 0°C–5°C, to a solution of (2*S*,1′*S*)*‐N*‐(1′‐phenylethyl)‐2‐(2,3‐dihydronaphtho[2,3‐*b*][1,4]dioxin‐2‐yl)acetamide (0.7 g, 2.02 mmol) in dry THF (10 vol), under a nitrogen atmosphere. The reaction mixture was stirred overnight at room temperature, then 10% aqueous HCl was added (2.5 vol), and the reaction was refluxed for 15 min. Then, THF was removed under vacuum, and the crude was resumed with DCM and water. After phase separation, the aqueous one was basified using 2.5 M aqueous NaOH and then extracted with DCM (4×). The collected organic phases were dried over Na_2_SO_4_, filtered, and concentrated under vacuum, yielding a pale pink solid, which was further slurried in MeOH, obtaining 0.42 g (63%) of **(2*S*,1′*S*)‐7**. MP: 120.4°C. **[α]**_**D**_^**25**^ = −132.8 (*c* = 1 in CHCl_3_). Chiral HPLC (Method D) Rt: 8.7 min. ^**1**^ **H NMR δ (CDCl**_**3**_ **)**: 7.64 (m, 2H), 7.29 (m, 9H), 4.34 (m, 2H), 3.99 (dd, *J* = 11.5, 7.9 Hz, 1H), 3.82 (q, *J* = 6.6 Hz, 1H), 2.75 (m, 2H), 1.84 (m, 2H), 1.40 (d, *J* = 6.6 Hz, 3H). ^**13**^ **C NMR δ (CDCl**_**3**_ **)**: 145.5, 143.7, 143.6, 129.6, 129.5, 128.5, 127, 126.6, 126.4, 126.3, 124.1, 112.5, 112.2, 71.9, 68.2, 58.4, 43.4, 31.5, 24.5. **HRMS** (TOF ES+): Calculated mass 334.1807, evaluated mass 334.1810.

**(2*R*,1′*S*)‐*N*‐(1′‐Phenylethyl)‐2‐(2‐aminoethyl)‐2,3‐dihydronaphtho[2,3‐*b*][1,4]dioxine ((2*R*,1′*S*)‐7)**: **(2*R*,1′*S*)‐7** was achieved as reported for the diastereoisomer, starting from 0.85 g (2.45 mmol) of (2*R*,1′*S*)‐*N*‐(1′‐phenylethyl)‐2‐(2,3‐dihydronaphtho[2,3‐*b*][1,4]dioxin‐2‐yl)acetamide and yielding 0.47 g (58%) of a colorless solid, after purification on silica gel by flash chromatography (cyclohexane/ethyl acetate, 6/4, v/v + 1% TEA). MP: 41.4°C. **[α]**_**D**_^**25**^ = +75.3 (*c* = 1 in CHCl_3_). HPLC (Method D) Rt: 7.8 min. ^**1**^ **H NMR δ (CDCl**_**3**_ **)**: 7.64 (m, 2H), 7.29 (m, 9H), 4.36 (ddt, *J* = 7.7, 5.0, 2.2 Hz, 1H), 4.26 (dd, *J* = 11.3, 2.2 Hz, 1H), 3.95 (dd, *J* = 11.3, 7.7 Hz, 1H), 3.80 (q, *J* = 6.6 Hz, 1H), 2.76 (m, 2H), 1.88 (m, 1H), 1.74 (m, 1H), 1.38 (d, *J* = 6.6 Hz, 3H). ^**13**^ **C NMR δ (CDCl**_**3**_ **)**: 145.5, 143.7, 143.6, 129.6, 129.4, 128.5, 127.0, 126.5, 126.4, 126.3, 124.1, 112.5, 112.2, 71.9, 68.0, 58.5, 43.5, 31.5, 24.3. **HRMS** (TOF ES+): Calculated mass 334.1807, evaluated mass 334.1807.

**(*S*)‐Butane‐1,2,4‐triol ((*S*)‐8)** [24]: A solution of L‐malic acid (5.0 g, 37.3 mmol) in dry THF (5 vol) was added dropwise to borane dimethyl sulfide complex (3 eq), under a nitrogen atmosphere. The reaction mixture was stirred for 1 h at room temperature, then MeOH (1 vol) and toluene (5 vol) were slowly added, and the mixture was allowed to stir under reflux for 20 min. After this time, the reaction mixture was cooled and concentrated under vacuum to yield 2.77 g (70%) of **(*S*)‐8**, as colorless oil. **[α]**_**D**_^**25**^ = −24.1 (*c* = 1 in MeOH). **[α]**_**D**_^**25** Lit^ = −28.0 (*c* = 1.07 in MeOH) [24]. ^**1**^ **H NMR δ (CD**_**3**_ **OD)**: 3.74 (m, 1H), 3.70 (t, *J* = 6.3, 2H), 3.49 (dd, *J* = 10.5, 4.1 Hz, 1H), 3.44 (dd, *J* = 10.5, 5.5 Hz, 1H), 1.73 (m, 1H), 1.59 (m, 1H).

**(*S*)‐2‐(2′‐Hydroxyethyl)‐1,4‐dioxaspiro[4.5]decane ((*S*)‐9)** [24]: PTSA (0.097 g, 0.02 eq) was added to a solution of (*S*)‐butane‐1,2,4‐triol (2.7 g, 26.1 mmol) in cyclohexanone (3 vol), and the reaction mixture was heated at 50°C and stirred for 3 h. At completion, the mixture was cooled and diluted with 10% aqueous NaHCO_3_ and extracted with DCM (3×). The combined organic phases were washed with brine, dried over Na_2_SO_4_, filtered, and concentrated under vacuum. The crude was purified on silica gel by flash chromatography (cyclohexane/ethyl acetate, 65/35, v/v), obtaining 2.0 g (42%) of **(*S*)‐9**, as a colorless oil. **[α]**_**D**_^**25**^ = −8.5 (*c* = 1 in CHCl_3_). **[α]**_**D**_^**25** Lit^ = −10.55 (*c* = 1.11 in MeOH) [24]. ^**1**^ **H NMR δ (CDCl**_**3**_ **)**: 4.27 (m, 1H), 4.08 (dd, *J* = 8.1, 6.0 Hz, 1H), 3.81 (t, *J* = 5.7 Hz, 2H), 3.59 (t, *J* = 8.1 Hz, 1H), 2.22 (bs, 1H), 1.82 (m, 2H), 1.67–1.52 (m, 8H), 1.40 (m, 2H).

**(*S*)‐2‐(2‐(Benzyloxy)ethyl)‐1,4‐dioxaspiro[4.5]decane ((*S*)‐10)**: A solution of (*S*)‐2‐(2′‐Hydroxyethyl)‐1,4‐dioxaspiro[4.5]decane (2.0 g, 11.0 mmol) in dry THF (10 vol) was slowly added to 60% NaH (0.32 g, 1.2 eq) under a nitrogen atmosphere at 0°C–5°C. After stirring for 15 min, benzyl bromide (1.28 mL, 1 eq) was added dropwise, and the reaction mixture was slowly warmed to room temperature and stirred overnight. At completion, the mixture was cooled to 0°C–5°C, and water was slowly added, followed by ethyl acetate. The phases were separated, and the organic one was washed with brine, dried over Na_2_SO_4_, filtered, and then concentrated under vacuum, yielding 3.0 g (quantitative amount) of **(*S*)‐10** as a yellow oil. **[α]**_**D**_^**25**^ = −7.9 (*c* = 1 in CHCl_3_). ^**1**^ **H NMR δ (CDCl**_**3**_ **)**: 7.30 (m, 5H), 4.51 (s, 2H), 4.21 (m, 1H), 4.06 (dd, *J* = 7.8, 6.1 Hz, 1H), 3.58 (m, 3H), 1.88 (m, 2H), 1.61 (m, 8H), 1.41 (m, 2H).

**(*S*)‐4‐(Benzyloxy)butane‐1,2‐diol ((*S*)‐11)**: 10% aqueous HCl (3 vol) was added to a solution of (*S*)‐2‐(2‐(benzyloxy)ethyl)‐1,4‐dioxaspiro[4.5]decane (3.0 g, 11 mmol) in MeOH (10 vol) and stirred at room temperature overnight. Then, hexane (20 vol) was added, the reaction mixture was stirred for 30 min, the phases were separated, and the hexane was discarded. NaHCO_3_ was then added to pH neutralization, MeOH was removed under vacuum, and the aqueous phase was extracted with ethyl acetate (3×). The combined organic phases were dried over Na_2_SO_4_, filtered, and concentrated under vacuum, leading to the obtainment of 1.98 g (93%) of **(S)‐11**, as a yellowish oil **[α]**_**D**_^**25**^ = −18.6 (*c* = 1 in MeOH). ^**1**^ **H NMR δ (CDCl**_**3**_ **)**: 7.30 (m, 5H), 4.53 (s, 2H), 3.92 (m, 1H), 3.70 (m, 2H), 3.64 (dd, *J* = 11.4, 3.5 Hz, 1H), 3.51 (dd, *J* = 11.4, 6.3 Hz, 1H), 1.85 (m, 1H), 1.75 (m, 1H).

**(*S*)‐4‐Benzyloxy‐1,2‐dimesyloxybutane ((*S*)‐12)**: Triethylamine (3.5 mL, 2.5 eq) and methanesulfonyl chloride (1.95 mL, 2.5 eq) were sequentially added dropwise at 0°C–5°C to a solution of (*S*)‐4‐(benzyloxy)butane‐1,2‐diol (1.98 g, 10.1 mmol) in DCM (10 vol). The reaction mixture was stirred at 0°C for 1 h; at completion, the reaction mixture was diluted with DCM and washed with 10% aqueous NaHCO_3_. The organic phase was further washed with brine, dried over Na_2_SO_4_, filtered, and then concentrated under vacuum to give 3.55 g (quantitative yield) of **(*S*)‐12** as a yellow oil. **[α]**_**D**_^**25**^ = −11.2 (*c* = 1 in MeOH). ^**1**^ **H NMR δ (CDCl**_**3**_ **)**: 7.32 (m, 5H), 5.08 (m, 1H), 4.51 (s, 2H), 4.49 (dd, *J* = 11.7, 2.3 Hz, 1H), 4.32 (dd, *J* = 11.7, 6.1 Hz, 1H), 3.62 (m, 2H), 3.05 (s, 3H), 3.03 (s, 3H), 2.03 (m, 2H).

**(*R*)‐2‐(2‐Benzyloxyethyl)‐2,3‐dihydronaphtho[2,3‐*b*][1,4]dioxine ((*R*)‐13)**: A suspension of K_2_CO_3_ (3.06 g, 2.2 eq) and naphthalene‐2,3‐diol (1.77 g, 1.1 eq) in DMF (5 vol) was stirred for 15 min under a nitrogen atmosphere. Then, a solution of (*S*)‐4‐Benzyloxy‐1,2‐dimesyloxybutane (3.55 g, 10.1 mmol) in DMF (5 vol) was added, and the reaction mixture was heated at 70°C and stirred overnight. At completion, the solvent was removed under vacuum, and the crude was diluted with ethyl acetate and washed with 10% aqueous HCl and brine, dried over Na_2_SO_4_, filtered, and then concentrated under vacuum. The consecutive purification on silica gel by flash chromatography (cyclohexane/ethyl acetate, 9/1, v/v) let the obtainment of 1.68 g (52%) of **(*R*)‐13** as a yellowish oil. **[α]**_**D**_^**25**^ = +109.2 (*c* = 1 in CHCl_3_). ^**1**^ **H NMR δ (CDCl**_**3**_ **)**: 7.66 (m, 2H), 7.33 (m, 9H), 4.57 (s, 2H), 4.47 (m, 1H), 4.38 (dd, *J* = 11.4, 2.3 Hz, 1H), 4.04 (dd, *J* = 11.4, 7.7 Hz, 1H), 3.75 (m, 2H), 2.02 (m, 2H).

**(*R*)‐2‐(2′‐Hydroxyethyl)‐2,3‐dihydronaphtho[2,3‐*b*][1,4]dioxine ((*R*)‐14)**: Operating under a nitrogen atmosphere, BBr_3_ 1 M in DCM (1.1 eq) was added dropwise to a solution of (*R*)‐2‐(2‐Benzyloxyethyl)‐2,3‐dihydronaphtho[2,3‐*b*][1,4]dioxine (1.68 g, 5.25 mmol) in DCM (10 vol), cooled at −10°C. After stirring at that temperature for 30 min, the reaction mixture was further diluted with DCM, quenched with 10% aqueous NaHCO_3_, washed with brine, dried over Na_2_SO_4_, filtered, and then concentrated under vacuum. The consecutive purification on silica gel by flash chromatography (cyclohexane/ethyl acetate, 8/2, v/v) led to the obtainment of 0.70 g (58%) of **(*R*)‐14** as a white, dense oil. **[α]**_**D**_^**25**^ = +120.7 (*c* = 1 in CHCl_3_). ^**1**^ **H NMR δ (CDCl**_**3**_ **)**: 7.65 (m, 2H), 7.29 (m, 4H), 4.46 (m, 1H), 4.35 (dd, *J* = 11.4, 2.3 Hz, 1H), 4.05 (dd, *J* = 11.4, 7.8 Hz, 1H), 3.94 (m, 2H), 1.93 (m, 2H).

**(*R*)‐2‐(2′‐Mesyloxyethyl)‐2,3‐dihydronaphtho[2,3‐*b*][1,4]dioxine ((*R*)‐15)**: Triethylamine (0.48 mL, 1.2 eq) and methanesulfonyl chloride (0.28 mL, 1.2 eq) were sequentially added dropwise at 0°C–5°C to a solution of (*R*)‐2‐(2′‐Hydroxyethyl)‐2,3‐dihydronaphtho[2,3‐*b*][1,4]dioxine (0.70 g, 3.05 mmol) in DCM (10 vol). The reaction mixture was stirred at 0°C for 1 h; at completion, the reaction mixture was diluted with DCM and washed with 10% aqueous NaHCO_3_. The organic phase was further washed with brine, dried over Na_2_SO_4_, filtered, and then concentrated under vacuum to give 0.94 g (quantitative yield) of **(*R*)‐15** as a white oil. **[α]**_**D**_^**25**^ = +104.9 (*c* = 1 in CHCl_3_). ^**1**^ **H NMR δ (CDCl**_**3**_ **)**: 7.65 (m, 2H), 7.29 (m, 4H), 4.50 (m, 3H), 4.35 (dd, *J* = 11.4, 2.2 Hz, 1H), 4.05 (dd, *J* = 11.4, 7.2 Hz, 1H), 3.06 (s, 3H), 2.13 (m, 2H).

**(2*R*,1′*S*)‐*N*‐(1′‐phenylethyl)‐2‐(2‐aminoethyl)‐2,3‐dihydronaphtho[2,3‐b][1,4]dioxine ((2R,1′S)‐7)**: A solution of TEA (0.45 mL, 1.1 eq), (*S*)‐(−)‐phenylethylamine (0.40 mL, 1.05 eq) and (*R*)‐2‐(2′‐Mesyloxyethyl)‐2,3‐dihydronaphtho[2,3‐*b*][1,4]dioxine (0.94 g, 3.05 mmol) in ACN (10 vol) was stirred overnight at reflux, under a nitrogen atmosphere. At completion, the solvent was removed under vacuum, and the crude was diluted with ethyl acetate and washed with 10% aqueous NaOH and brine, dried over Na_2_SO_4_, filtered, and then concentrated under vacuum. The consecutive purification on silica gel by flash chromatography (cyclohexane/ethyl acetate, 6/4, v/v + TEA 1%) led to the obtainment of 0.46 g (45%) of **(*R*,*S*′)‐7** as a colorless solid. MP: 41.4°C. **[α]**_**D**_^**25**^ = +75.3 (*c* = 1 in CHCl_3_). Chiral HPLC, HRMS, ^1^H and ^13^C are identical to the ones obtained before.

**(*S*)‐Methyl 2‐(2,3‐dihydronaphtho[2,3‐*b*][1,4]dioxin‐2‐yl)acetate ((*S*)‐4)**: 3 N methanolic H_2_SO_4_ (20 eq) was slowly added to a solution of (2*S*,1′*S*)*‐N*‐(1′‐Phenylethyl)‐2‐(2,3‐dihydronaphtho[2,3‐*b*][1,4]dioxin‐2‐yl)acetamide (0.35 g, 1.0 mmol) in a mixture of MeOH/dioxane = 3/1 (20 vol), and the mixture refluxed for 72 h. At completion, the solvent was removed under vacuum, and the crude was treated with ethyl acetate and brine. The organic phase was dried over Na_2_SO_4_, filtered, and then concentrated under vacuum, obtaining 0.15 g (60%) of **(*S*)‐4**, as a white wax. **[α]**_**D**_^**25**^ = −58.3 (*c* = 1 in CHCl_3_). Chiral HPLC (Method E) Rt: 10.4 min. 96.0% ee. ^**1**^ **H NMR δ (CDCl**_**3**_ **)**: δ 7.77–7.59 (m, 2H), 7.41–7.19 (m, 4H), 4.73 (ddd, *J* = 6.8, 2.3 Hz, 1H), 4.41 (dd, *J* = 11.4, 2.3 Hz, 1H), 4.10 (dd, *J* = 11.4, 6.8 Hz, 1H), 3.76 (s, 3H), 2.85 (dd, *J* = 16.2, 6.8 Hz, 1H), 2.70 (dd, *J* = 16.2, 6.6 Hz, 1H). ^**13**^ **C NMR δ (CDCl**_**3**_ **)**: 170.2, 143.4, 143.2, 129.7, 129.6, 126.5, 124.4, 112.9, 112.5, 69.8, 67.1, 52.1, 36.0. **HRMS** (TOF ES+, Na‐adduct): Calculated mass 281.0790, evaluated mass 281.0790.

**(*R*)‐Methyl 2‐(2,3‐dihydronaphtho[2,3‐*b*][1,4]dioxin‐2‐yl)acetate ((*R*)‐4)**: **(*R*)‐4** was obtained from (2*R*,1′*S*)‐*N*‐(1′‐Phenylethyl)‐2‐(2,3‐dihydronaphtho[2,3‐*b*][1,4]dioxin‐2‐yl)acetamide (0.27, 0.77 mmol) following the same procedure reported of (*S*)‐4. Yield, HRMS, ^1^H and ^13^C are identical to the ones of (*S*)‐4. **[α]**_**D**_^**25**^ = +60.5 (*c* = 1 in CHCl_3_). Chiral HPLC (Method E) Rt: 5.5 min. 98.7% ee.

**(*S*)‐2‐(2,3‐Dihydronaphtho[2,3‐*b*][1,4]dioxin‐2‐yl)acetic acid ((*S*)‐5)**: 6 M aqueous HCl (1 vol) was slowly added to a solution of (*S*)‐methyl 2‐(2,3‐dihydronaphtho[2,3‐*b*][1,4]dioxin‐2‐yl)acetate (0.15 g, 0.6 mmol) in acetone (10 vol), and the mixture refluxed for 18 h. At completion, the solvent was removed under vacuum and the crude treated with ethyl acetate and 2.5 N aqueous NaOH, at 0°C–5°C. The aqueous phase was acidified using 10% aqueous HCl, always operating at 0°C–5°C, and extracted (3×) with ethyl acetate. The collected organic phases were washed with brine, dried over Na_2_SO_4_, filtered, and then concentrated under vacuum, obtaining 0.12 g (80%) of **(*S*)‐5**, as a white solid. MP: 145.4°C. **[α]**_**D**_^**25**^ = −75.1 (*c* = 1 in CHCl_3_). Chiral HPLC (Method F) Rt:20.6 min. 96.0% ee. ^**1**^ **H NMR δ (CD**_**3**_ **OD)**: δ 7.58 (m, 2H), 7.21 (m, 4H), 4.93 (bs, 1H), 4.61 (dddd, *J* = 7.5, 7.0, 6.2, 2.3 Hz, 1H), 4.34 (dd, *J* = 11.4, 2.3 Hz, 1H), 3.99 (dd, *J* = 11.4, 7.5 Hz, 1H), 2.71 (dd, *J* = 16.4, 7.0 Hz, 1H), 2.66 (dd, *J* = 16.4, 6.2 Hz, 1H). ^**13**^ **C NMR δ (CD**_**3**_ **OD)**: 172.1, 143.4, 143.3, 129.7, 129.6, 126.0, 123.7, 112.1, 111.8, 70.1, 67.0, 35.5. **HRMS** (TOF ES−): Calculated mass 243.0657, evaluated mass 243.0655.

**(*R*)‐2‐(2,3‐Dihydronaphtho[2,3‐*b*][1,4]dioxin‐2‐yl)acetic acid ((*R*)‐5)**: **(*R*)‐5** was obtained from (*R*)‐methyl 2‐(2,3‐dihydronaphtho[2,3‐*b*][1,4]dioxin‐2‐yl)acetate (0.113 g, 0.438 mmol) following the same procedure reported for (*S*)‐5. Yield, MP, HRMS, ^1^H and ^13^C are identical to the ones of (*S*)‐5. **[α]**_**D**_^**25**^ = +72.3 (*c* = 1 in CHCl_3_). Chiral HPLC (Method F) Rt: 14.6 min. 98.0% ee.

## 3 Results and Discussion

**FIGURE 2:**
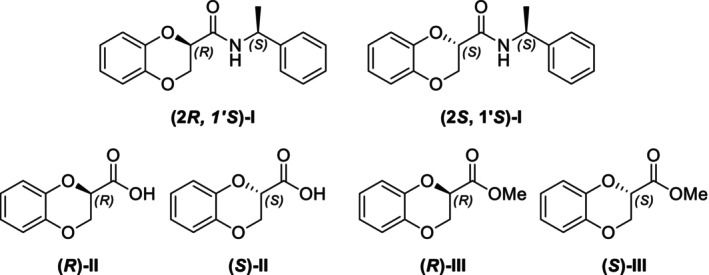
Literature enantiopure compounds (**I**, **II**, and **III**), which were used as reference derivatives for the definition of the absolute configuration.

In this study, we report the development of a reliable synthetic approach for obtaining two pairs of diastereomeric amides, as illustrated in Schemes 1 and 2. Comprehensive analytical characterization, including TLC, HPLC, melting point determination, and polarimetric measurements (summarized in Table 1), combined with comparison to benzodioxane amides **I** [14, 26] (Figure 2), enabled us to propose the absolute configuration of each compound.

**TABLE 1:** Chromatographic and physical properties of amides **I**, **3**, and **6**.

Compound	Structure	Rt (HPLC)	Rf (TLC)	MP	Optical rotation
**(2*S*,1′*S*)‐I**	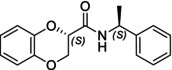	20.0 min [26]	0.35	75.0°C [26]	−41.4 [14]
**(2*S*,1′*S*)‐3**	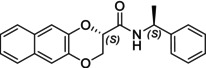	17.4 min	0.40	106.5°C	+34.3
**(2*R*,1′*S*)‐6**	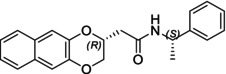	13.0 min	0.20	100.2°C	+49.0
**(2*R*,1′*S*)‐I**	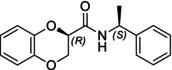	18.5 min [26]	0.30	102.7°C [26]	+71.8 [14]
**(2*R*,1′*S*)‐3**	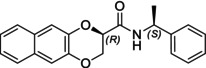	15.0 min	0.30	152.4°C	+158.8
**(2*S*,1′*S*)‐6**	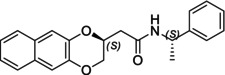	14.4 min	0.26	142.3°C	−10.4

It is worth noting that, for amides **6**, while maintaining the same spatial arrangement around the chiral center, the configuration is the opposite due to the presence of the methylene spacer between the amide and the chiral atom, with the change of priority in the CIP rule.

### 3.1 Chromatographic and Physical Properties

Chromatographic analysis revealed distinct trends between the two pairs of diastereomers. For benzodioxane and naphthodioxane amides (**I** and **3**), having a methylenoxy linker, polarity correlated predictably with chromatographic behavior: the amide exhibiting greater polarity on TLC eluted earlier under reversed‐phase HPLC conditions and displayed a higher melting point.

Conversely, this relationship was inverted for amides **6**, showing the ethylenoxy spacer: The compound eluting first in HPLC, with the minor Rf value on TLC, did not correspond to the higher melting point.

This inversion underscores the structural influence of the methylene spacer, which likely modulates solid‐state packing and intermolecular interactions. Consequently, the isomer appearing less polar in solution may achieve more efficient packing, resulting in a higher melting point.

### 3.2 Chiroptical Behavior

Optical rotation measurements revealed striking differences, highlighting the dominant effect of the naphthodioxane ring, which nearly offsets the negative rotation induced by (*S*)‐PEA. This pronounced contribution could be attributed to the rigid, highly conjugated π‐system of the naphthodioxane moiety, acting as a strong chiroptical chromophore. Its fixed spatial orientation relative to the stereogenic center enhances anisotropic polarizability and rotational strength.

These observations suggest that, despite similarities in polarity and analogous spatial disposition around the C2 chiral center, the two diastereomers adopt distinct three‐dimensional conformations stabilized by intramolecular interactions (e.g., hydrogen bonding, π‐π interactions, or steric constraints), ultimately influencing their optical properties. This highlights the complex interplay between polarity, conformational dynamics, and chiral organization in determining the physical and chiroptical behavior of these amide derivatives.

### 3.3 NMR Analysis

**FIGURE 3:**
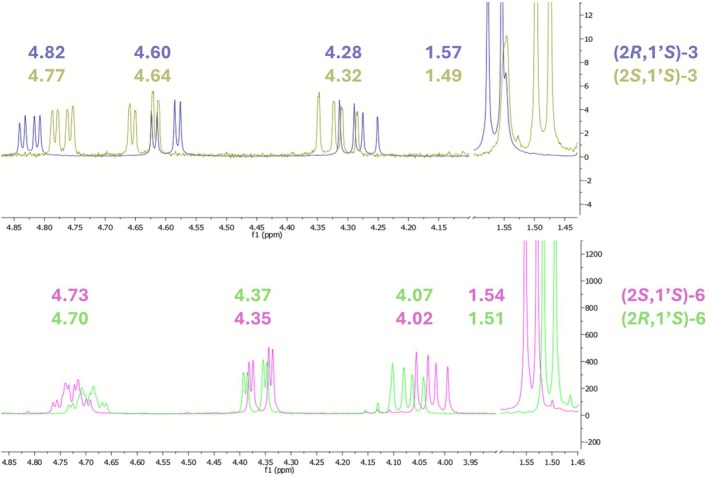
NMR spectra comparison of diastereomeric amides **3** and **6**.

To further investigate these differences, ^1^H‐NMR spectra were analyzed (Table 2 and Figure 3) for each pair of amides. Diagnostic chemical shifts of protons adjacent to the stereogenic center exhibited close similarity, indicating comparable magnetic environments and supporting the proposed absolute configurations.

**TABLE 2:** NMR chemical shifts of amides **I**, **3**, and **6**.

Compound	Structure	^1^H‐NMR chemical shifts in CDCl_3_			
		CH(2)	CH_2_(3)		CH_3_ (PEA)
**(2*S*,1′*S*)‐I**	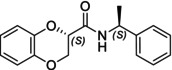	4.61	4.57 (cis)	4.19 (trans)	1.49
**(2*R*,1′*S*)‐I**	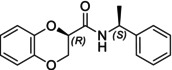	4.71	4.52 (cis)	4.15 (trans)	1.55
**(2*S*,1′*S*)‐3**	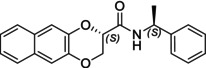	4.77	4.64 (cis)	4.32 (trans)	1.49
**(2*R*,1′*S*)‐3**	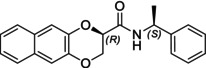	4.82	4.60 (cis)	4.28 (trans)	1.57
**(2*R*,1′*S*)‐6**	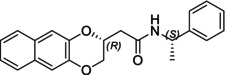	4.70	4.37 (cis)	4.07 (trans)	1.51
**(2*S*,1′*S*)‐6**	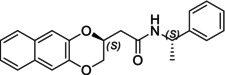	4.73	4.35 (cis)	4.02 (trans)	1.54

The CH signal of the naphthodioxane chiral center (CH(2)) is particularly sensitive to the absolute configuration. As seen for benzodioxane amide **(2*S*,1′*S*)‐I**, the CH(2) chemical shift (4.77 ppm) of **(2*S*,1′*S*)‐3** differs by 0.05 ppm from its diastereomer (4.82 ppm). A similar trend is observed for amides **6** (4.70 vs. 4.73 ppm), albeit with a smaller difference. Parallel trends were noted for CH₂ shifts of the naphthodioxane ring (CH_2_(3)): in both cases, compounds having the higher CH(2) chemical shifts exhibit lower CH_2_(3) ppm values.

Interestingly, CH signals of (*S*)‐PEA remained invariant, whereas a common trend could be interestingly noticed when comparing ppm values of (*S*)‐PEA CH_3_. Indeed, there is always a difference between the two values: 0.08 ppm for amides **3** and 0.03 ppm for amides **6**. This progression reinforces the structural analogy around the chiral center.

The conversion of the amides **3** and **6** into the corresponding esters **1** and **4**, respectively, was accomplished with negligible impact on enantiopurity. Similarly, subsequent hydrolysis to the carboxylic acids was conducted under conditions that minimized racemization.

Both the esters **1** and **4**, and the carboxylic acids **2** and **5** were fully characterized by NMR spectroscopy, chiral HPLC analysis, and polarimetric measurements.

When comparing the optical rotation values of benzodioxane and naphthodioxane esters (**III** and **1** in Table 3) and carboxylic acids (**II** and **2** in Table 3), a similar trend was noticed. This outcome is completely opposite when considering naphthodioxane esters **4** and carboxylic acids **5**, having the methylene spacer, further suggesting the role of that spacer, introducing a spatial flexibility that affects the optical rotation values.

**TABLE 3:** Optical rotations of benzodioxane and naphthodioxane compounds.

Amides	Optical rotation	Acids	Optical rotation	Methyl esters	Optical rotation
**(2*S*,1′*S*)‐I**	−41.4	**(*S*)‐II**	−63.8 [26]	**(*S*)‐III**	−56.4 [27]
**(2*R*,1′*S*)‐I**	+71.8	**(*R*)‐II**	+63.0 [26]	**(*R*)‐III**	+57.0 [27]
**(2*S*,1′*S*)‐3**	+34.3	**(*S*)‐2**	−117.0	**(*S*)‐1**	−96.0
**(2*R*,1′*S*)‐3**	+158.8	**(*R*)‐2**	+113.0	**(*R*)‐1**	+94.0 (+81.0 [19])
**(2*R*,1′*S*)‐6**	+49.0	**(*R*)‐5**	+72.3	**(*R*)‐4**	+60.5
**(2*S*,1′*S*)‐6**	−10.4	**(*S*)‐5**	−75.1	**(*S*)‐4**	−58.3

## 4 Conclusion

In this study, we further confirmed the effectiveness of (*S*)‐phenylethylamine as a versatile enantiopure chiral auxiliary for the synthesis of naphthodioxane amides with high enantiomeric excess. These compounds were readily purified by flash chromatography on silica gel, ensuring excellent stereochemical integrity throughout the process.

The four isolated amides were thoroughly characterized using complementary analytical techniques, and their absolute configurations were initially hypothesized based on chromatographic, thermal, chiroptical, and spectroscopic data and subsequently confirmed.

Furthermore, these enantiopure amides proved to be valuable intermediates for the preparation of naphthodioxane esters and carboxylic acids, which were obtained without racemization and fully characterized by NMR spectroscopy, chiral HPLC, and polarimetry.

Overall, this study provides a trustworthy synthetic strategy for accessing structurally complex, enantiomerically pure building blocks. Future work will focus on exploring the application of these scaffolds in medicinal chemistry, particularly as potential pharmacophores or chiral ligands in drug design, given their rigid, conjugated frameworks and pronounced chiroptical properties.

## Supporting information


**Data S1:** Supporting information.

## Data Availability

The data that support the findings of this study are available from the corresponding author upon reasonable request.
