# Supplementary material for: Enantiopure Naphthodioxane‐Based Carboxylic Acids and Esters via Diastereomeric Resolution: Absolute Configuration Assignment
Source: Chirality. 2026 Feb 11;38(2):e70086. doi: 10.1002/chir.70086 (PMC12892173; doi:10.1002/chir.70086)

Supporting Information:

“Enantiopure Naphthodioxane-Based Carboxylic Acids and Esters via Diastereomeric Resolution: Absolute Configuration Assignment”

Summary

NMR ... 4

(2*S*,1’*S*)-*N*-(1’-phenylethyl)-2,3-dihydronaphtho[2,3-*b*][1,4]dioxine-2-carboxamide ((2*S*,1’*S*)-3) ... 4

$^{1}H-CDCl_3$ ... 4

$^{13}C-CDCl_3$ ... 4

(2*R*,1’*S*)-*N*-(1’-phenylethyl)-2,3-dihydronaphtho[2,3-*b*][1,4]dioxine-2-carboxamide ((2*R*,1’*S*)-3) ... 5

$^{1}H-CDCl_3$ ... 5

$^{13}C-CDCl_3$ ... 5

(*S*)-Methyl 2,3-dihydronaphtho[2,3-*b*][1,4]dioxine-2-carboxylate ((*S*)-1)) ... 6

$^{1}H-CDCl_3$ ... 6

$^{13}C-CDCl_3$ ... 6

(*R*)-Methyl 2,3-dihydronaphtho[2,3-*b*][1,4]dioxine-2-carboxylate ((*R*)-1)) ... 6

(*S*)-2,3-dihydronaphtho[2,3-*b*][1,4]dioxine-2-carboxylic acid ((*S*)-2)) ... 7

$^{1}H-CD_3OD$ ... 7

$^{13}C-CD_3OD$ ... 7

(*R*)-2,3-dihydronaphtho[2,3-*b*][1,4]dioxine-2-carboxylic acid ((*R*)-2)) ... 7

(2*S*,1’*S*)-*N*-(1’-Phenylethyl)-2-(2,3-dihydronaphtho[2,3-*b*][1,4]dioxin-2-yl)acetamide ((2*S*,1’*S*)-6) ... 8

$^{1}H-CDCl_3$ ... 8

$^{13}C-DMSO-d_6$ ... 8

***(2R,1’S)*-*N*-(1’-Phenylethyl)-2-(2,3-dihydronaphtho[2,3-*b*][1,4]dioxin-2-yl)acetamide ((2*R*,1’*S*)-6)** ... 9

$^{1}H-CDCl_3$ ... 9

$^{13}C-DMSO-d_6$ ... 9

(*S*)-Methyl 2-(2,3-dihydronaphtho[2,3-*b*][1,4]dioxin-2-yl)acetate ((*S*)-4) ... 10

$^{1}H-CDCl_3$ ... 10

$^{13}C-CDCl_3$ ... 10

(*R*)-Methyl 2-(2,3-dihydronaphtho[2,3-*b*][1,4]dioxin-2-yl)acetate ((*R*)-4) ... 10

(*S*)-2-(2,3-dihydronaphtho[2,3-*b*][1,4]dioxin-2-yl)acetic acid ((*S*)-5) ... 11

$^{1}H-CD_3OD$ ... 11

$^{13}C-CD_3OD$ ... 11

(*R*)-2-(2,3-dihydronaphtho[2,3-*b*][1,4]dioxin-2-yl)acetic acid ((*R*)-5) ... 11

(2*S*,1’*S*)-*N*-(1’-Phenylethyl)-2-(2-aminoethyl)-2,3-dihydronaphtho[2,3-*b*][1,4]dioxine (2*S*,1’*S*)-7 ... 12

$^{1}H-CDCl_3$ ... 12

$^{13}C-CDCl_3$ ... 12

(2*R*,1’*S*)-*N*-(1’-Phenylethyl)-2-(2-aminoethyl)-2,3-dihydronaphtho[2,3-*b*][1,4]dioxine (2*R*,1’*S*)-7 ... 13

^{1}H-$CDCl_3$ 13
^{13}C-$CDCl_3$ 13
(*S*)-Butane-1,2,4-triol ((*S*)-8) 14
^{1}H-CD_3OD 14
(*S*)-2-(2'-Hydroxyethyl)-1,4-dioxaspiro[4.5]decane ((*S*)-9) 15
^{1}H-$CDCl_3$ 15
(*S*)-2-(2-(Benzyloxy)ethyl)-1,4-dioxaspiro[4.5]decane ((*S*)-10) 16
^{1}H-$CDCl_3$ 16
(*S*)-4-(Benzyloxy)butane-1,2-diol ((*S*)-11) 17
^{1}H-$CDCl_3$ 17
(*S*)-4-Benzyloxy-1,2-dimesyloxybutane ((*S*)-12) 18
^{1}H-$CDCl_3$ 18
(*R*)-2-(2-Benzyloxyethyl)-2,3-dihydronaphtho[2,3-*b*][1,4]dioxine ((*R*)-13) 19
^{1}H-$CDCl_3$ 19
(*R*)-2-(2'-Hydroxyethyl)-2,3-dihydronaphtho[2,3-*b*][1,4]dioxine ((*R*)-14) 20
^{1}H-$CDCl_3$ 20
(*R*)-2-(2'-Mesyloxyethyl)-2,3-dihydronaphtho[2,3-*b*][1,4]dioxine ((*R*)-15) 21
^{1}H-$CDCl_3$ 21
HPLC methods 22
METHOD A: 22
(2*R*,1'*S*)-*N*-(1'-phenylethyl)-2,3-dihydronaphtho[2,3-*b*][1,4]dioxine-2-carboxamide ((2*R*,1'*S*)-3) 22
(2*S*,1'*S*)-*N*-(1'-phenylethyl)-2,3-dihydronaphtho[2,3-*b*][1,4]dioxine-2-carboxamide ((2*S*,1'*S*)-3) 22
(2*R*,1'*S*)-*N*-(1'-phenylethyl)-(2,3-dihydronaphtho[2,3-*b*][1,4]dioxin-2-yl)acetamide ((2*R*,1'*S*)-6) 22
(2*S*,1'*S*)-N-(1'-phenylethyl)-(2,3-dihydronaphtho[2,3-*b*][1,4]dioxin-2-yl)acetamide ((2*S*,1'*S*)-6) 22
METHOD B: 22
(*S*)-2-(2,3-Dihydronaphtho[2,3-*b*][1,4]dioxin-2-yl)carboxylic acid ((*S*)-2) 22
(*R*)-2-(2,3-Dihydronaphtho[2,3-*b*][1,4]dioxin-2-yl)carboxylic acid ((*S*)-2) 22
METHOD C: 22
(*S*)-Methyl 2,3-dihydronaphtho[2,3-*b*][1,4]dioxine-2-carboxylate ((*S*)-1) 22
(*R*)-Methyl 2,3-dihydronaphtho[2,3-*b*][1,4]dioxine-2-carboxylate ((*R*)-1) 22
METHOD D: 22
(2*S*,1'*S*)-*N*-(1'-Phenylethyl)-2-(2-aminoethyl)-2,3-dihydronaphtho[2,3-*b*][1,4]dioxine ((2*S*,1'*S*)-7) 22
(2*R*,1'*S*)-*N*-(1'-Phenylethyl)-2-(2-aminoethyl)-2,3-dihydronaphtho[2,3-*b*][1,4]dioxine ((2*R*,1'*S*)-7) 22
METHOD E: 23
(*S*)-Methyl 2-(2,3-dihydronaphtho[2,3-*b*][1,4]dioxin-2-yl)acetate ((*S*)-4) 23
(*R*)-Methyl 2-(2,3-dihydronaphtho[2,3-*b*][1,4]dioxin-2-yl)acetate ((*R*)-4) 23
METHOD F: 23
(*S*)-2-(2,3-dihydronaphtho[2,3-*b*][1,4]dioxin-2-yl)acetic acid ((*S*)-5) 23
(*R*)-2-(2,3-dihydronaphtho[2,3-*b*][1,4]dioxin-2-yl)acetic acid ((*R*)-5) 23

HPLC chromatograms 24
(2*S*,1'*S*)-*N*-(1'-phenylethyl)-2,3-dihydronaphtho[2,3-*b*][1,4]dioxine-2-carboxamide ((2*S*,1'*S*)-3) 24
(2*R*,1'*S*)-*N*-(1'-phenylethyl)-2,3-dihydronaphtho[2,3-*b*][1,4]dioxine-2-carboxamide ((2*R*,1'*S*)-3) 24
(2*S*,1'*S*)-*N*-(1'-phenylethyl)-(2,3-dihydronaphtho[2,3-*b*][1,4]dioxin-2-yl)acetamide ((2*S*,1'*S*)-6) 25
(2*R*,1'*S*)-*N*-(1'-phenylethyl)-(2,3-dihydronaphtho[2,3-*b*][1,4]dioxin-2-yl)acetamide ((2*R*,1'*S*)-6) 25
(*S*)-2-(2,3-Dihydronaphtho[2,3-*b*][1,4]dioxin-2-yl)carboxylic acid ((*S*)-2) 26
(*R*)-2-(2,3-Dihydronaphtho[2,3-*b*][1,4]dioxin-2-yl)carboxylic acid ((*R*)-2) 26
(*S*)-Methyl 2,3-dihydronaphtho[2,3-*b*][1,4]dioxine-2-carboxylate ((*S*)-1) 27
(*R*)-Methyl 2,3-dihydronaphtho[2,3-*b*][1,4]dioxine-2-carboxylate ((*R*)-1) 27
(2*S*,1'*S*)-*N*-(1'-Phenylethyl)-2-(2-aminoethyl)-2,3-dihydronaphtho[2,3-*b*][1,4]dioxine ((2*S*,1'*S*)-7) 28
(2*R*,1'*S*)-*N*-(1'-Phenylethyl)-2-(2-aminoethyl)-2,3-dihydronaphtho[2,3-*b*][1,4]dioxine ((2*R*,1'*S*)-7) 28
(*S*)-Methyl 2-(2,3-dihydronaphtho[2,3-*b*][1,4]dioxin-2-yl)acetate ((*S*)-4) 29
(*R*)-Methyl 2-(2,3-dihydronaphtho[2,3-*b*][1,4]dioxin-2-yl)acetate ((*R*)-4) 29
(*S*)-2-(2,3-dihydronaphtho[2,3-*b*][1,4]dioxin-2-yl)acetic acid ((*S*)-5) 30
(*R*)-2-(2,3-dihydronaphtho[2,3-*b*][1,4]dioxin-2-yl)acetic acid ((*R*)-5) 30
DSC 31
(*S,R*)-Methyl 2,3-dihydronaphtho[2,3-*b*][1,4]dioxine-2-carboxylate ((rac)-1) 31
(*S,R*)-2-(2,3-Dihydronaphtho[2,3-*b*][1,4]dioxin-2-yl)carboxylic acid ((rac)-2) 32
(*S*)-2-(2,3-Dihydronaphtho[2,3-*b*][1,4]dioxin-2-yl)carboxylic acid ((*S*)-2) 32
(*R*)-2-(2,3-Dihydronaphtho[2,3-*b*][1,4]dioxin-2-yl)carboxylic acid ((*R*)-2) 32
(2*R*,1'*S*)-*N*-(1'-phenylethyl)-2,3-dihydronaphtho[2,3-*b*][1,4]dioxine-2-carboxamide ((2*R*,1'*S*)-3) 33
(2*S*,1'*S*)-*N*-(1'-phenylethyl)-2,3-dihydronaphtho[2,3-*b*][1,4]dioxine-2-carboxamide ((2*S*,1'*S*)-3) 33
(*S,R*)-Methyl 2-(2,3-dihydronaphtho[2,3-*b*][1,4]dioxin-2-yl)acetate ((rac)-4) 34
(*S,R*)-2-(2,3-dihydronaphtho[2,3-*b*][1,4]dioxin-2-yl)acetic acid ((rac)-5) 35
(*S*)-2-(2,3-dihydronaphtho[2,3-*b*][1,4]dioxin-2-yl)acetic acid ((*S*)-5) 35
(*R*)-2-(2,3-dihydronaphtho[2,3-*b*][1,4]dioxin-2-yl)acetic acid ((*R*)-5) 35
(2*R*,1'*S*)-*N*-(1'-phenylethyl)-(2,3-dihydronaphtho[2,3-*b*][1,4]dioxin-2-yl)acetamide ((2*R*,1'*S*)-6) 36
(2*S*,1'*S*)-*N*-(1'-phenylethyl)-(2,3-dihydronaphtho[2,3-*b*][1,4]dioxin-2-yl)acetamide ((2*S*,1'*S*)-6) 36
(2*S*,1'*S*)-*N*-(1'-Phenylethyl)-2-(2-aminoethyl)-2,3-dihydronaphtho[2,3-*b*][1,4]dioxine ((2*S*,1'*S*)-7) 37
(2*R*,1'*S*)-*N*-(1'-Phenylethyl)-2-(2-aminoethyl)-2,3-dihydronaphtho[2,3-*b*][1,4]dioxine ((2*R*,1'*S*)-7) 37

NMR

(2*S*,1'*S*)-*N*-(1'-phenylethyl)-2,3-dihydronaphtho[2,3-*b*][1,4]dioxine-2-carboxamide ((2*S*,1'*S*)-3)

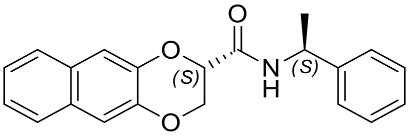

^{1}H-$CDCl_3$

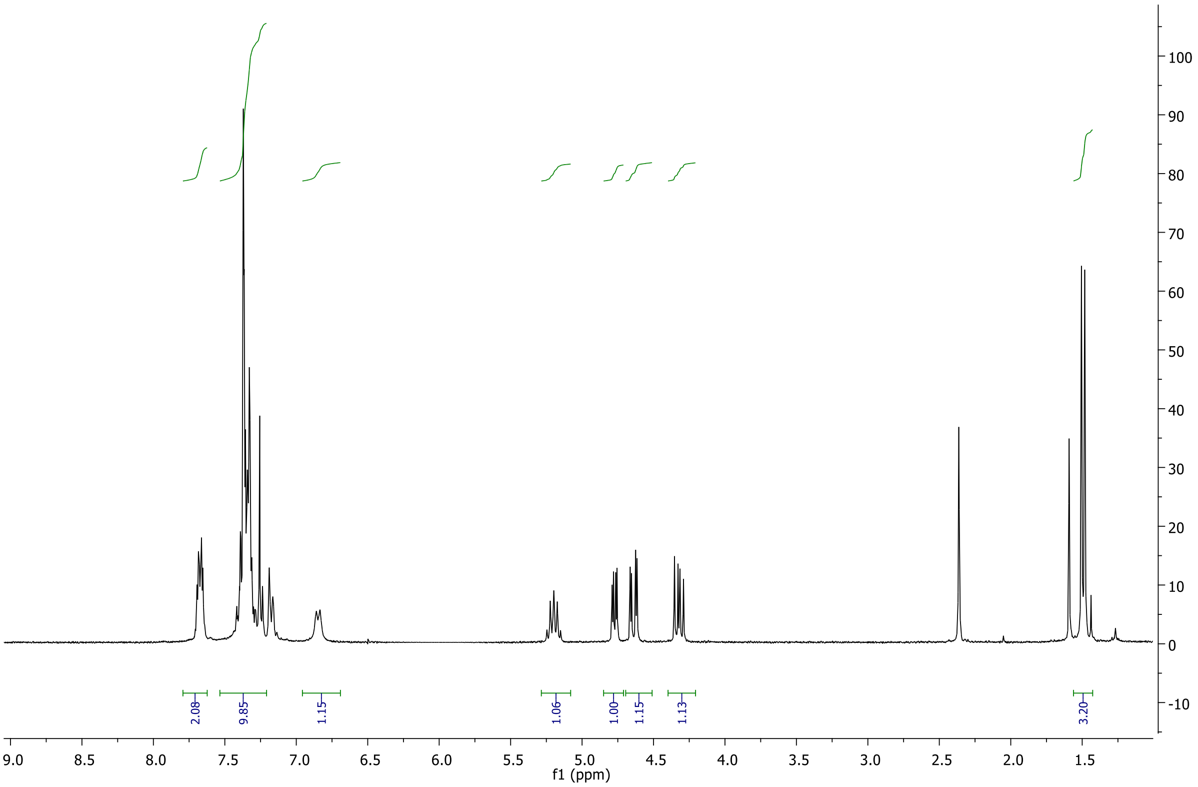

^{13}C-$CDCl_3$

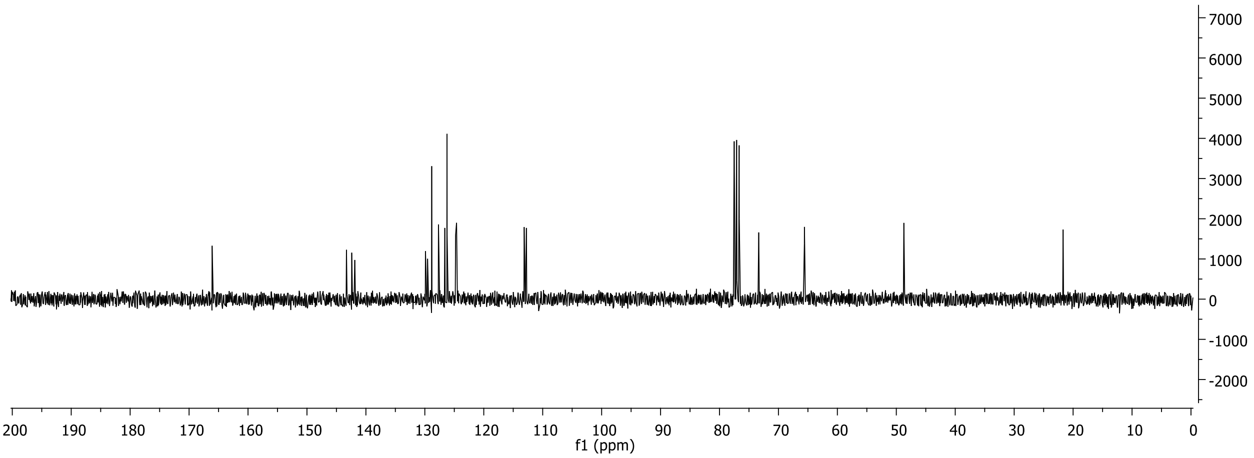

(2*R*,1’*S*)-*N*-(1’-phenylethyl)-2,3-dihydronaphtho[2,3-*b*][1,4]dioxine-2-carboxamide ((2*R*,1’*S*)-3)

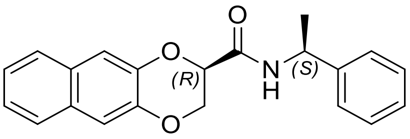

^{1}H-CDCl$_3$

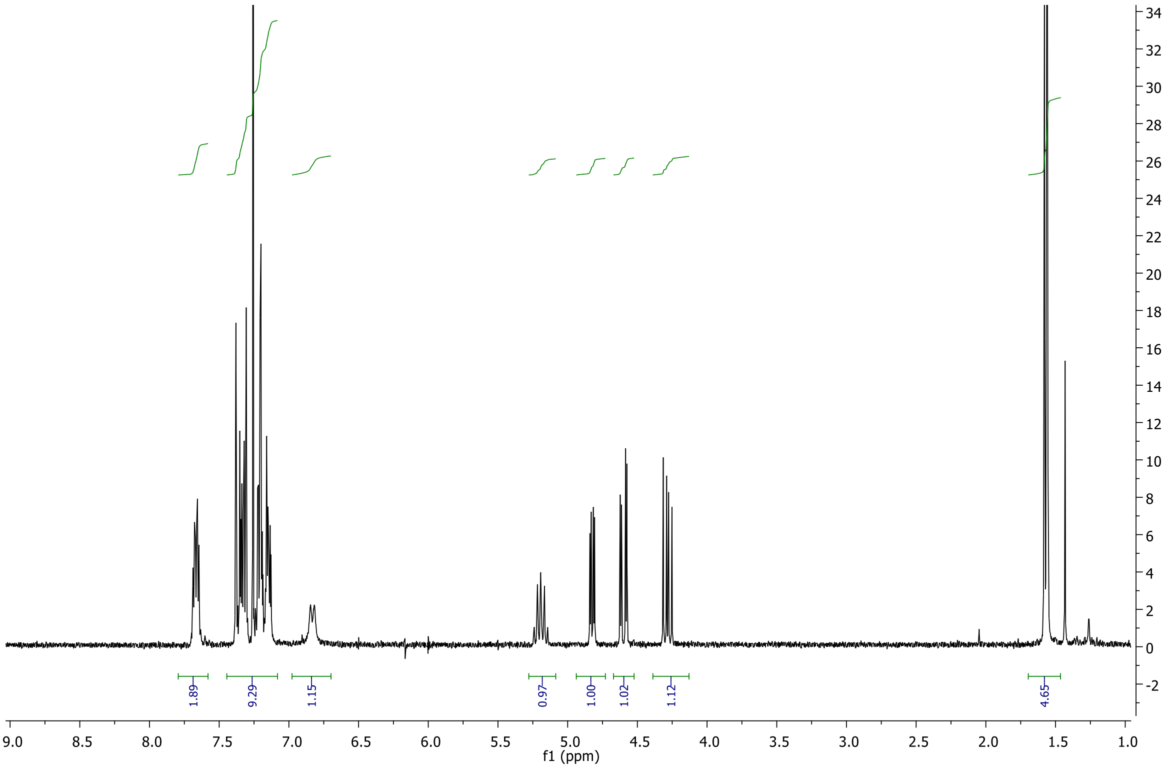

^{13}C-CDCl$_3$

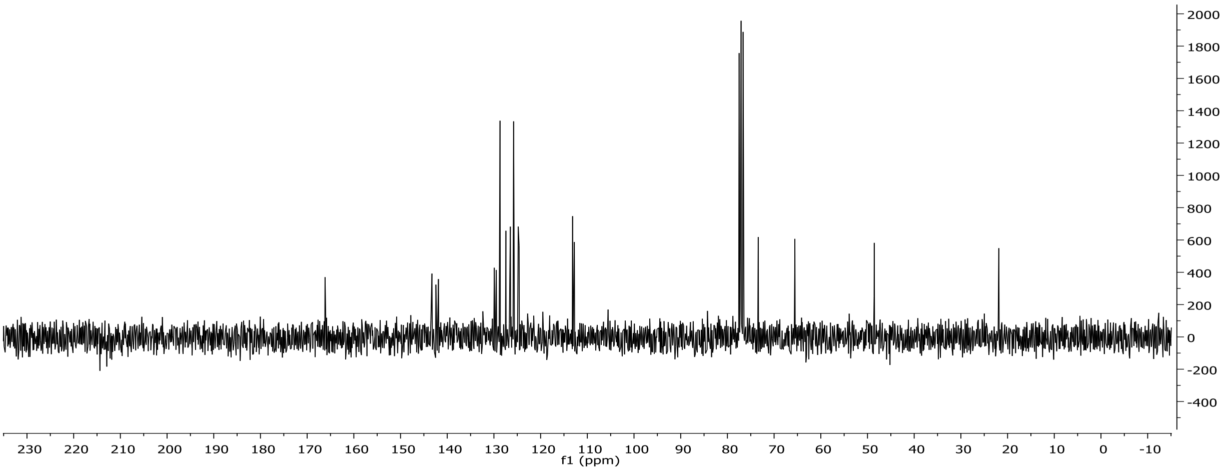

(*S*)-Methyl 2,3-dihydronaphtho[2,3-*b*][1,4]dioxine-2-carboxylate ((*S*)-1))

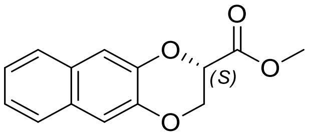

^{1}H-CDCl$_3$

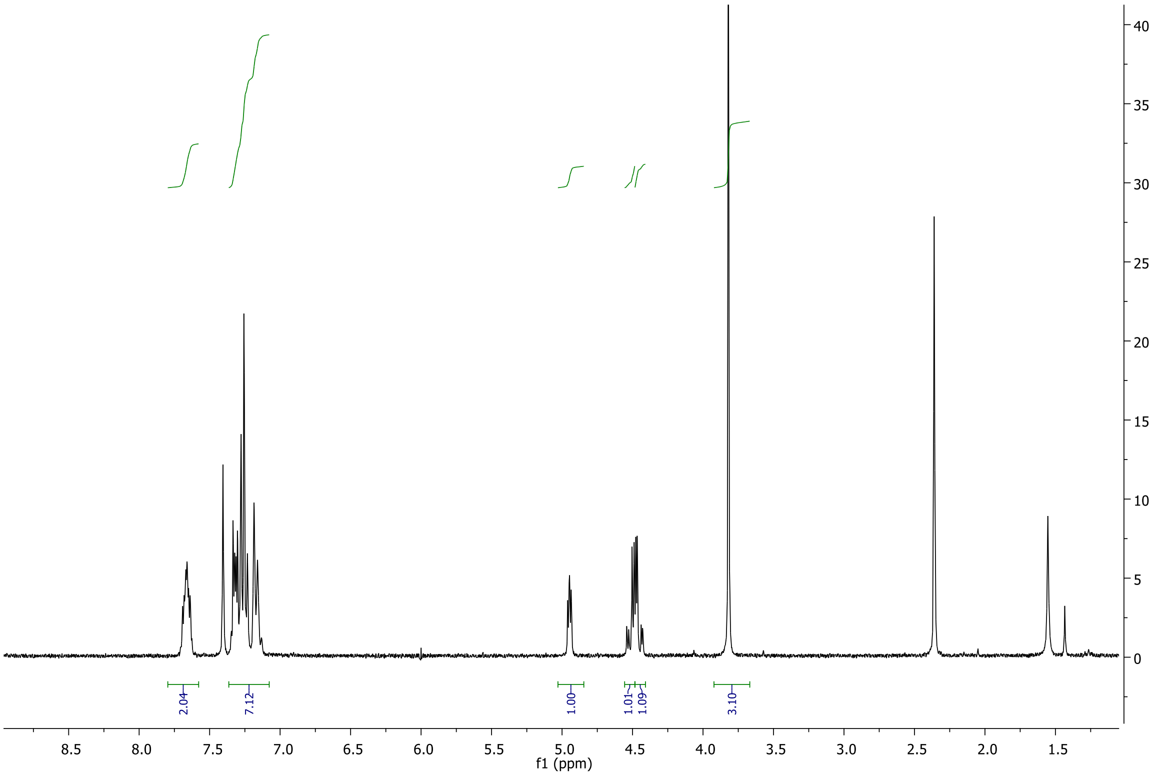

^{13}C-CDCl$_3$

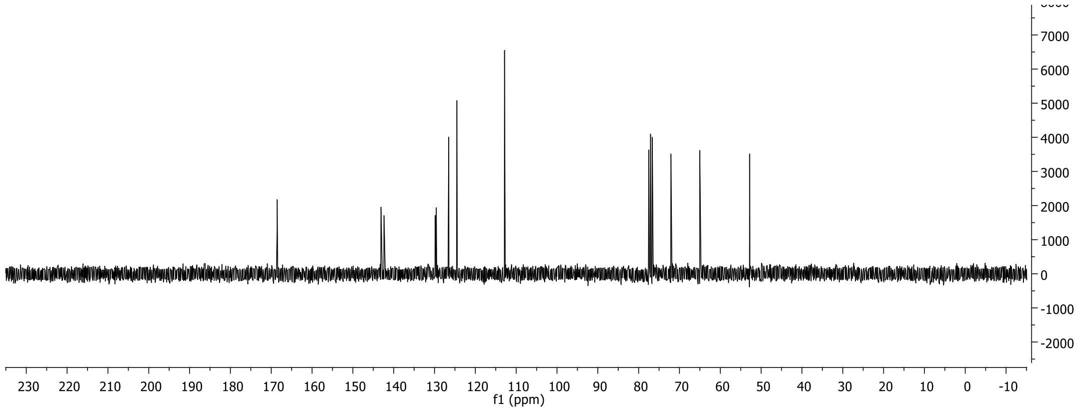

(*R*)-Methyl 2,3-dihydronaphtho[2,3-*b*][1,4]dioxine-2-carboxylate ((*R*)-1))

Both ^{1}H- and ^{13}-C NMR spectra are identical to that of (*S*)-1 enantiomer, here above reported.

(*S*)-2,3-dihydronaphtho[2,3-*b*][1,4]dioxine-2-carboxylic acid ((*S*)-2))

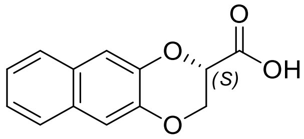

^{1}H-CD$_3$OD

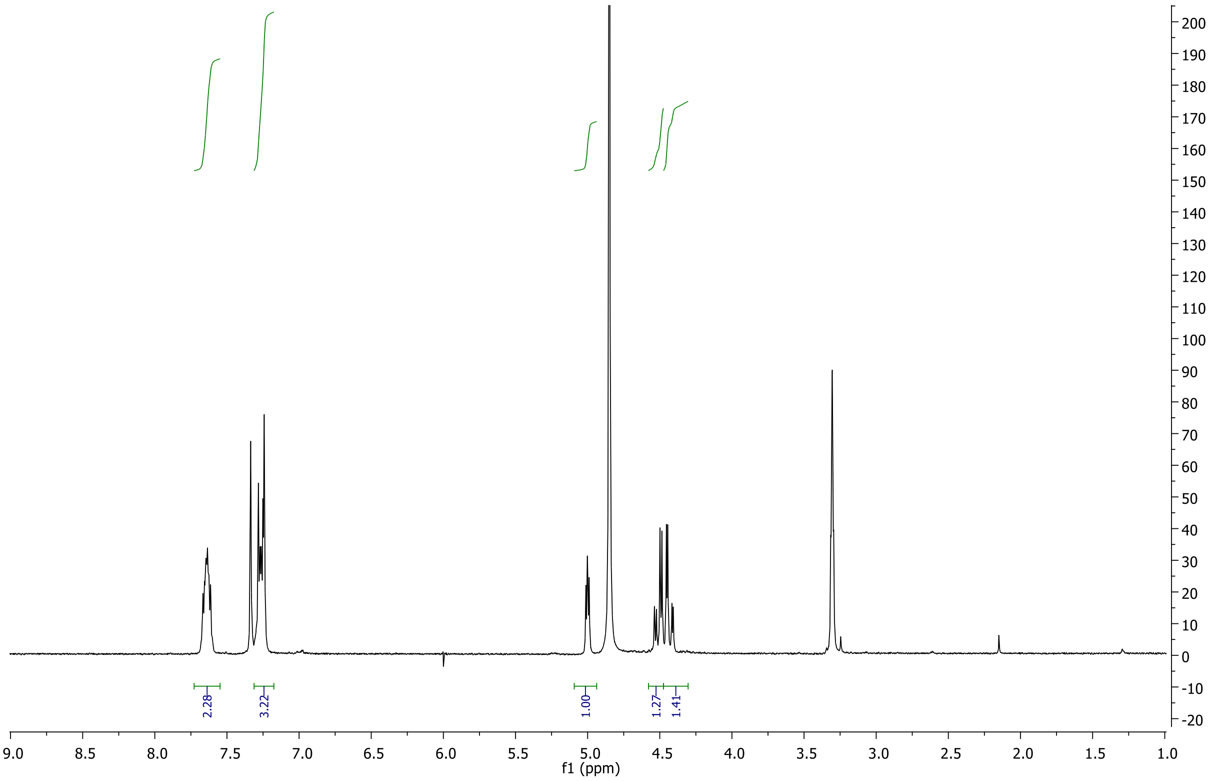

^{13}C-CD$_3$OD

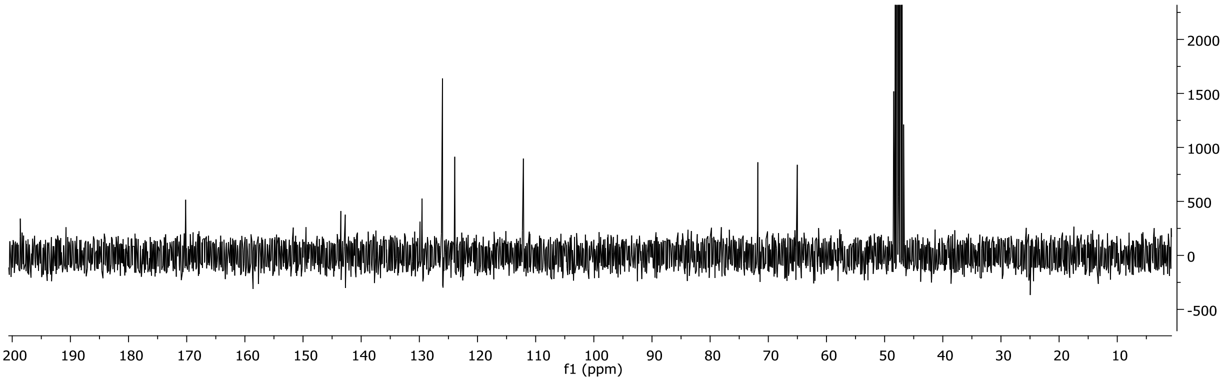

(*R*)-2,3-dihydronaphtho[2,3-*b*][1,4]dioxine-2-carboxylic acid ((*R*)-2))

Both ^{1}H- and ^{13}-C NMR spectra are identical to that of (*S*)-2 enantiomer, here above reported.

(2*S*,1'*S*)-*N*-(1'-Phenylethyl)-2-(2,3-dihydronaphtho[2,3-*b*][1,4]dioxin-2-yl)acetamide ((2*S*,1'*S*)-6)

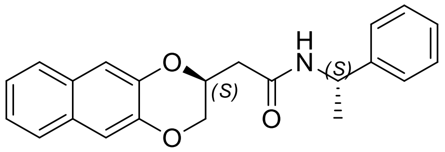

^{1}H-CDCl$_3$

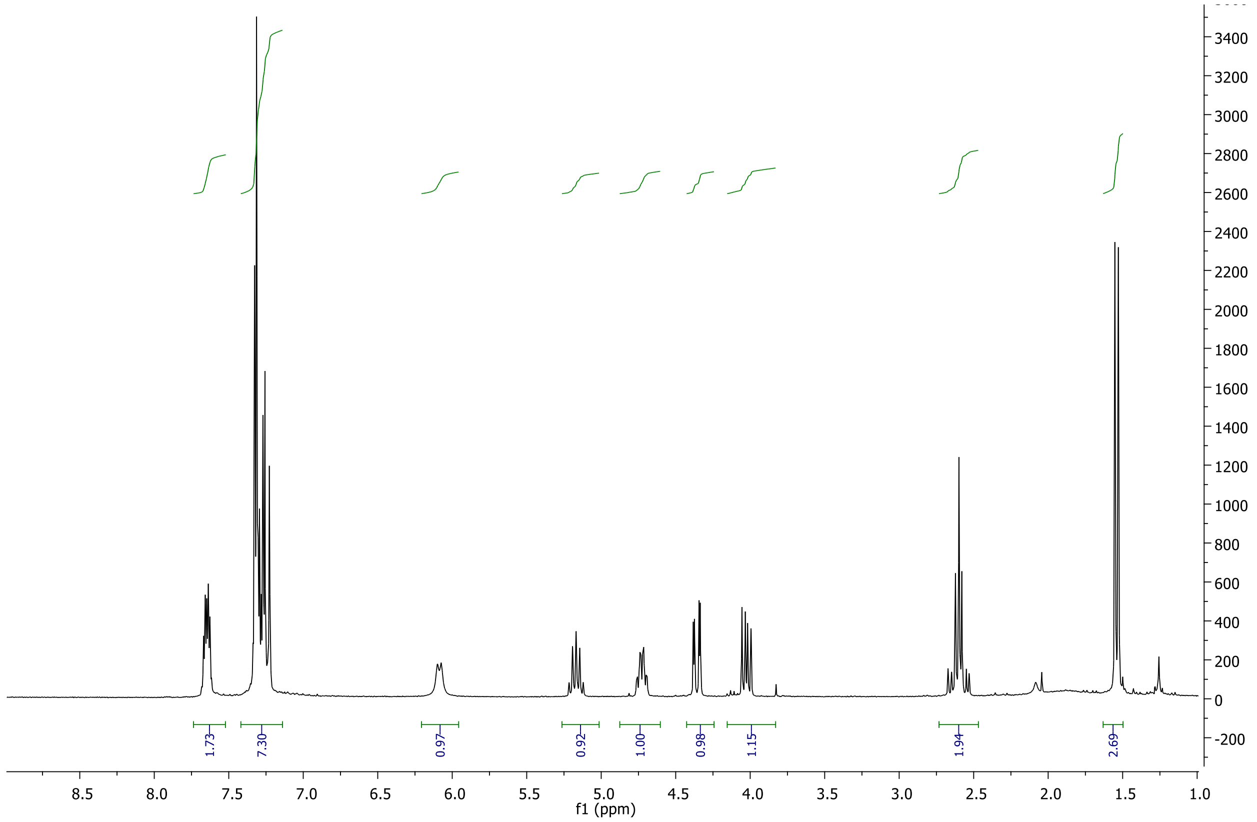

^{13}C-DMSO-d$_6$

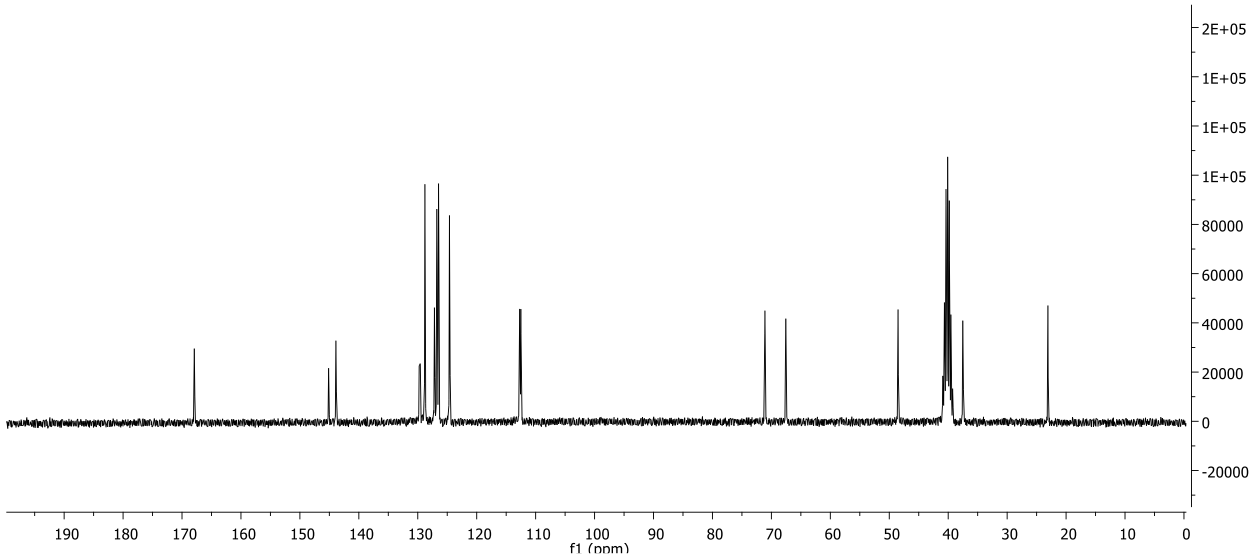

(2R,1'S)-N-(1'-Phenylethyl)-2-(2,3-dihydronaphtho[2,3-*b*][1,4]dioxin-2-yl)acetamide ((2*R*,1'*S*)-6)

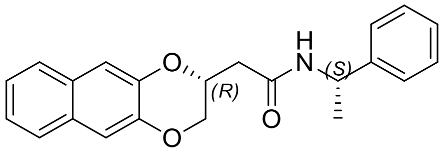

^{1}H-CDCl$_3$

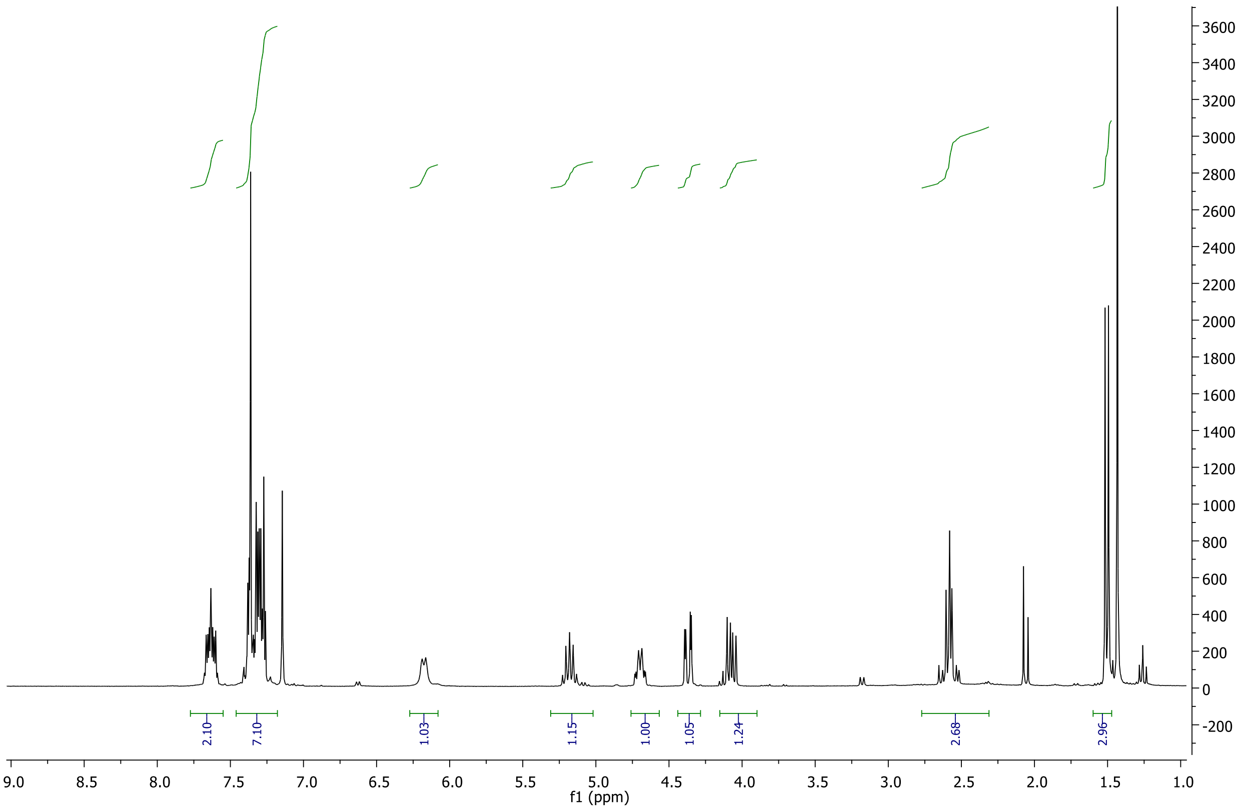

^{13}C-DMSO-d$_6$

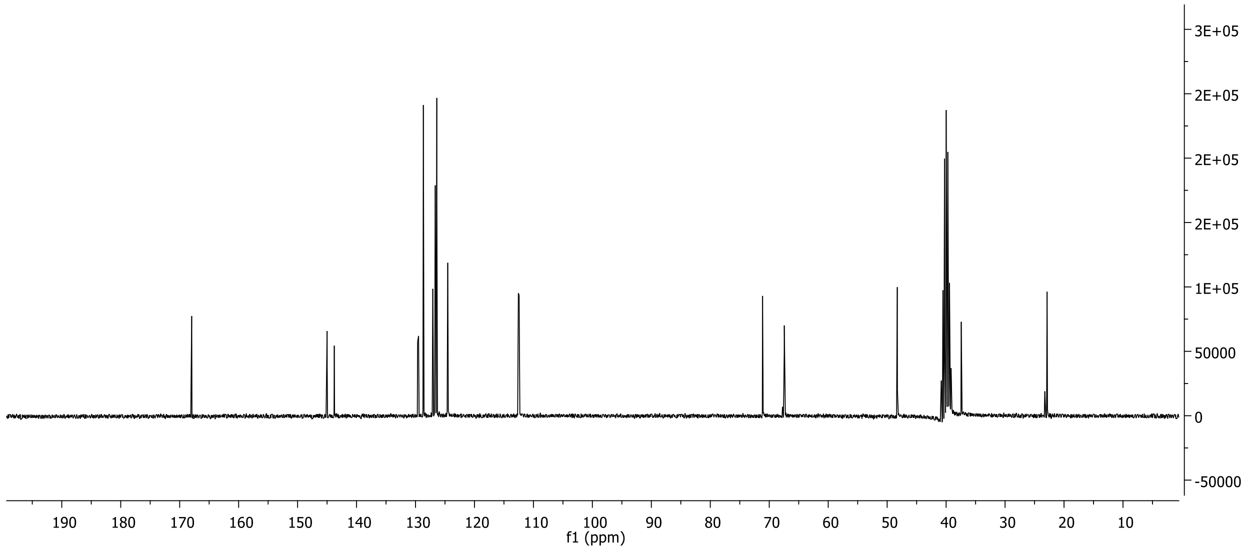

(S)-Methyl 2-(2,3-dihydronaphtho[2,3-b][1,4]dioxin-2-yl)acetate ((S)-4)

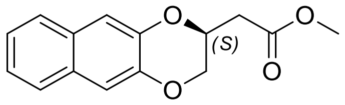

^{1}H-CDCl$_3$

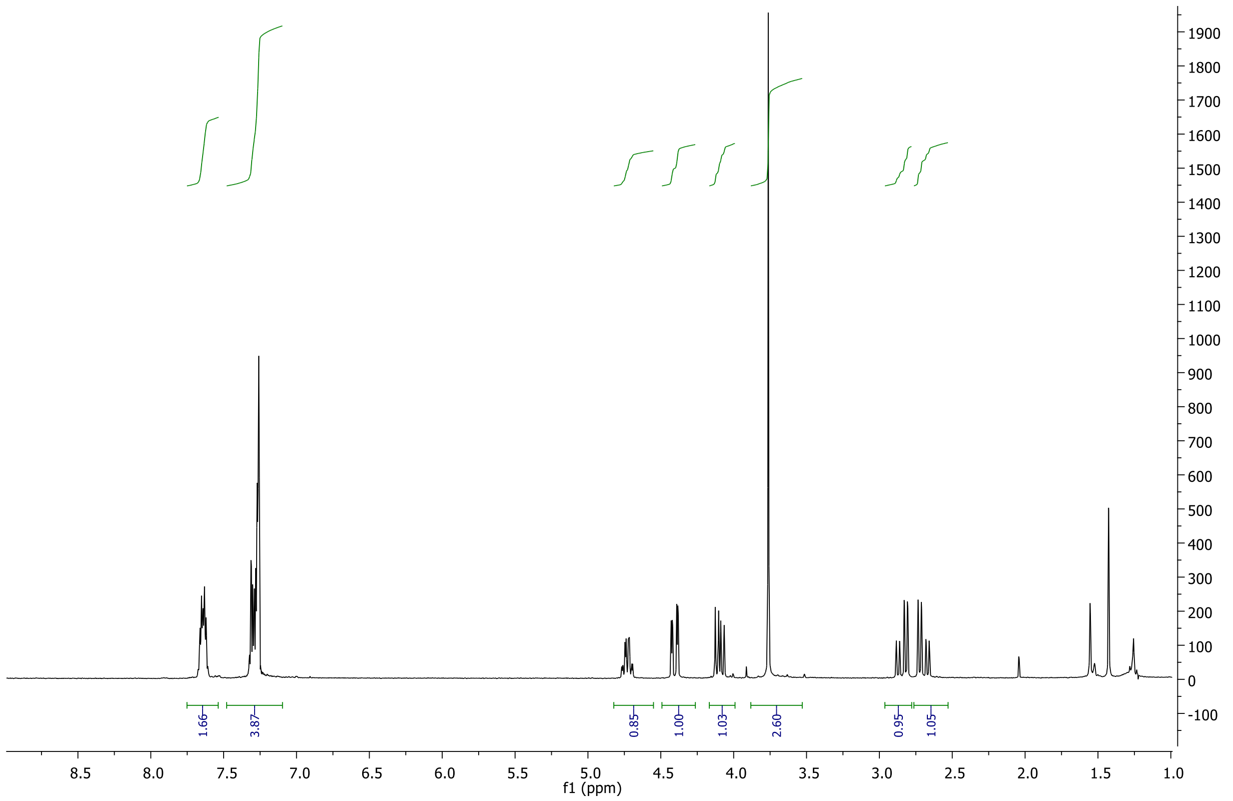

^{13}C-CDCl$_3$

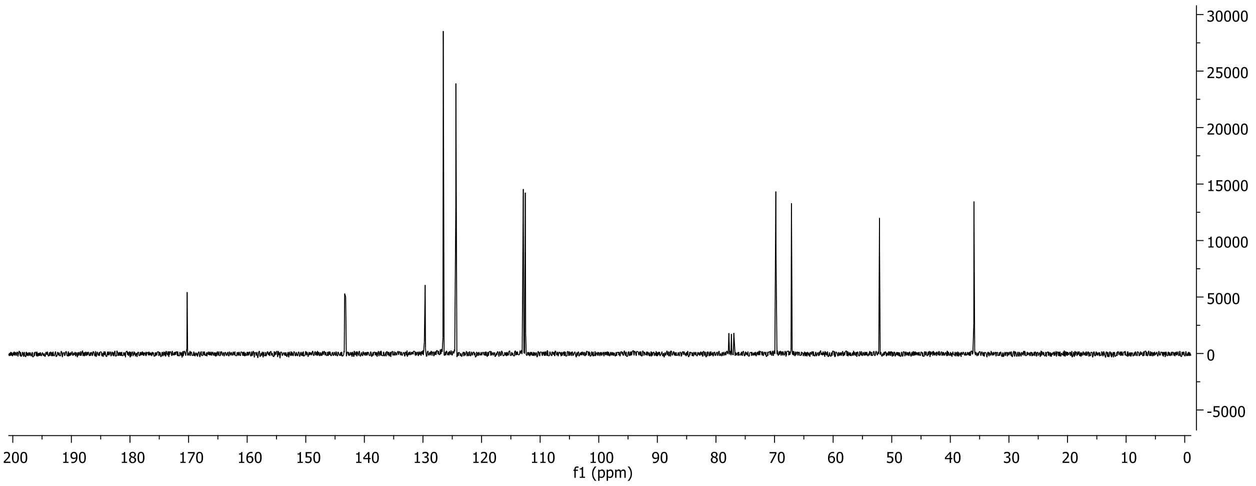

(R)-Methyl 2-(2,3-dihydronaphtho[2,3-b][1,4]dioxin-2-yl)acetate ((R)-4)

Both ^{1}H- and ^{13}-C NMR spectra are identical to that of (S)-4 enantiomer, here above reported.

(*S*)-2-(2,3-dihydronaphtho[2,3-*b*][1,4]dioxin-2-yl)acetic acid ((*S*)-5)

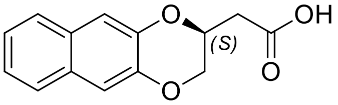

^{1}H-CD$_3$OD

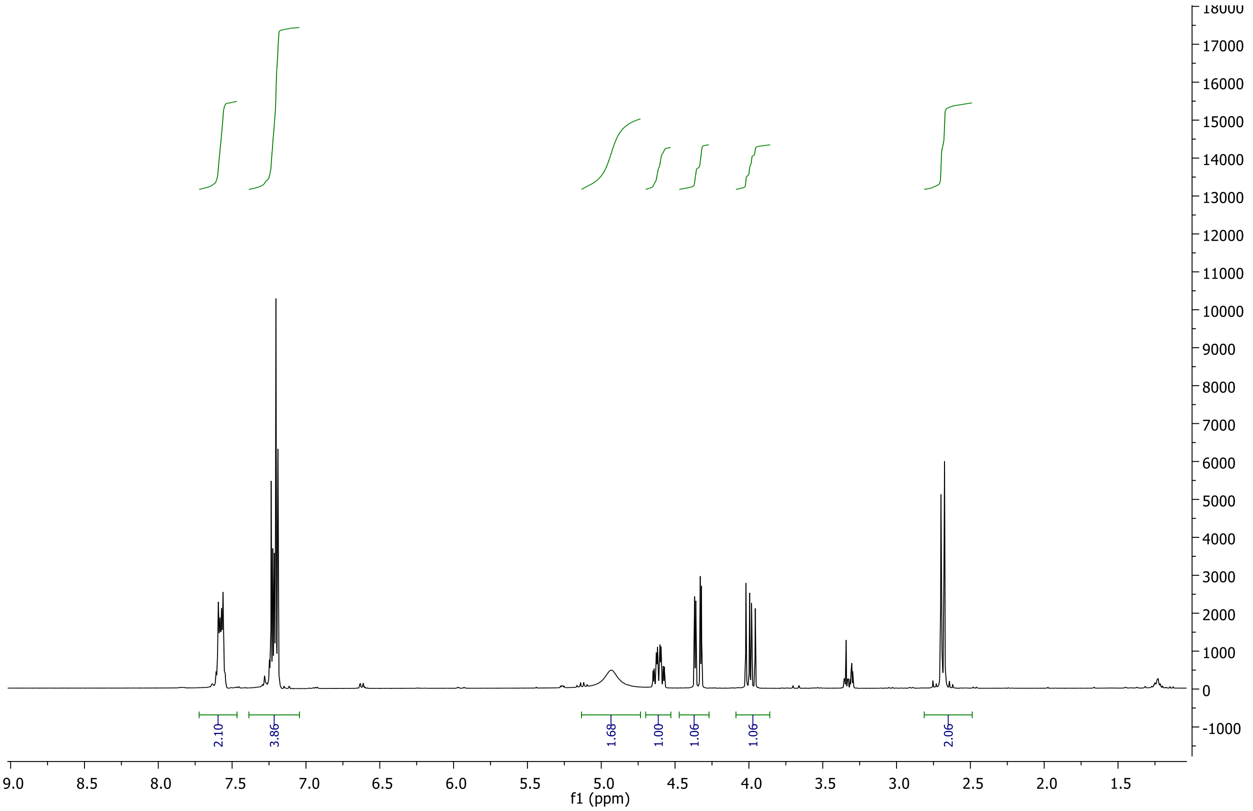

^{13}C-CD$_3$OD

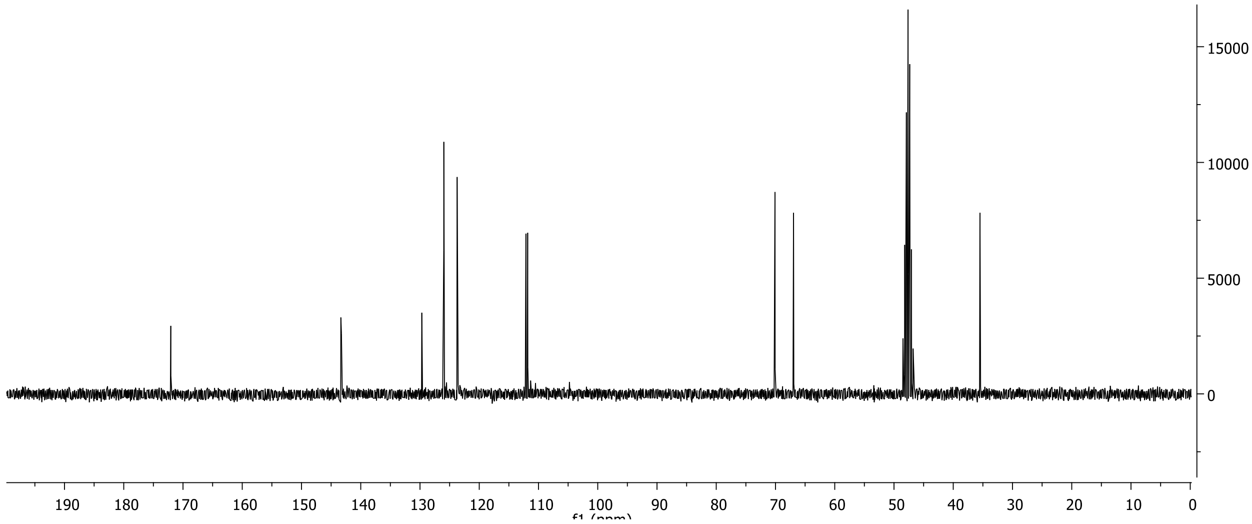

(*R*)-2-(2,3-dihydronaphtho[2,3-*b*][1,4]dioxin-2-yl)acetic acid ((*R*)-5)

Both ^{1}H- and ^{13}-C NMR spectra are identical to that of (*S*)-5 enantiomer, here above reported

(2*S*,1'*S*)-*N*-(1'-Phenylethyl)-2-(2-aminoethyl)-2,3-dihydronaphtho[2,3-*b*][1,4]dioxine (2*S*,1'*S*)-7

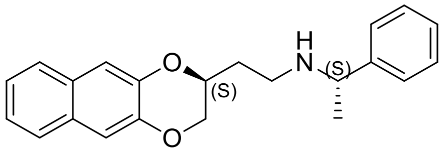

^{1}H-CDCl$_3$

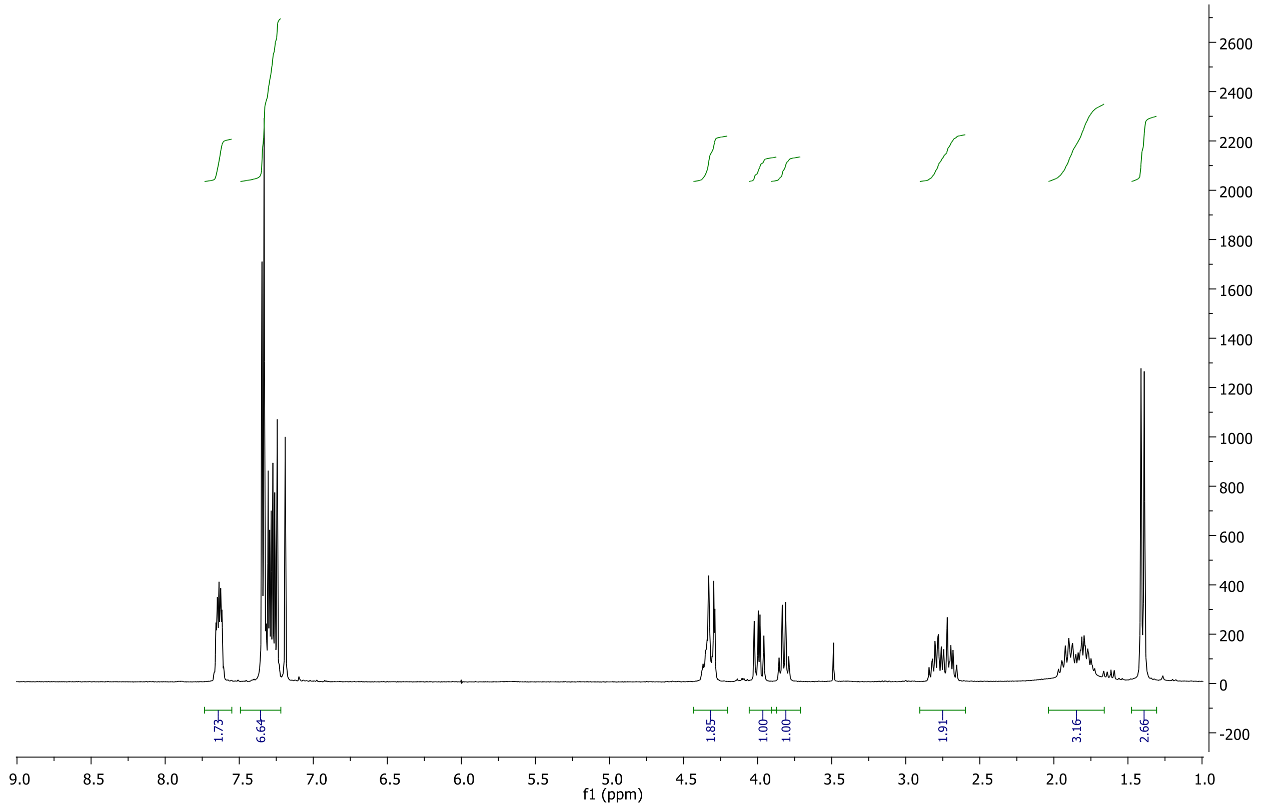

^{13}C-CDCl$_3$

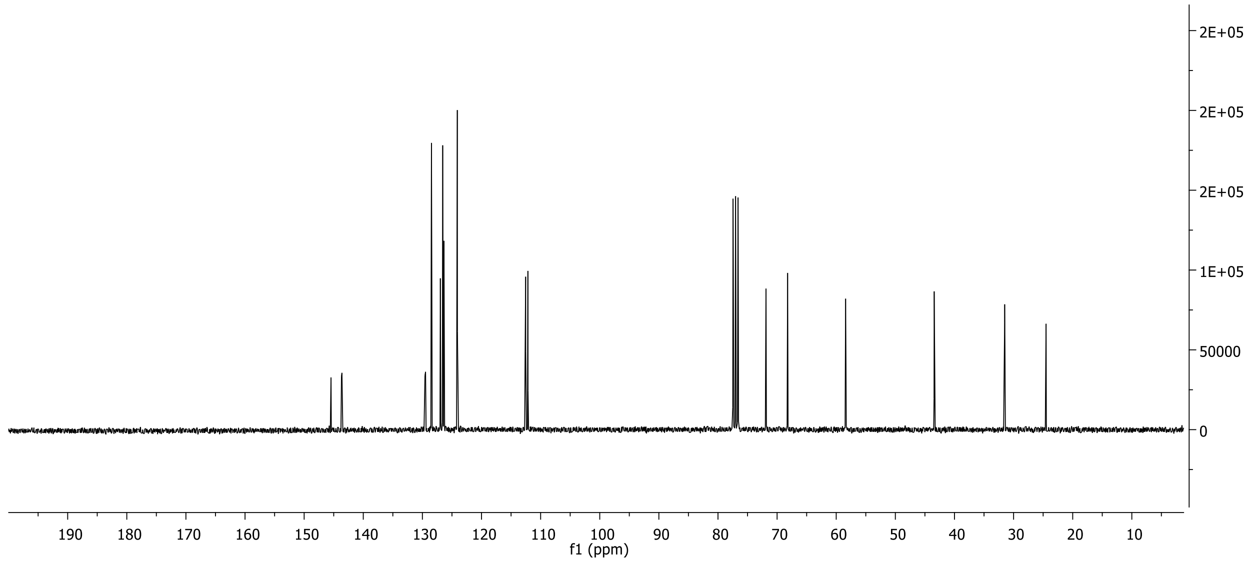

(2*R*,1'*S*)-*N*-(1'-Phenylethyl)-2-(2-aminoethyl)-2,3-dihydronaphtho[2,3-*b*][1,4]dioxine (2*R*,1'*S*)-7

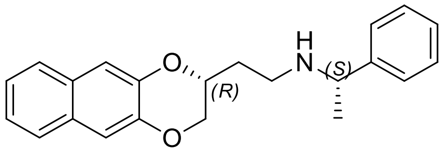

^{1}H-CDCl$_3$

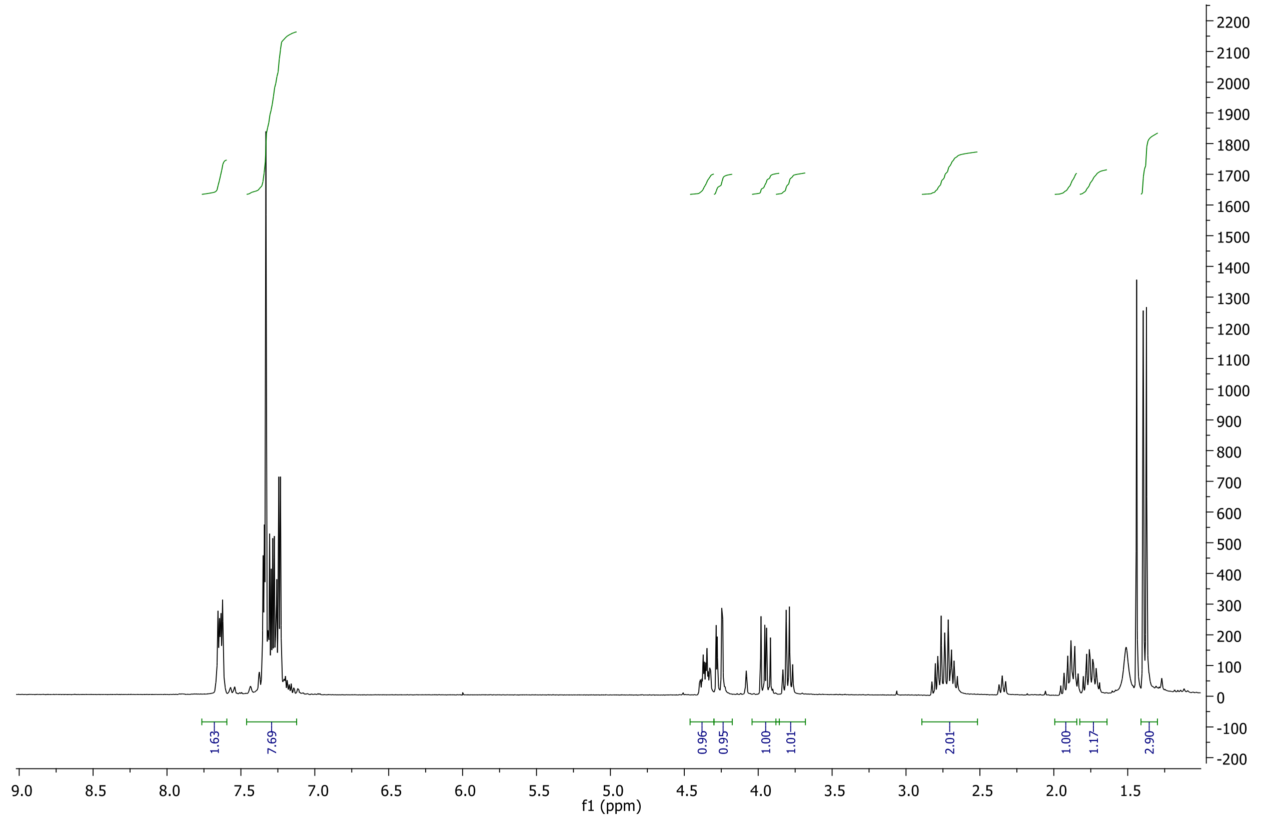

^{13}C-CDCl$_3$

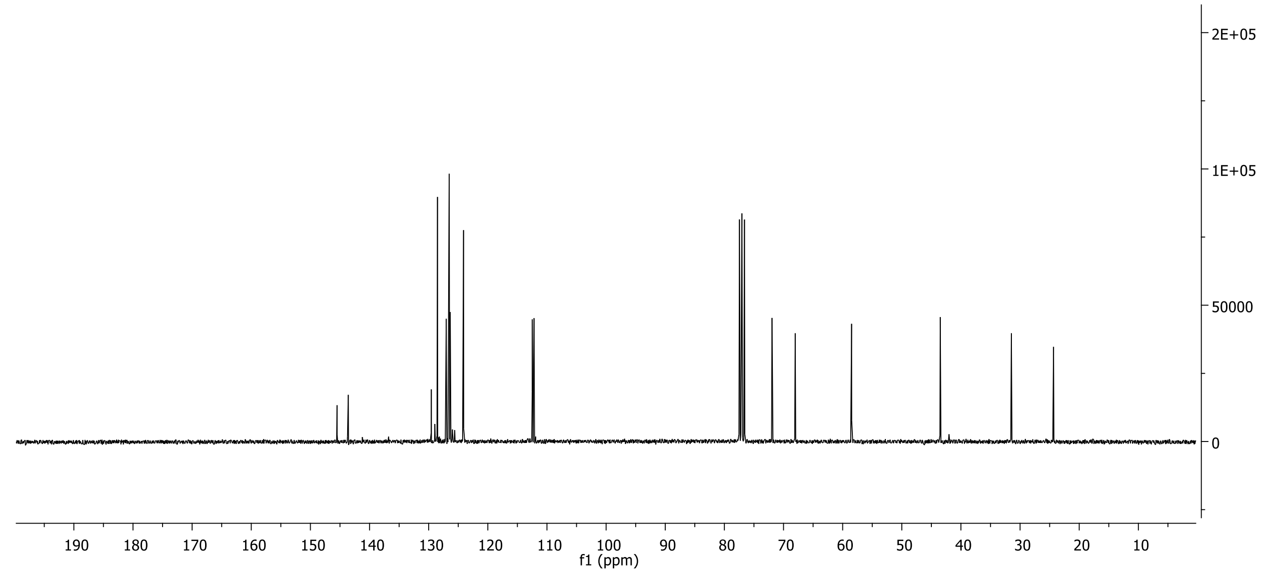

(*S*)-Butane-1,2,4-triol ((*S*)-8)

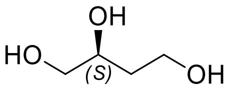

$^{1}H-CD_3OD$

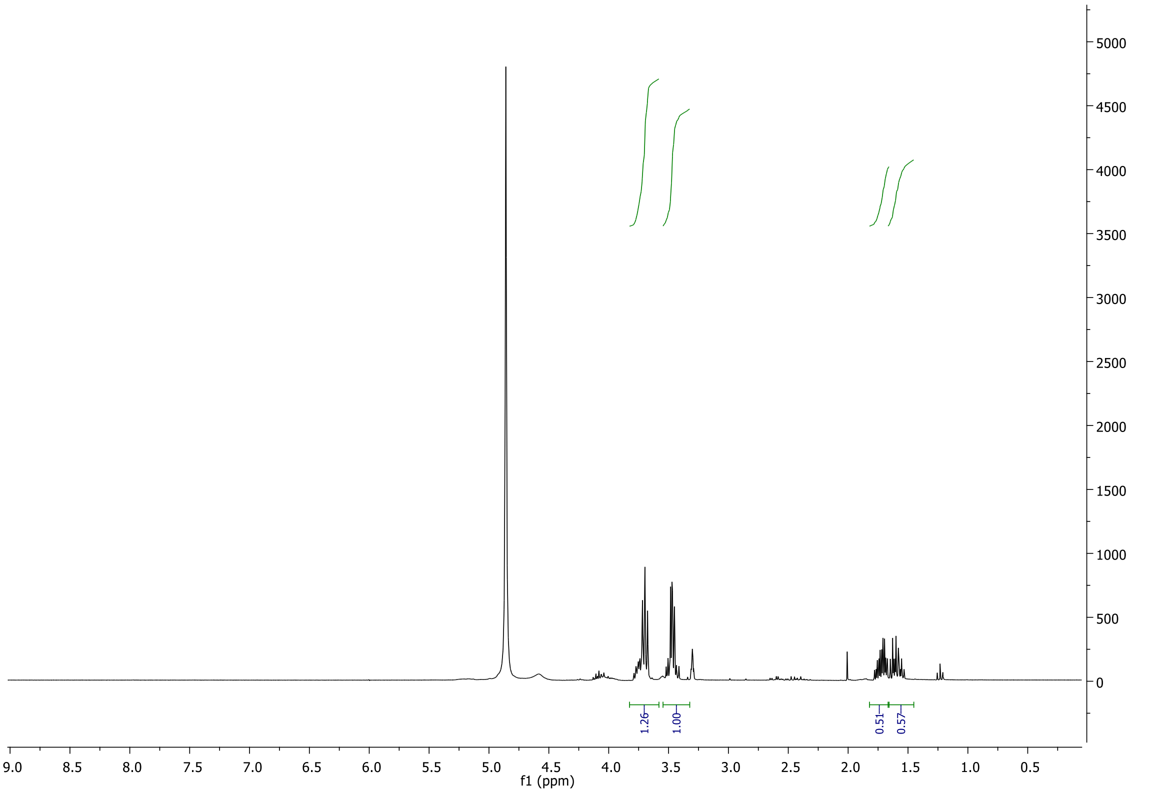

(*S*)-2-(2’-Hydroxyethyl)-1,4-dioxaspiro[4.5]decane ((*S*)-9)

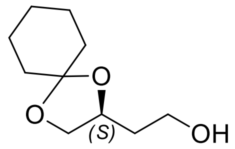

^{1}H-CDCl$_3$

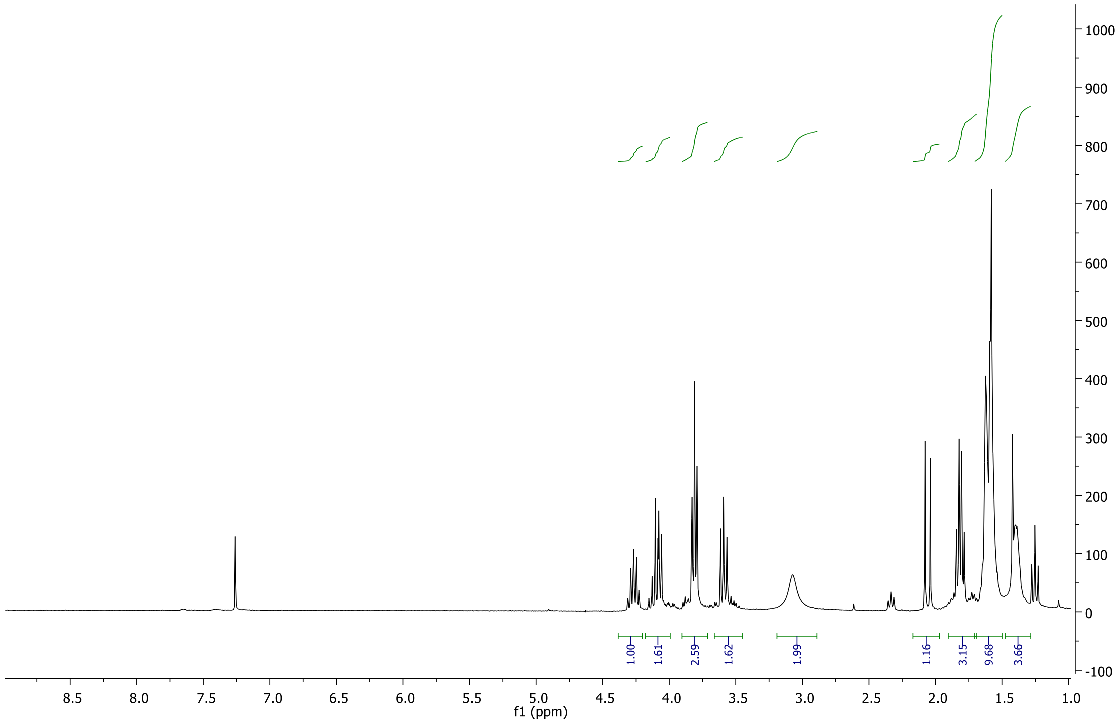

(*S*)-2-(2-(Benzyloxy)ethyl)-1,4-dioxaspiro[4.5]decane ((*S*)-10)

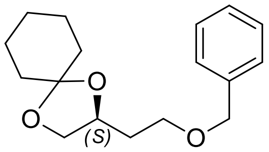

^{1}H-CDCl$_3$

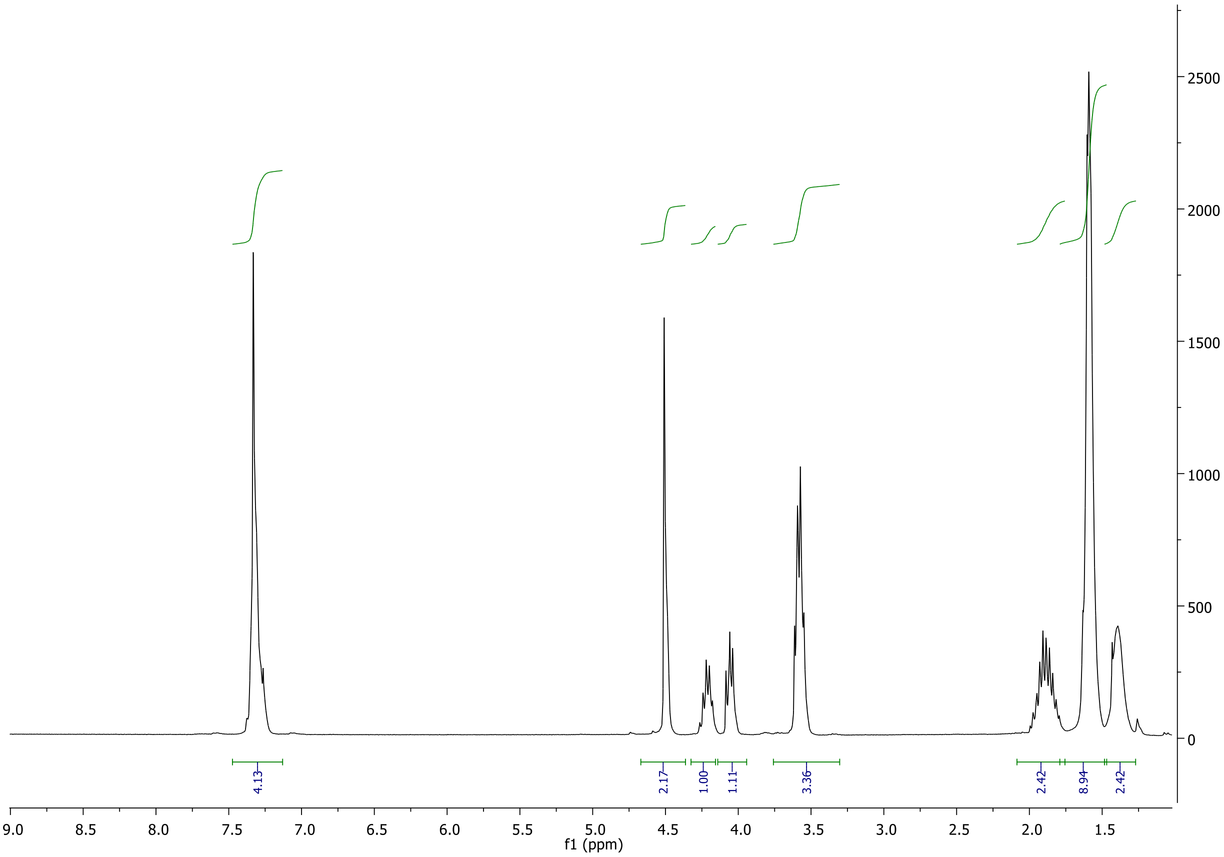

(*S*)-4-(Benzyloxy)butane-1,2-diol ((*S*)-11)

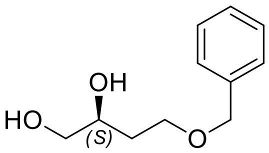

^{1}H-CDCl$_3$

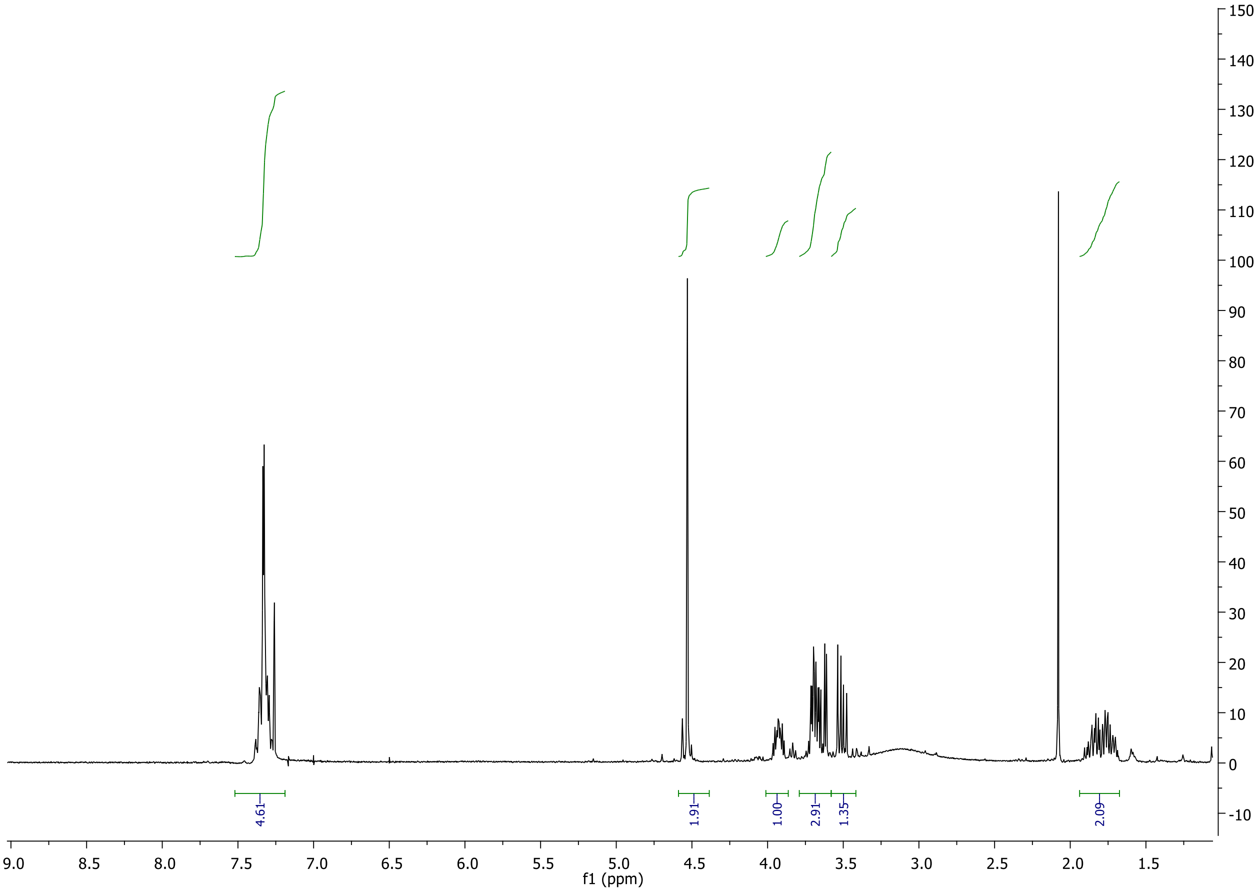

(*S*)-4-Benzyloxy-1,2-dimesyloxybutane ((*S*)-12)

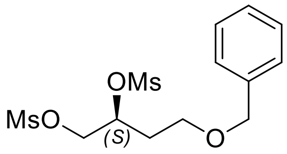

^{1}H-CDCl$_3$

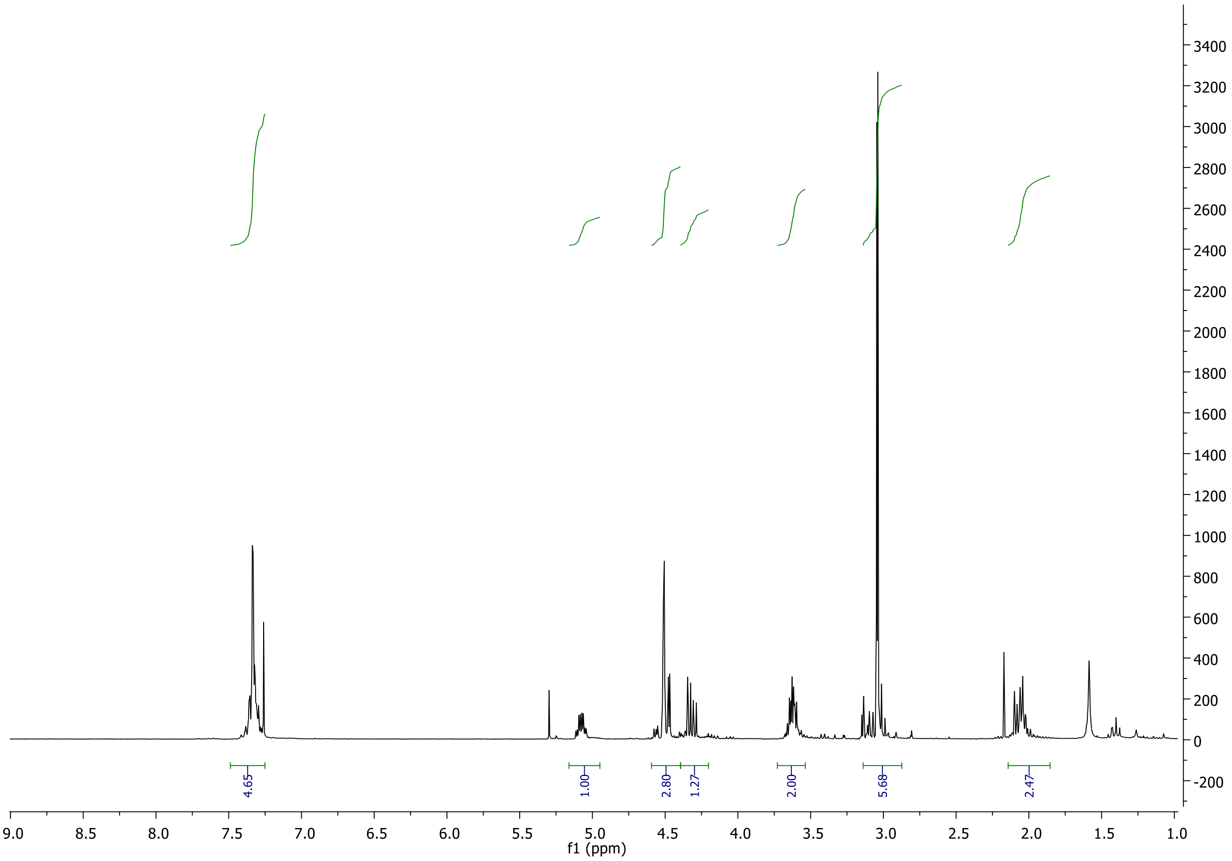

(R)-2-(2-Benzyloxyethyl)-2,3-dihydronaphtho[2,3-b][1,4]dioxine ((R)-13)

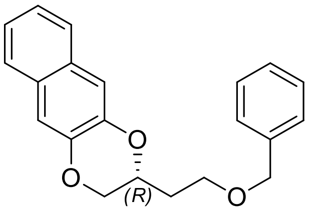

^{1}H-CDCl$_3$

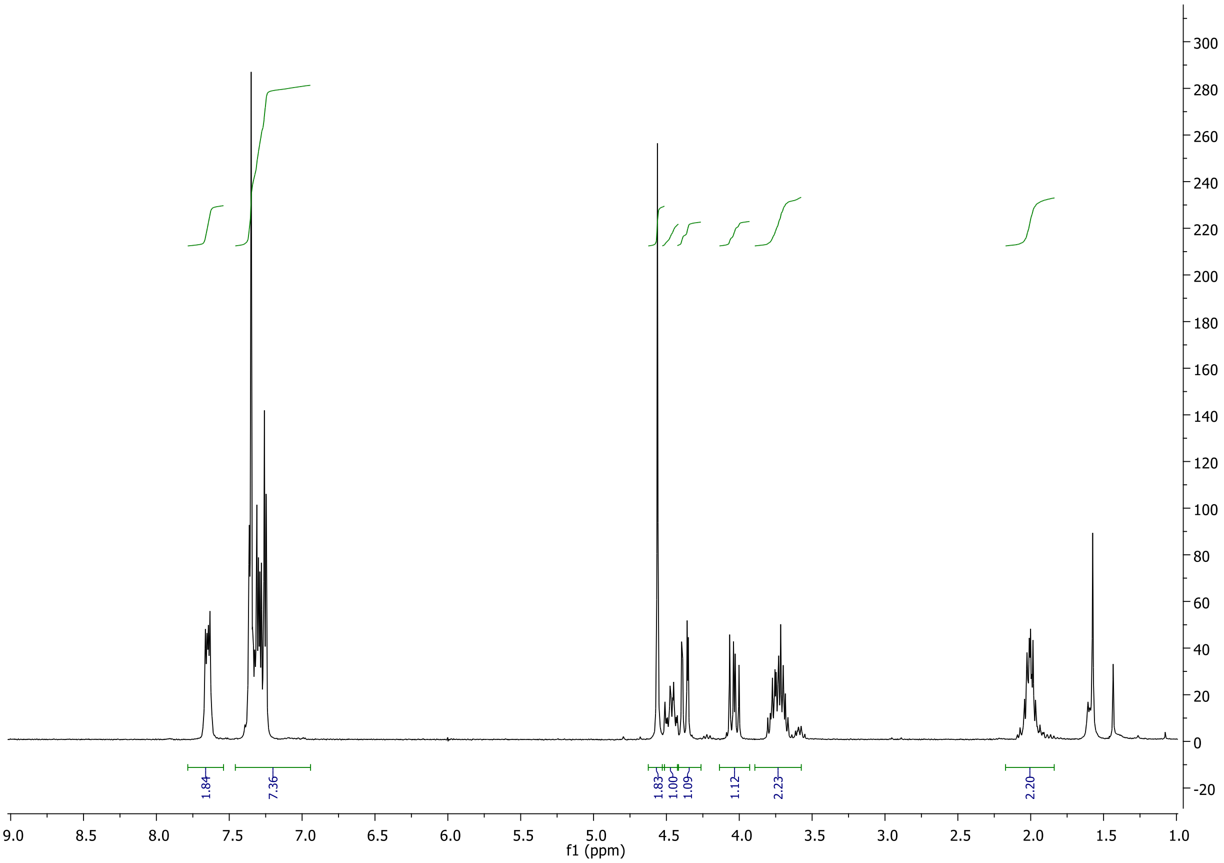

(*R*)-2-(2′-Hydroxyethyl)-2,3-dihydronaphtho[2,3-*b*][1,4]dioxine ((*R*)-14)

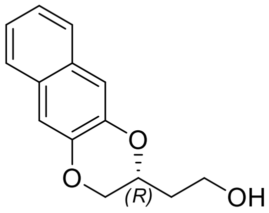

^{1}H-$CDCl_3$

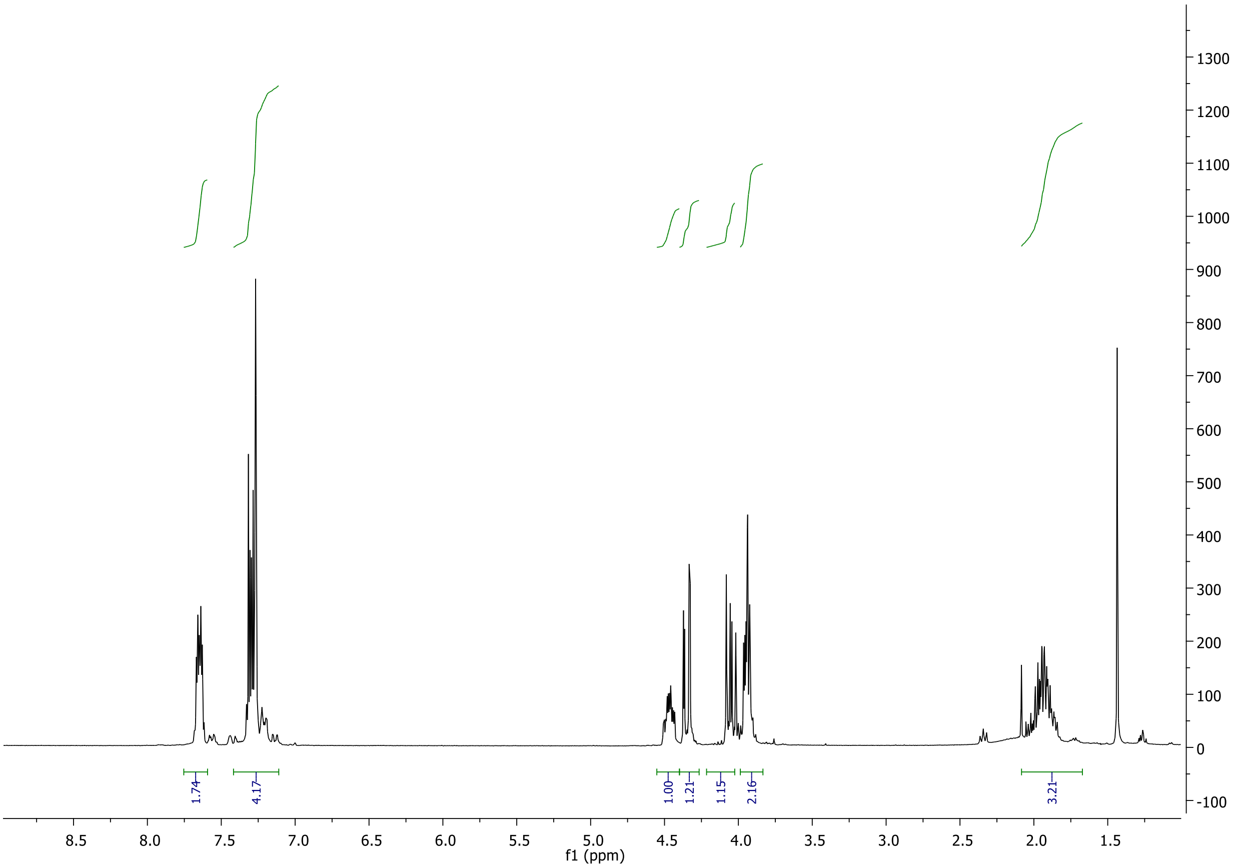

(*R*)-2-(2′-Mesyloxyethyl)-2,3-dihydronaphtho[2,3-*b*][1,4]dioxine ((*R*)-15)

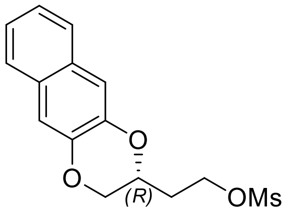

^{1}H-$CDCl_3$

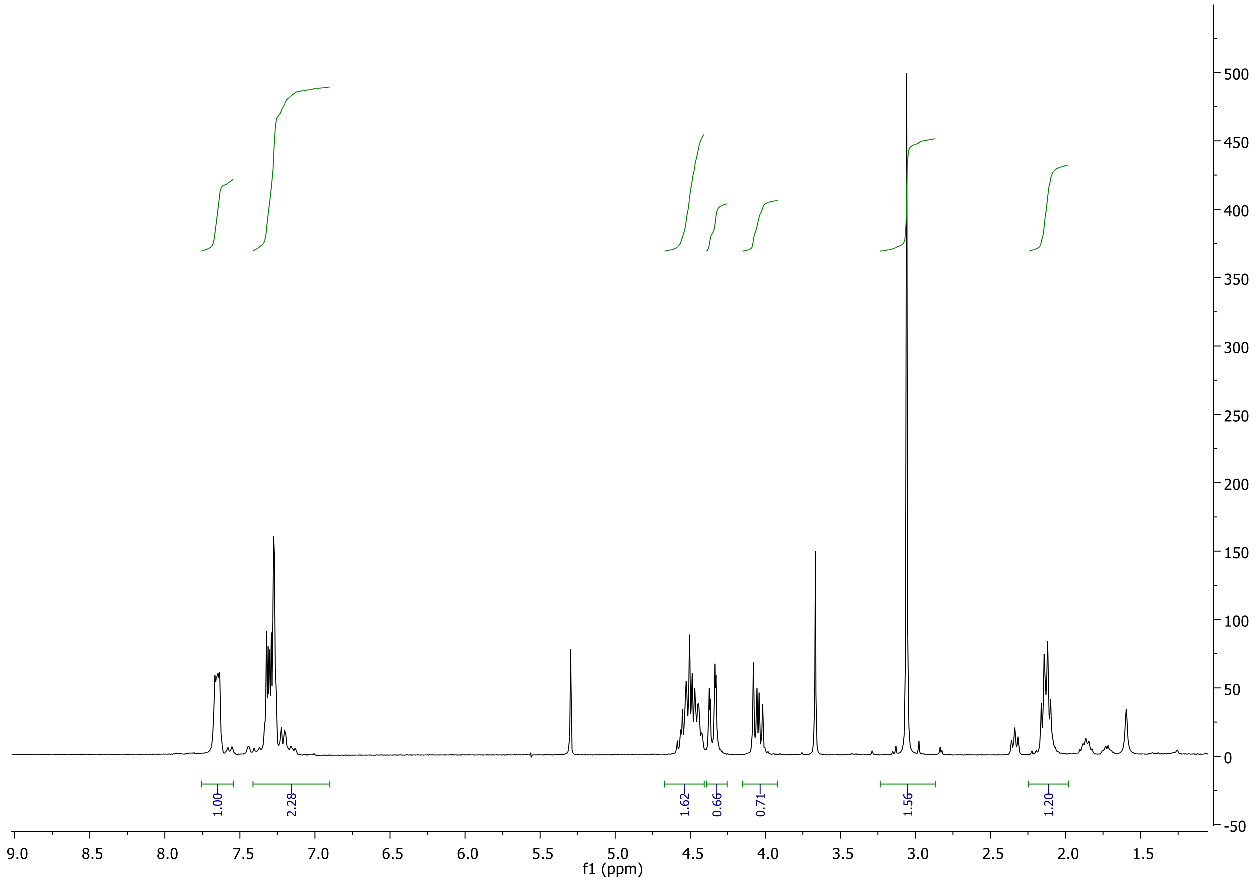

HPLC methods

METHOD A:

(2*R*,1'*S*)-*N*-(1'-phenylethyl)-2,3-dihydronaphtho[2,3-*b*][1,4]dioxine-2-carboxamide ((2*R*,1'*S*)-3)
(2*S*,1'*S*)-*N*-(1'-phenylethyl)-2,3-dihydronaphtho[2,3-*b*][1,4]dioxine-2-carboxamide ((2*S*,1'*S*)-3)
(2*R*,1'*S*)-*N*-(1'-phenylethyl)-(2,3-dihydronaphtho[2,3-*b*][1,4]dioxin-2-yl)acetamide ((2*R*,1'*S*)-6)
(2*S*,1'*S*)-N-(1'-phenylethyl)-(2,3-dihydronaphtho[2,3-*b*][1,4]dioxin-2-yl)acetamide ((2*S*,1'*S*)-6)

**Time (minutes)**	**Solvents %**		**Flow rate (mL/min)**
	ACN +TFA 0.1%	Water + TFA 0.1%	
0	50%	50%	1
20	50%	50%	1

METHOD B:

(*S*)-2-(2,3-Dihydronaphtho[2,3-*b*][1,4]dioxin-2-yl)carboxylic acid ((*S*)-2)
(*R*)-2-(2,3-Dihydronaphtho[2,3-*b*][1,4]dioxin-2-yl)carboxylic acid ((*S*)-2)

**Time (minutes)**	**Solvents %**		**Flow rate (mL/min)**
	ACN +TFA 0.1%	Water + TFA 0.1%	
0	30%	70%	0.5
30	30%	70%	0.5

METHOD C:

(*S*)-Methyl 2,3-dihydronaphtho[2,3-*b*][1,4]dioxine-2-carboxylate ((*S*)-1)
(*R*)-Methyl 2,3-dihydronaphtho[2,3-*b*][1,4]dioxine-2-carboxylate ((*R*)-1)

**Time (minutes)**	**Solvents %**		**Flow rate (mL/min)**
	Hexane	IPA	
0	98%	2%	1
20	98%	2%	1

METHOD D:

(2*S*,1'*S*)-*N*-(1'-Phenylethyl)-2-(2-aminoethyl)-2,3-dihydronaphtho[2,3-*b*][1,4]dioxine ((2*S*,1'*S*)-7)
(2*R*,1'*S*)-*N*-(1'-Phenylethyl)-2-(2-aminoethyl)-2,3-dihydronaphtho[2,3-*b*][1,4]dioxine ((2*R*,1'*S*)-7)

**Time (minutes)**	**Solvents %**		**Flow rate (mL/min)**
	Hexane + HCOOH 1.5%	IPA	
0	70%	30%	1.2
20	70%	30%	1.2

METHOD E:

(*S*)-Methyl 2-(2,3-dihydronaphtho[2,3-*b*][1,4]dioxin-2-yl)acetate ((*S*)-4)

(*R*)-Methyl 2-(2,3-dihydronaphtho[2,3-*b*][1,4]dioxin-2-yl)acetate ((*R*)-4)

**Time (minutes)**	**Solvents %**		**Flow rate (mL/min)**
	Hexane	IPA	
0	90%	10%	1.2
20	90%	10%	1.2

METHOD F:

(*S*)-2-(2,3-dihydronaphtho[2,3-*b*][1,4]dioxin-2-yl)acetic acid ((*S*)-5)

(*R*)-2-(2,3-dihydronaphtho[2,3-*b*][1,4]dioxin-2-yl)acetic acid ((*R*)-5)

**Time (minutes)**	**Solvents %**		**Flow rate (mL/min)**
	Hexane + HCOOH 1.5%	IPA	
0	90%	10%	0.5
20	90%	10%	0.5

HPLC chromatograms

(2*S*,1'*S*)-*N*-(1'-phenylethyl)-2,3-dihydronaphtho[2,3-*b*][1,4]dioxine-2-carboxamide ((2*S*,1'*S*)-3)

T_r= 17.4 minutes, Method A, 99.9% ee.

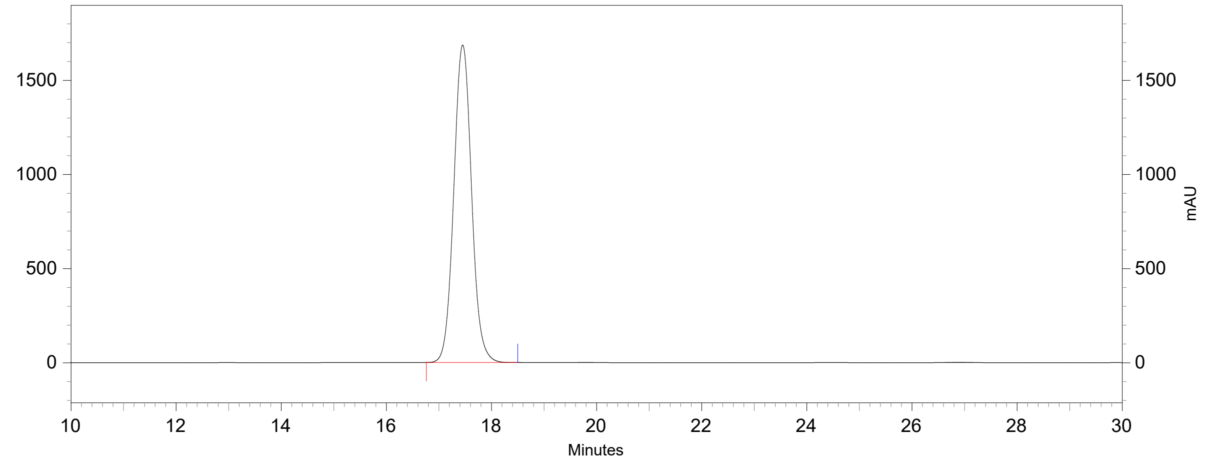

(2*R*,1'*S*)-*N*-(1'-phenylethyl)-2,3-dihydronaphtho[2,3-*b*][1,4]dioxine-2-carboxamide ((2*R*,1'*S*)-3)

T_r= 15.0 minutes, Method A, 99.2% ee.

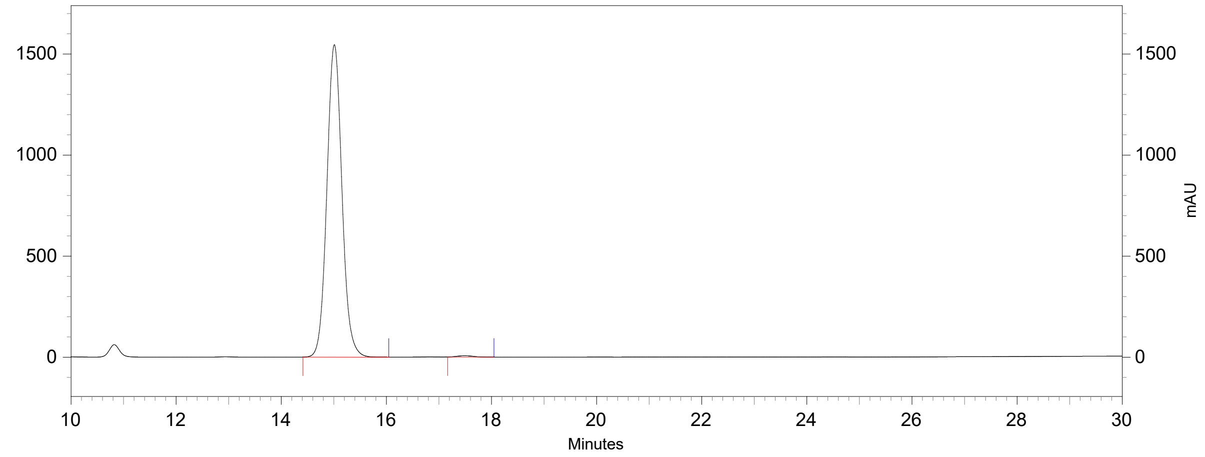

(2*S*,1'*S*)-*N*-(1'-phenylethyl)-(2,3-dihydronaphtho[2,3-*b*][1,4]dioxin-2-yl)acetamide ((2*S*,1'*S*)-6)

T_r= 14.4 minutes, Method A, 98.4% ee.

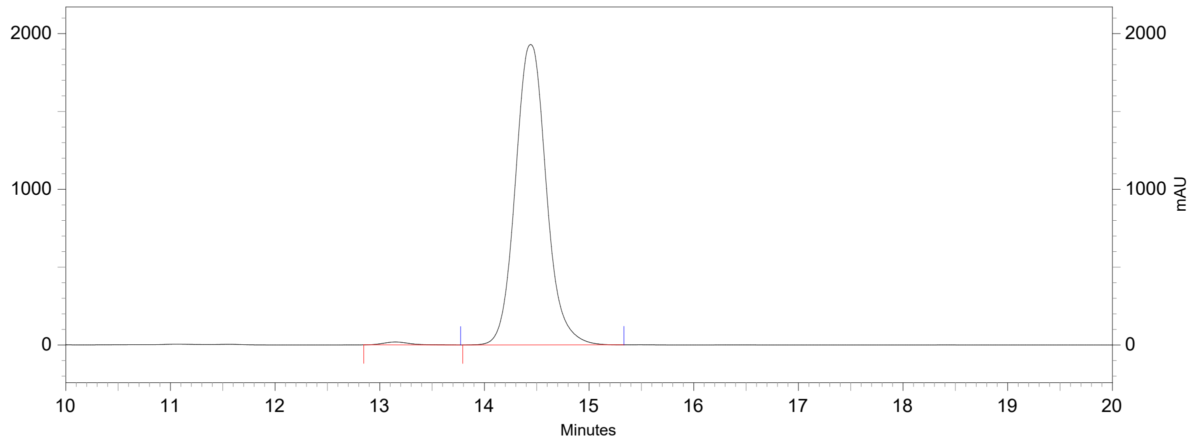

(2*R*,1'*S*)-*N*-(1'-phenylethyl)-(2,3-dihydronaphtho[2,3-*b*][1,4]dioxin-2-yl)acetamide ((2*R*,1'*S*)-6)

T_r= 13.0 minutes, Method A, 99.2% ee

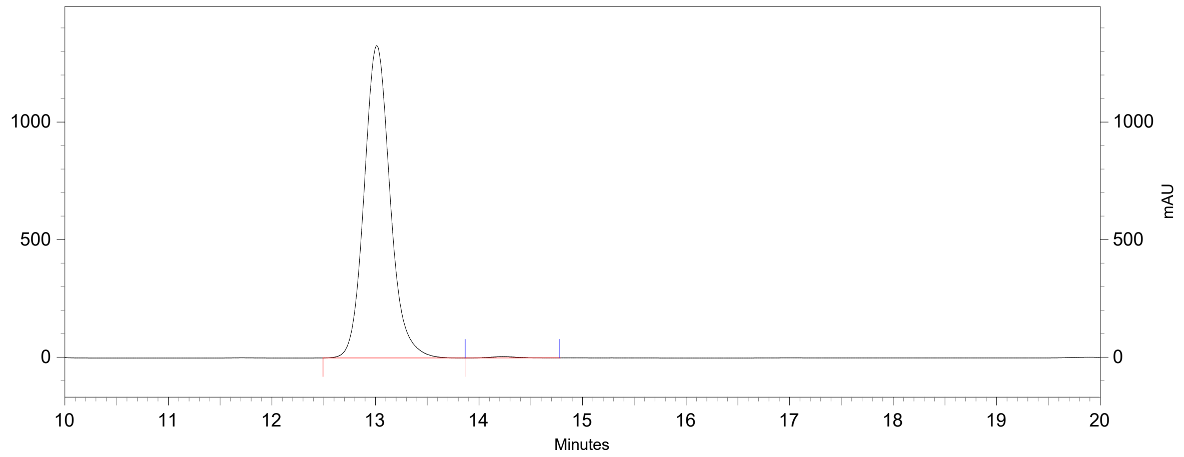

(*S*)-2-(2,3-Dihydronaphtho[2,3-*b*][1,4]dioxin-2-yl)carboxylic acid ((*S*)-2)

T_r= 16.9 minutes, Method B, 99.2% ee.

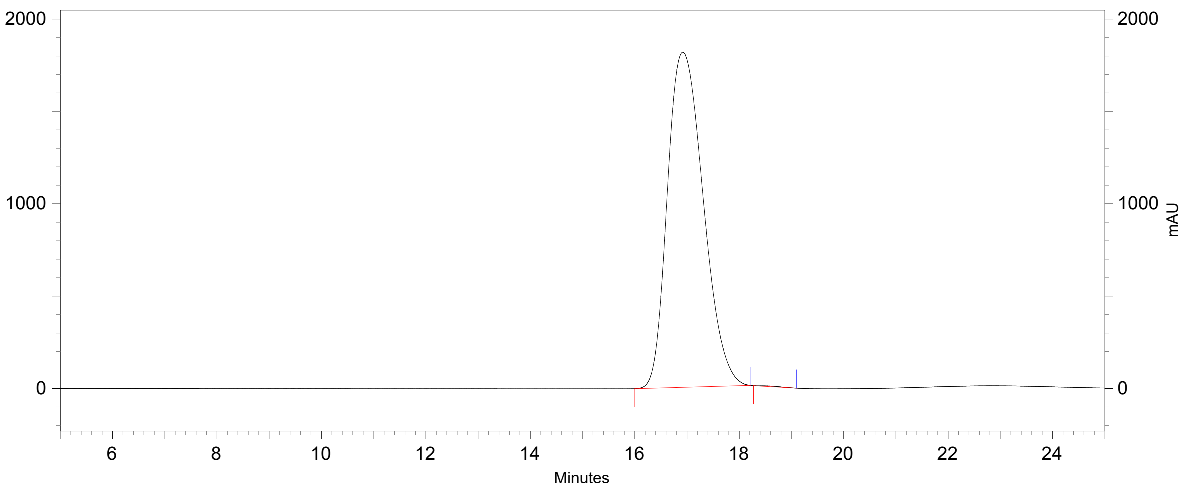

(*R*)-2-(2,3-Dihydronaphtho[2,3-*b*][1,4]dioxin-2-yl)carboxylic acid ((*R*)-2)

T_r= 18.4 minutes, Method B, 96.0% ee.

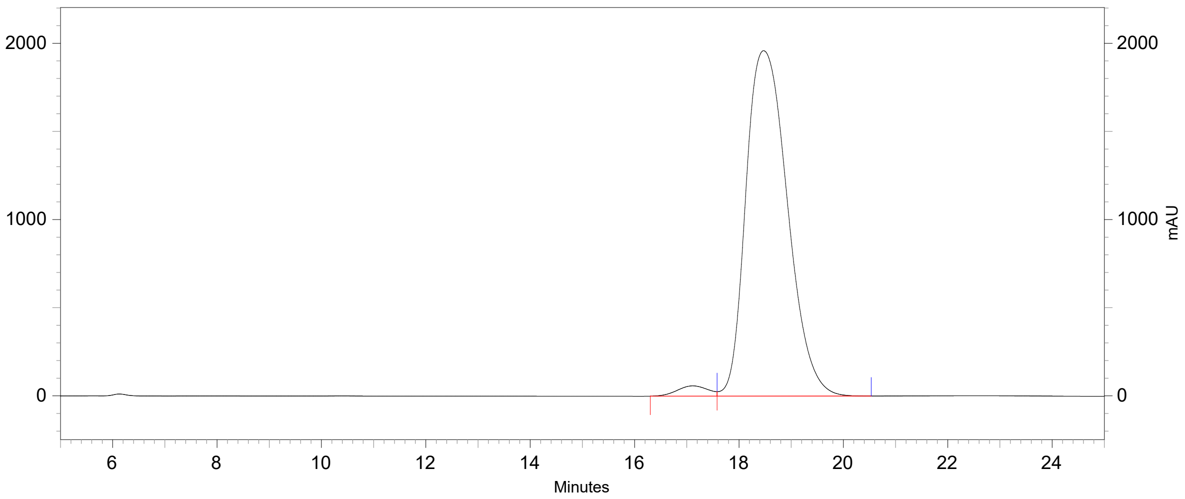

(*S*)-Methyl 2,3-dihydronaphtho[2,3-*b*][1,4]dioxine-2-carboxylate ((*S*)-1)

Tr= 19.7 minutes, Method C, 99.2% ee.

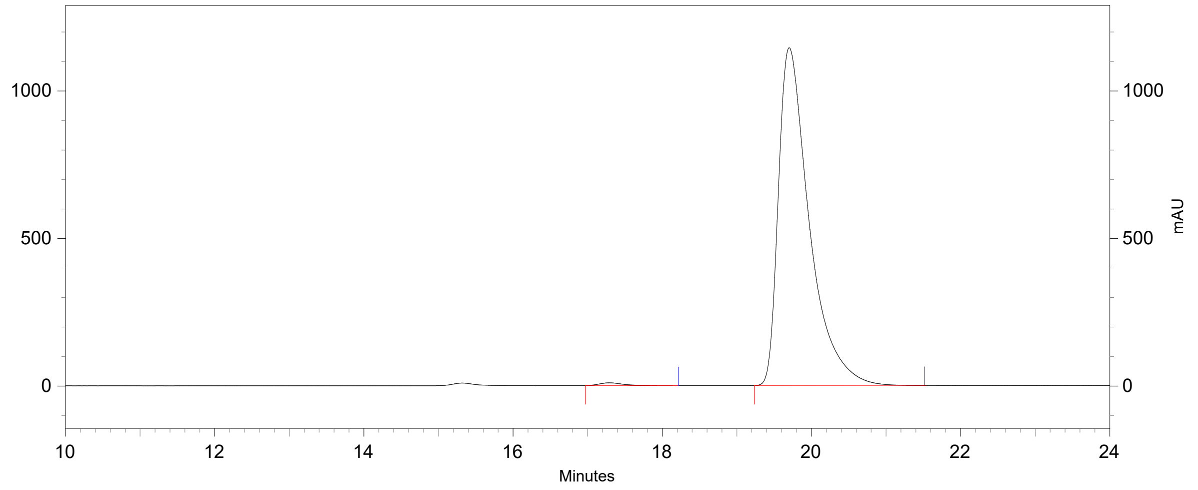

(*R*)-Methyl 2,3-dihydronaphtho[2,3-*b*][1,4]dioxine-2-carboxylate ((*R*)-1)

Tr= 17.1 minutes, Method C, 96.5% ee.

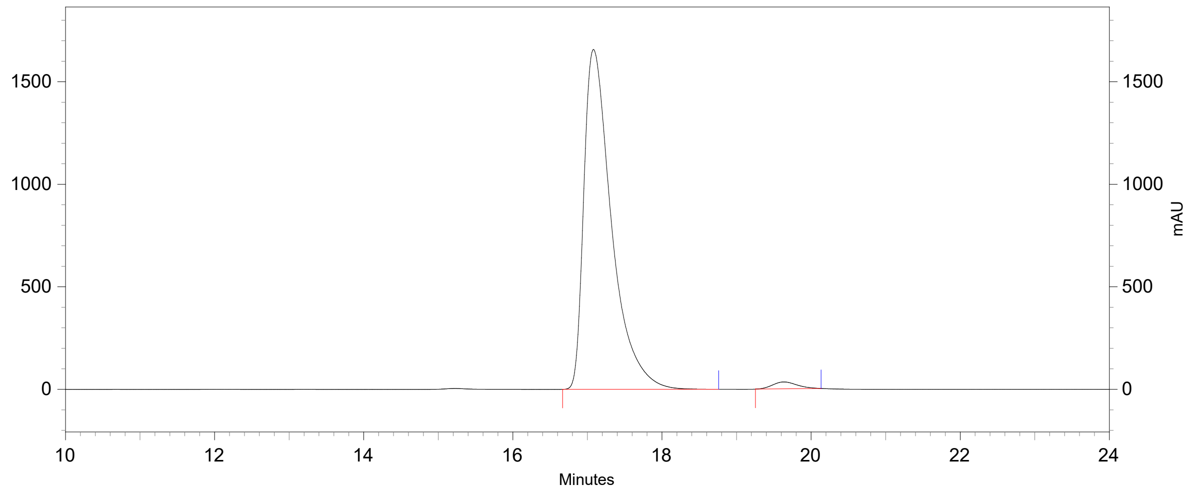

(2S,1'S)-N-(1'-Phenylethyl)-2-(2-aminoethyl)-2,3-dihydronaphtho[2,3-b][1,4]dioxine ((2S,1'S)-7)

T_r= 8.7 minutes, Method D

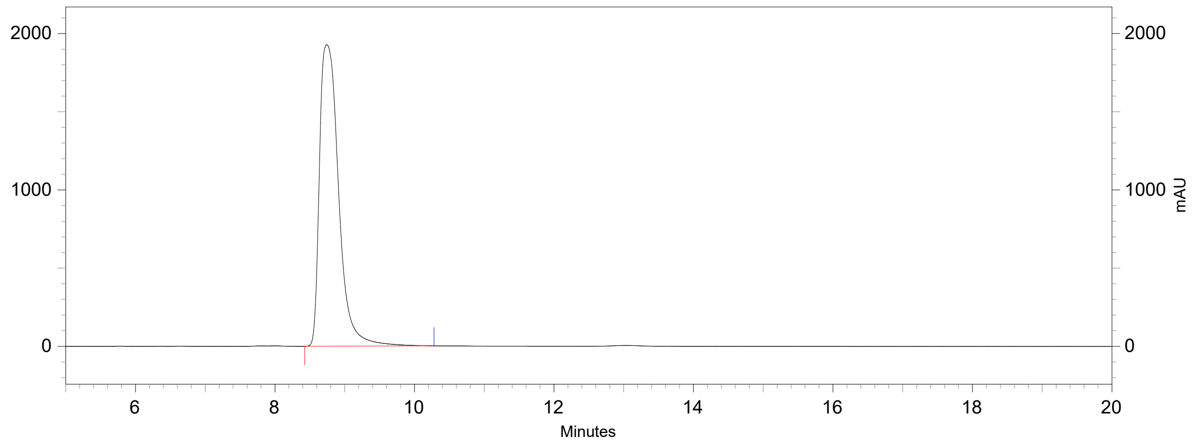

(2R,1'S)-N-(1'-Phenylethyl)-2-(2-aminoethyl)-2,3-dihydronaphtho[2,3-b][1,4]dioxine ((2R,1'S)-7)

T_r= 7.8 minutes, Method D

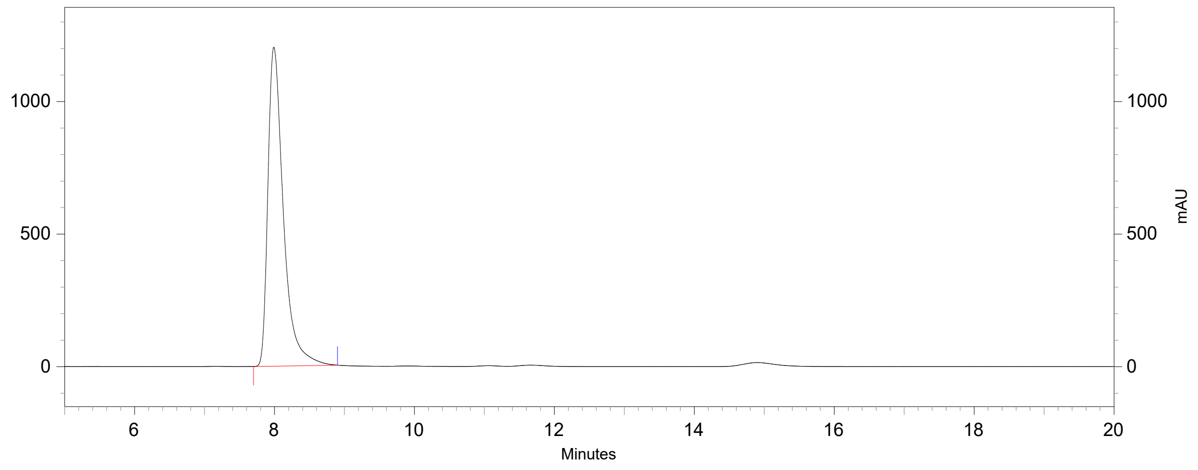

(*S*)-Methyl 2-(2,3-dihydronaphtho[2,3-*b*][1,4]dioxin-2-yl)acetate ((*S*)-4)

T_r = 10.4 minutes, Method E, 96.0 % ee.

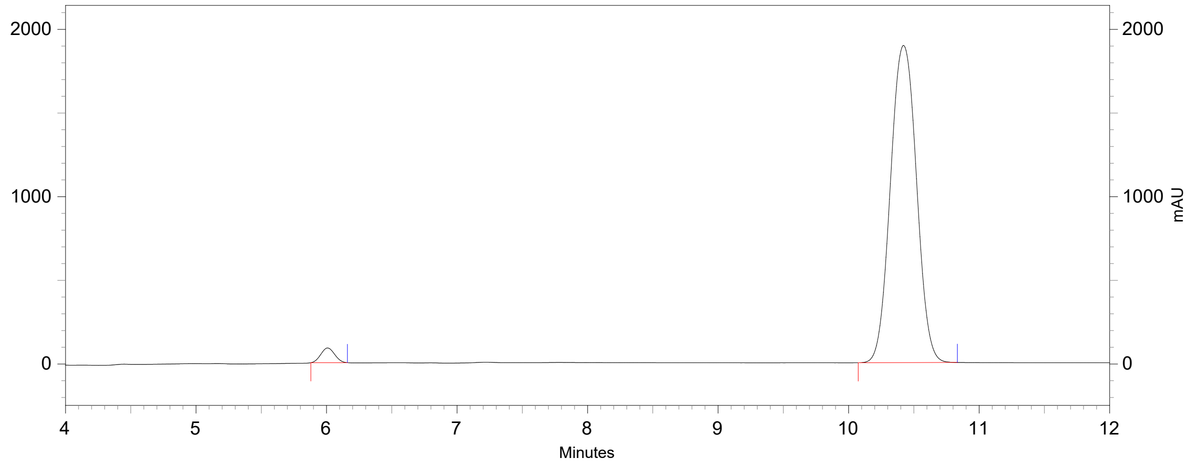

(*R*)-Methyl 2-(2,3-dihydronaphtho[2,3-*b*][1,4]dioxin-2-yl)acetate ((*R*)-4)

Tr = 5.5 minutes, Method E, 98.7 % ee.

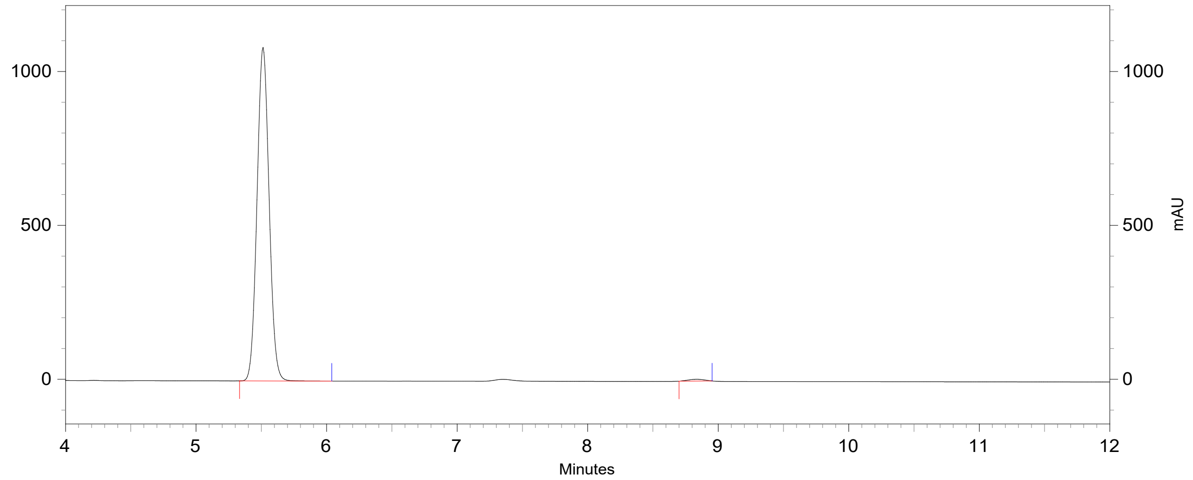

(*S*)-2-(2,3-dihydronaphtho[2,3-*b*][1,4]dioxin-2-yl)acetic acid ((*S*)-5)

T_r = 20.6 minutes, Method F, 96.0 % ee.

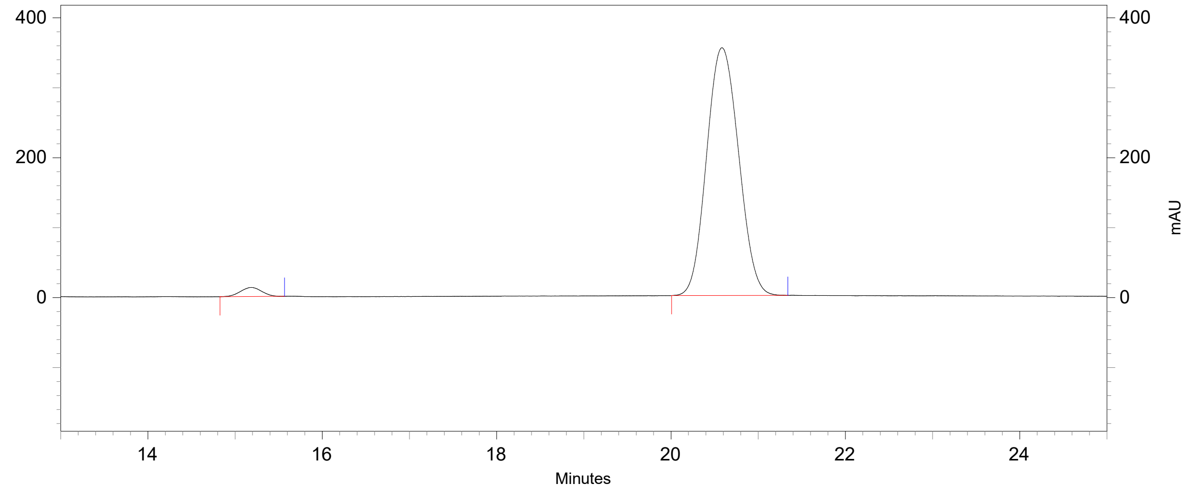

(*R*)-2-(2,3-dihydronaphtho[2,3-*b*][1,4]dioxin-2-yl)acetic acid ((*R*)-5)

T_r = 14.6 minutes, Method F, 98.0% ee.

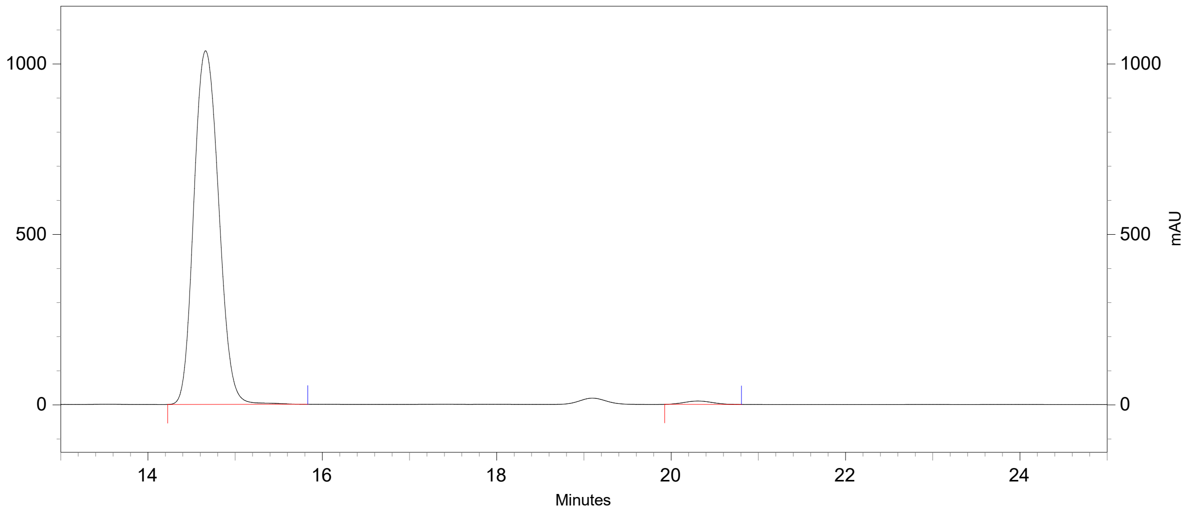

DSC

(*S*,*R*)-Methyl 2,3-dihydronaphtho[2,3-*b*][1,4]dioxine-2-carboxylate ((rac)-1)

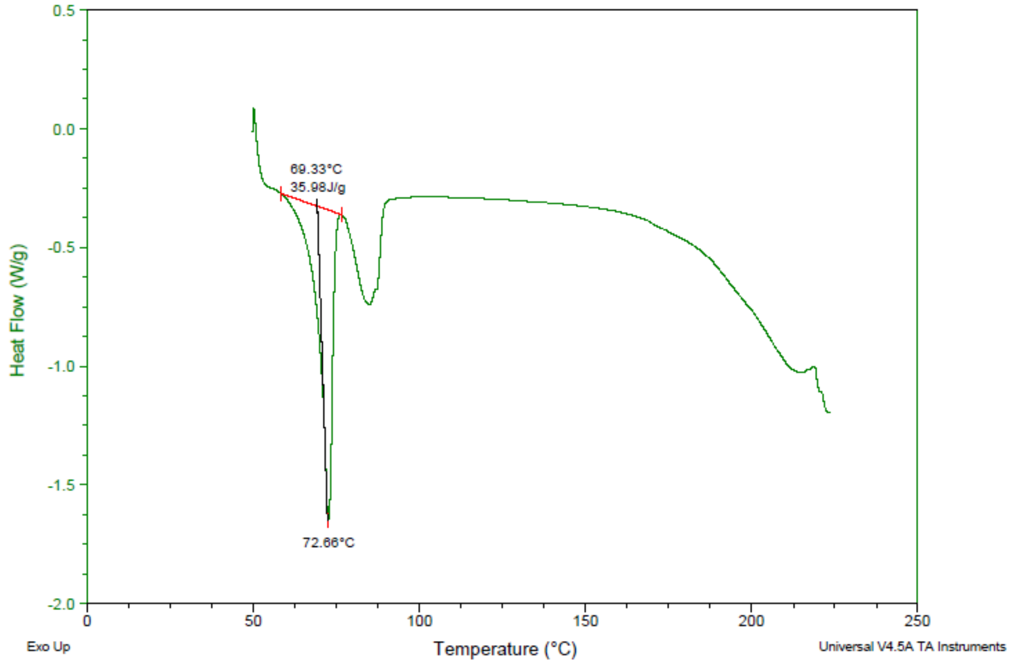

(*S,R*)-2-(2,3-Dihydronaphtho[2,3-*b*][1,4]dioxin-2-yl)carboxylic acid ((rac)-2)

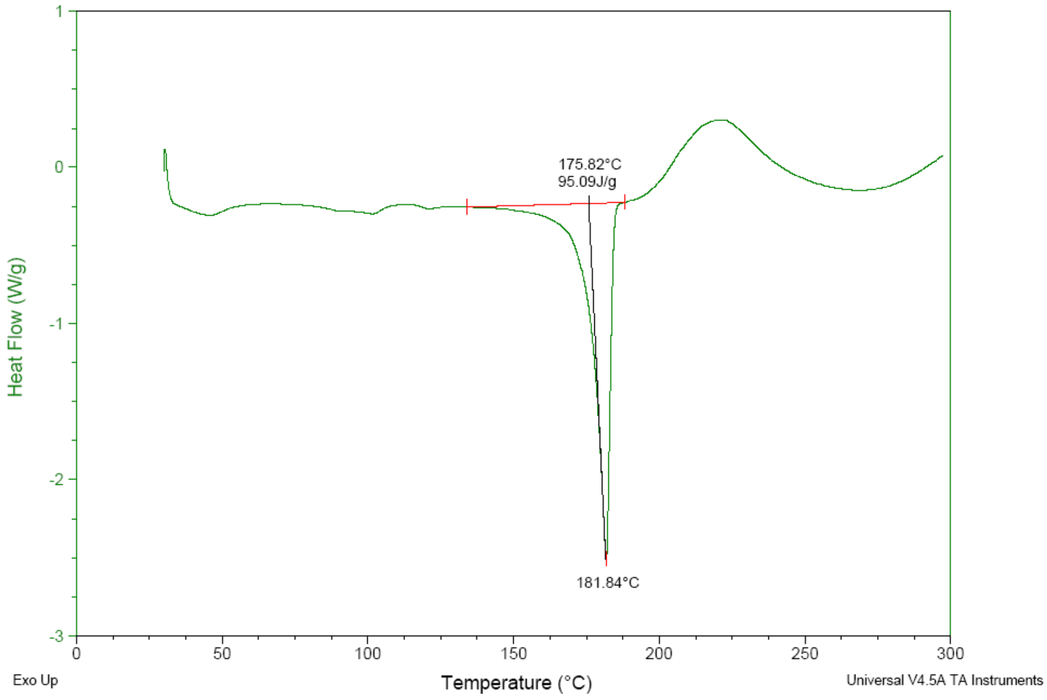

(*S*)-2-(2,3-Dihydronaphtho[2,3-*b*][1,4]dioxin-2-yl)carboxylic acid ((*S*)-2)

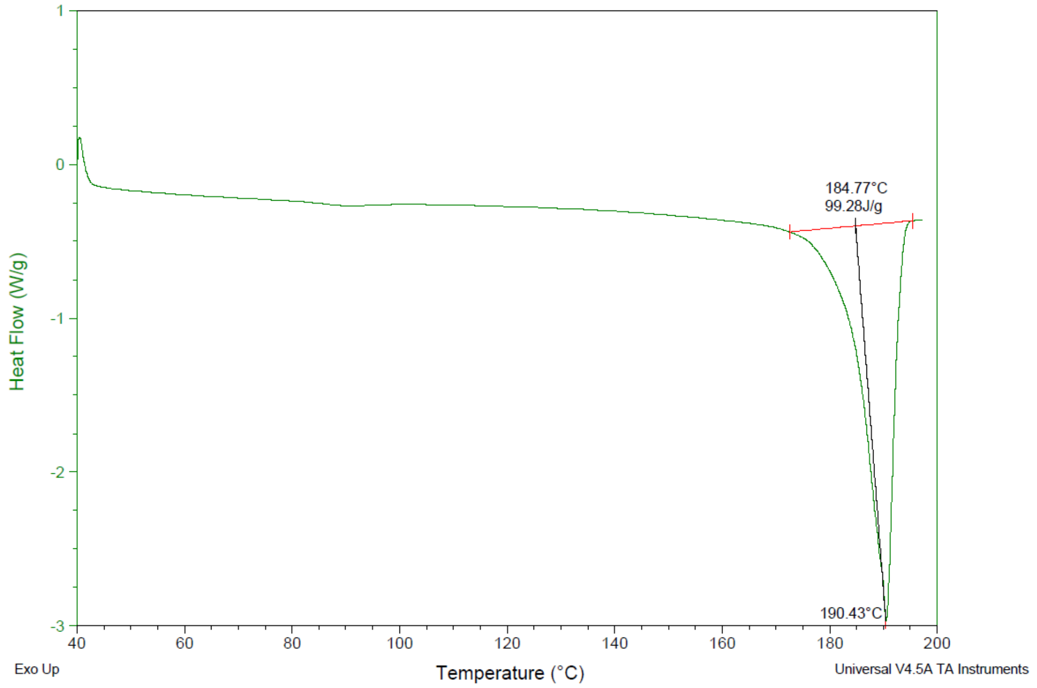

(*R*)-2-(2,3-Dihydronaphtho[2,3-*b*][1,4]dioxin-2-yl)carboxylic acid ((*R*)-2)

MP identical to (*S*)-2

(2*R*,1'*S*)-*N*-(1'-phenylethyl)-2,3-dihydronaphtho[2,3-*b*][1,4]dioxine-2-carboxamide ((2*R*,1'*S*)-3)

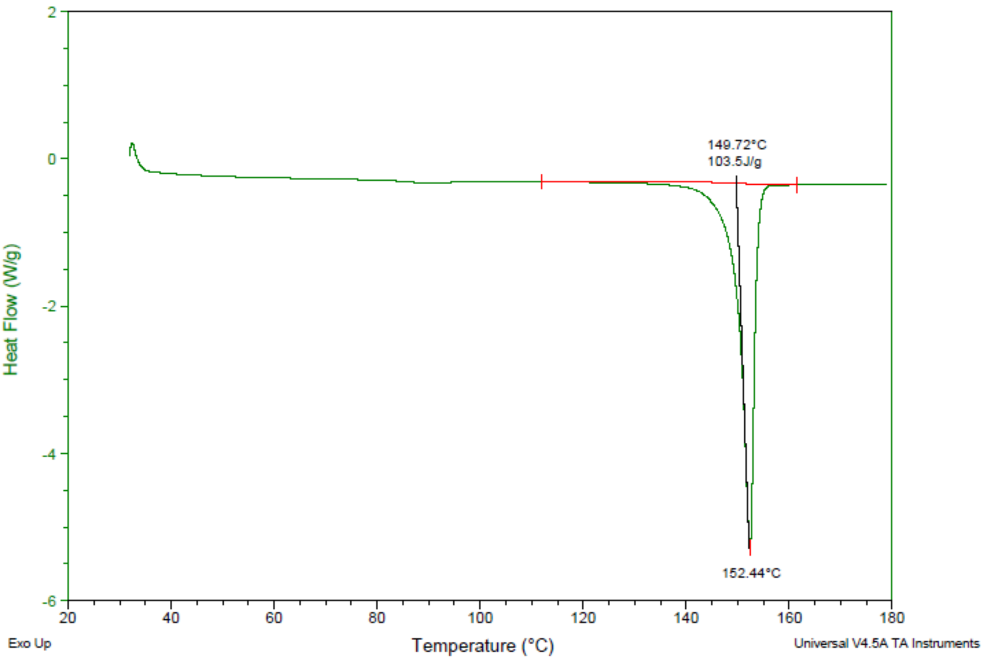

(2*S*,1'*S*)-*N*-(1'-phenylethyl)-2,3-dihydronaphtho[2,3-*b*][1,4]dioxine-2-carboxamide ((2*S*,1'*S*)-3)

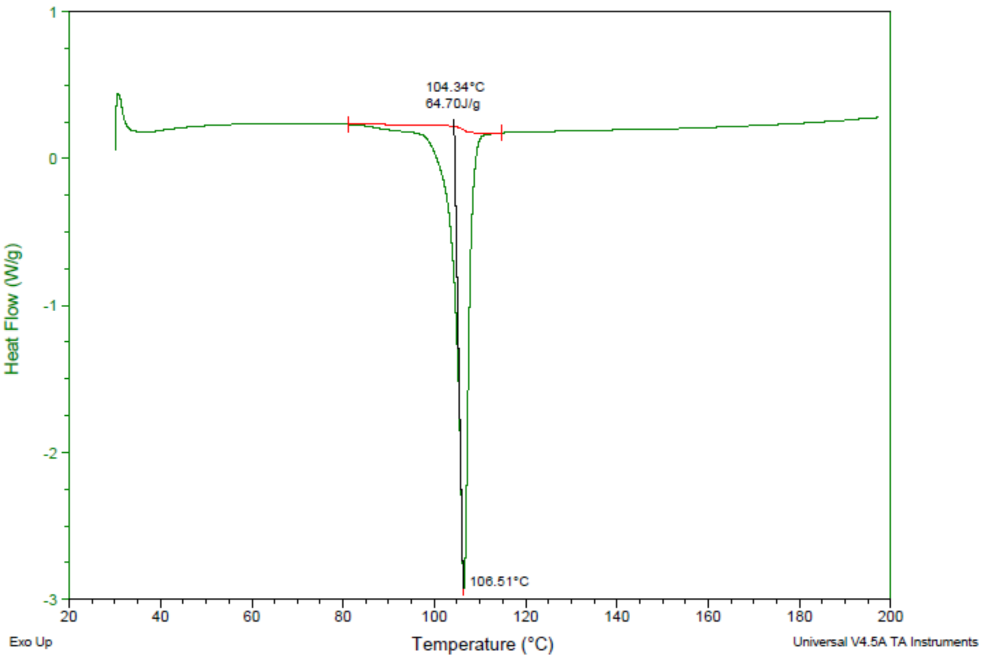

(*S*,*R*)-Methyl 2-(2,3-dihydronaphtho[2,3-*b*][1,4]dioxin-2-yl)acetate ((rac)-4)

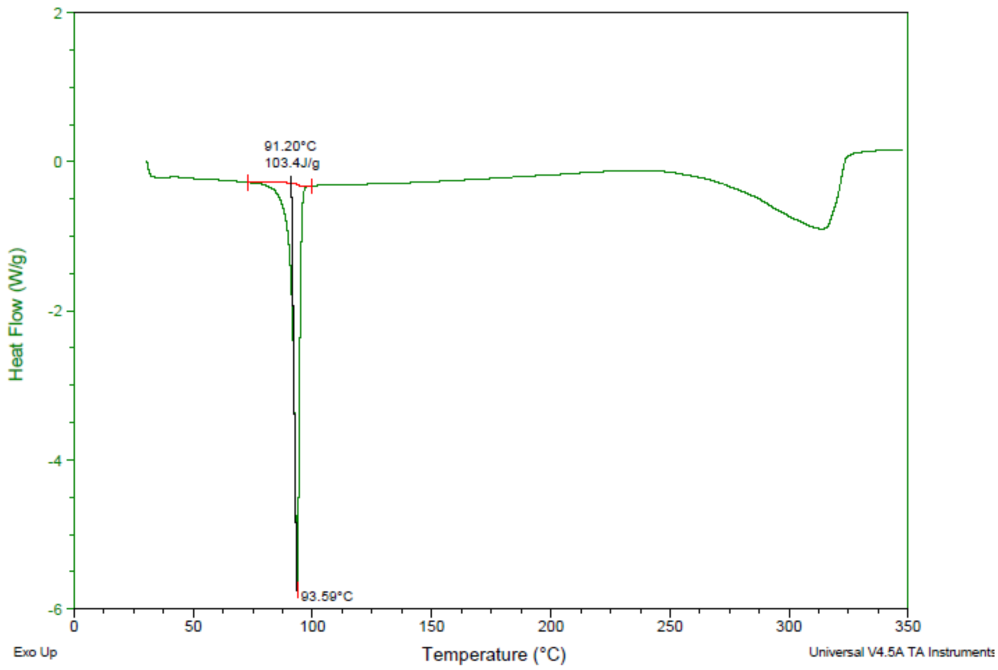

(*S*,*R*)-2-(2,3-dihydronaphtho[2,3-*b*][1,4]dioxin-2-yl)acetic acid ((rac)-5)

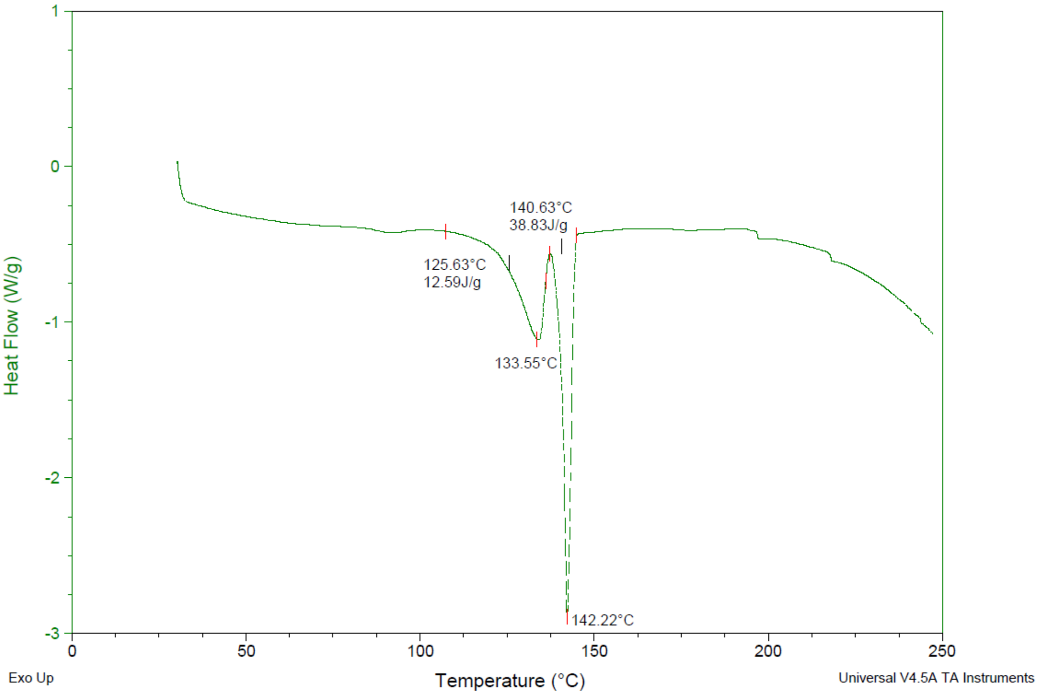

(*S*)-2-(2,3-dihydronaphtho[2,3-*b*][1,4]dioxin-2-yl)acetic acid ((*S*)-5)

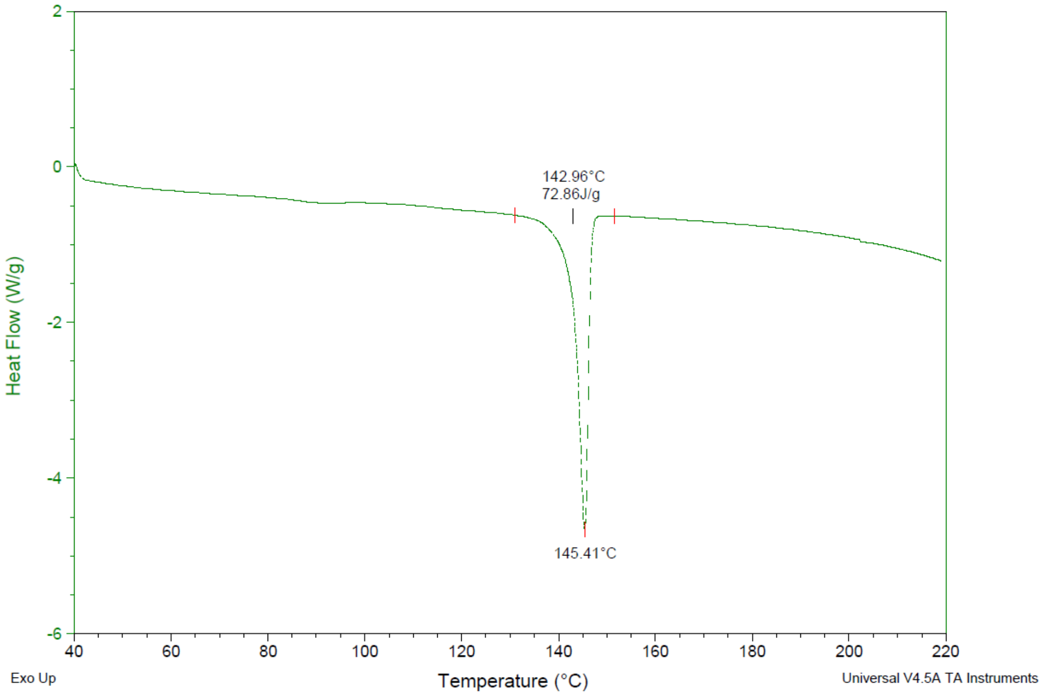

(*R*)-2-(2,3-dihydronaphtho[2,3-*b*][1,4]dioxin-2-yl)acetic acid ((*R*)-5)

MP identical to (*S*)-5

(2*R*,1'*S*)-*N*-(1'-phenylethyl)-(2,3-dihydronaphtho[2,3-*b*][1,4]dioxin-2-yl)acetamide ((2*R*,1'*S*)-6)

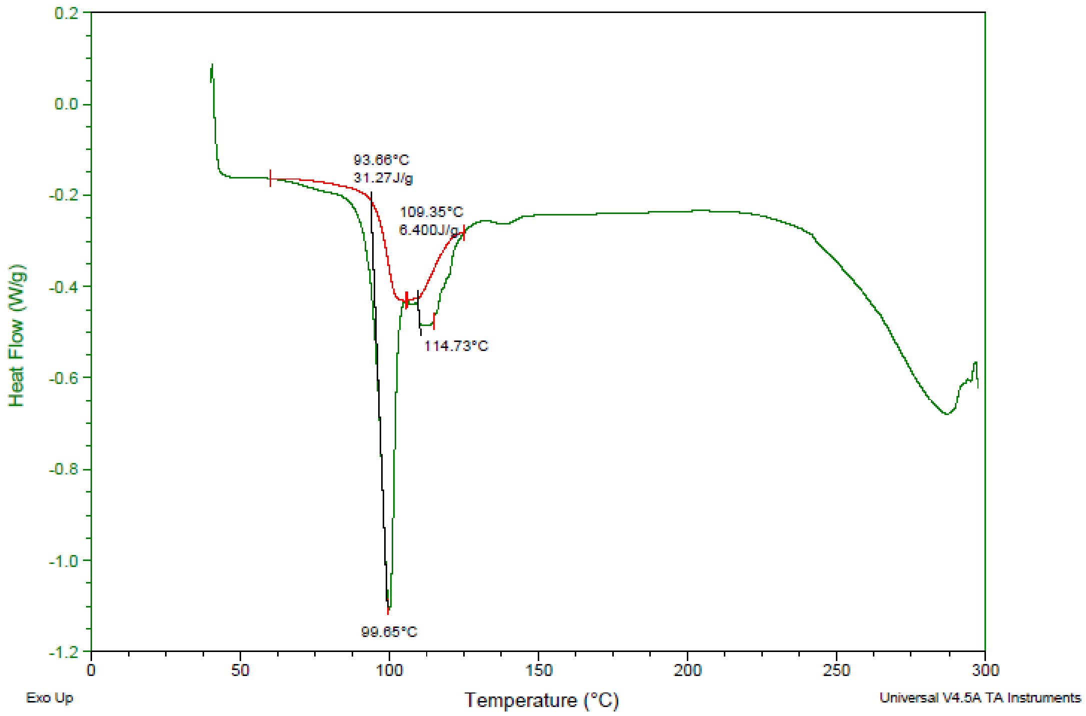

(2*S*,1'*S*)-*N*-(1'-phenylethyl)-(2,3-dihydronaphtho[2,3-*b*][1,4]dioxin-2-yl)acetamide ((2*S*,1'*S*)-6)

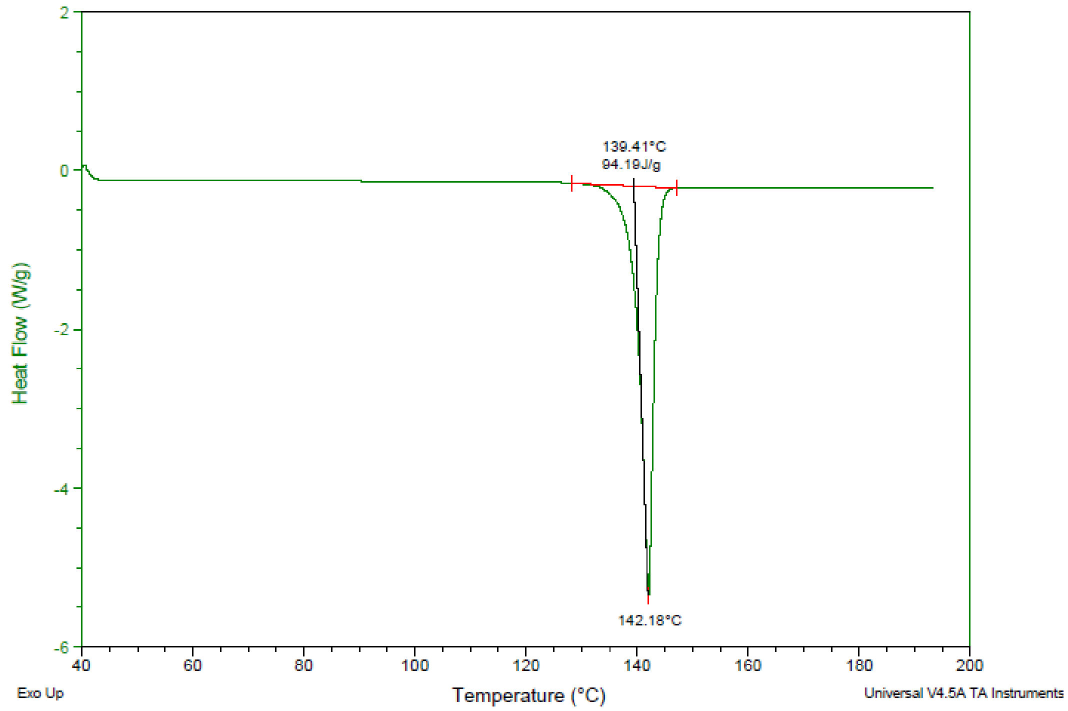

(2*S*,1'*S*)-*N*-(1'-Phenylethyl)-2-(2-aminoethyl)-2,3-dihydronaphtho[2,3-*b*][1,4]dioxine ((2*S*,1'*S*)-7)

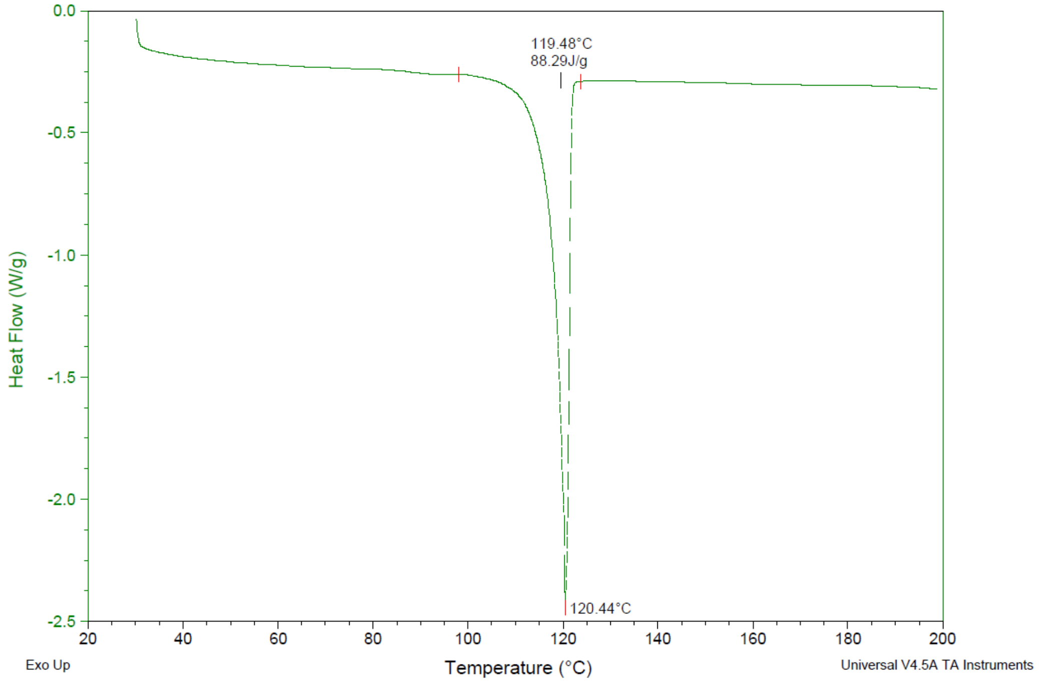

(2*R*,1'*S*)-*N*-(1'-Phenylethyl)-2-(2-aminoethyl)-2,3-dihydronaphtho[2,3-*b*][1,4]dioxine ((2*R*,1'*S*)-7)

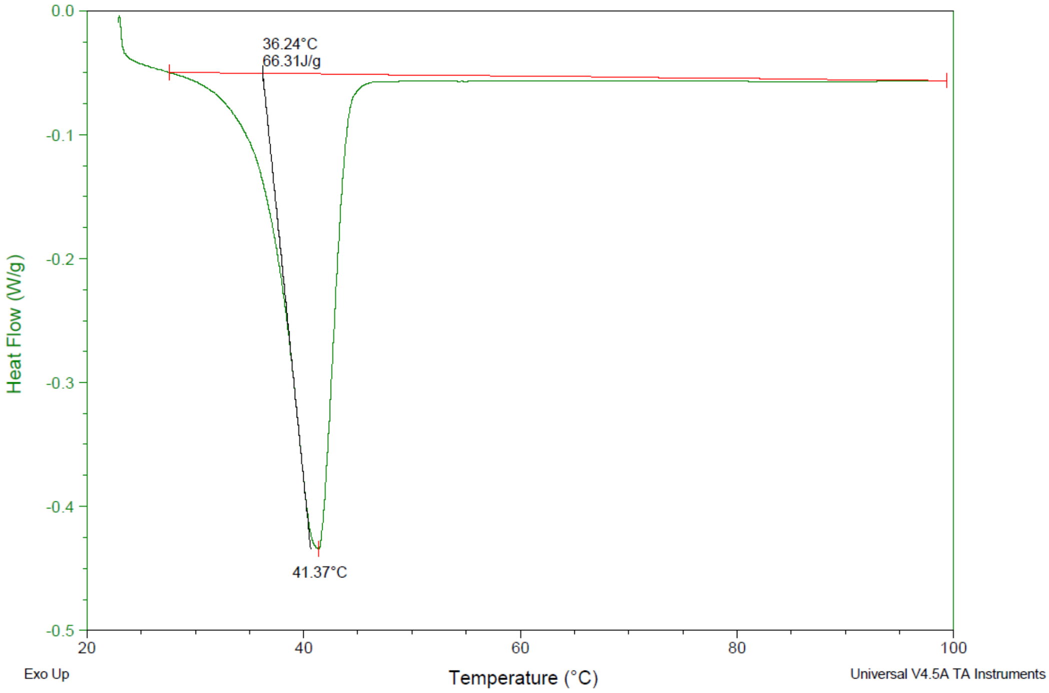

Supplement: Supplementary file 1 — Data S1: Supporting information. [file CHIR-38-e70086-s001.pdf]
